# Soilless Cultivated Halophyte Plants: Volatile, Nutritional, Phytochemical, and Biological Differences

**DOI:** 10.3390/antiox12061161

**Published:** 2023-05-26

**Authors:** Sheila C. Oliveira-Alves, Fábio Andrade, João Sousa, Andreia Bento-Silva, Bernardo Duarte, Isabel Caçador, Miguel Salazar, Elsa Mecha, Ana Teresa Serra, Maria Rosário Bronze

**Affiliations:** 1 iBET, Instituto de Biologia Experimental e Tecnológica, Apartado 12, 2781-901 Oeiras, Portugal; sheila.alves@itqb.unl.pt (S.C.O.-A.); f.andrade@campus.ul.pt (F.A.); joao.sousa@staff.uma.pt (J.S.); emecha@itqb.unl.pt (E.M.); tserra@ibet.pt (A.T.S.); 2 ITQB-NOVA, Instituto de Tecnologia Química e Biológica António Xavier, Universidade Nova de Lisboa, Av. da República, 2780-157 Oeiras, Portugal; 3 Faculdade de Farmácia, Universidade de Lisboa, Av. Gama Pinto, 1649-003 Lisboa, Portugal; abentosilva@ff.ulisboa.pt; 4 MARE—Marine and Environmental Sciences Centre & ARNET–Aquatic Research Network Associated Laboratory, Faculdade de Ciências da Universidade de Lisboa, Campo Grande, 1749-016 Lisbon, Portugal; baduarte@fc.ul.pt (B.D.); micacador@fc.ul.pt (I.C.); 5 Departamento de Biologia Vegetal, Faculdade de Ciências, Universidade de Lisboa, Campo Grande, 1749-016 Lisboa, Portugal; 6 Riafresh, Sítio do Besouro, CX 547-B, 8005-421 Faro, Portugal; miguel.salazar@riafresh.com; 7 MED—Mediterranean Institute for Agriculture, Environment and Development, Universidade do Algarve, Campus de Gambelas, 8005-139 Faro, Portugal

**Keywords:** salt-tolerating plants, soilless agriculture, phenolics, fatty acids, antihypertensive activity

## Abstract

The use of halophyte plants appears as a potential solution for degraded soil, food safety, freshwater scarcity, and coastal area utilization. These plants have been considered an alternative crop soilless agriculture for sustainable use of natural resources. There are few studies carried out with cultivated halophytes using a soilless cultivation system (SCS) that report their nutraceutical value, as well as their benefits on human health. The objective of this study was to evaluate and correlate the nutritional composition, volatile profile, phytochemical content, and biological activities of seven halophyte species cultivated using a SCS (*Disphyma crassifolium* L., *Crithmum maritimum* L., *Inula crithmoides* L., *Mesembryanthemum crystallinum* L., *Mesembryanthemum nodiflorum* L., *Salicornia ramosissima* J. Woods, and *Sarcocornia fruticosa* (Mill.) A. J. Scott.). Among these species, results showed that *S. fruticosa* had a higher content in protein (4.44 g/100 g FW), ash (5.70 g/100 g FW), salt (2.80 g/100 g FW), chloride (4.84 g/100 g FW), minerals (Na, K, Fe, Mg, Mn, Zn, Cu), total phenolics (0.33 mg GAE/g FW), and antioxidant activity (8.17 µmol TEAC/g FW). Regarding the phenolic classes, *S. fruticosa* and *M. nodiflorum* were predominant in the flavonoids, while *M. crystallinum*, *C. maritimum,* and *S. ramosissima* were in the phenolic acids. Moreover, *S. fruticosa, S. ramosissima, M. nodiflorum*, *M. crystallinum,* and *I. crithmoides* showed ACE-inhibitory activity, an important target control for hypertension. Concerning the volatile profile, *C. maritimum*, *I. crithmoides,* and *D. crassifolium* were abundant in terpenes and esters, while *M. nodiflorum*, *S. fruticosa,* and *M. crystallinum* were richer in alcohols and aldehydes, and *S. ramosissima* was richer in aldehydes. Considering the environmental and sustainable roles of cultivated halophytes using a SCS, these results indicate that these species could be considered an alternative to conventional table salt, due to their added nutritional and phytochemical composition, with potential contribution for the antioxidant and anti-hypertensive effects.

## 1. Introduction

Salinity is a worldwide problem that affects a large percentage of the earth’s surface, in which salts gradually accumulate in the soil, eventually transforming fertile soil to barren soil [1]. In this process, water-soluble salts are deposited in the soil to an extent that impacts crop productivity, agricultural economics, and the ecosystem [2]. Currently, salt-affected soils cover over 15 percent of the world’s cultivated land [3]. Despite this critical factor that imposes severe constraints on the ecosystem, there is a type of plant able of growing in these salt-affected areas; they are called salt-tolerating plants or halophyte plants [4].

Halophyte plants can grow in extreme conditions of drought, temperature, and salinity (NaCl concentrations above 200 mM) since they respond to salt stress at three levels, e.g., cellular, tissue, and whole plant level [5]. In this context, halophyte plants are a potential solution for degraded soil, freshwater scarcity, and coastal area utilization [6]. Therefore, these plants have been considered alternative crops (biosaline and soilless agriculture) for sustainable use of natural resources.

In Portuguese territory, the halophytes grow along the country’s coast salt marshes, and some companies are already producing some edible species in salt-affected areas using a soilless cultivation system (SCS) to improve their productivity and marketing, assuring their safety and quality. In this cultivation system, nutrients and salt are added to the halophytes through the cultivation media, and these plants are irrigated by flooding, simulating tides, under controlled temperature, and humidity [7]. Regarding food safety, salt marshes ecosystems are natural deposits of heavy metals near a polluted area. As the metals spread along with the tides and periodic floods, they interact with soil and the biotic community [8]. In this way, the halophytes may accumulate large amounts of these contaminants in their aerial and belowground organs, leading to potential impacts on human health and safety [9].

Portugal has become an important producer of edible halophytes. So far there are few studies carried out with cultivated halophytes using a SCS that report their nutraceutical value, as well as their benefits on human health when consumed in equilibrated diets. Therefore, studies involving halophyte species are crucial. In a previous study, the halophyte (*Sarcocornia fruticosa* L.) cultivated using a SCS showed higher nutrient contents (ash, proteins, and fat contents), antioxidant activity, and total phenolic compound content than wild plants [7].

Halophytes are used traditionally as herbs and vegetables and some species are appreciated in gourmet cuisine as a promising substitute for table salt because of their characteristic salty taste. These plants are attracting attention for being sources of phenolic acids, flavonoids, coumarins, vitamins, and carotenoids, which are reported to be health promoters due to their antioxidant properties [10,11,12]. These natural antioxidants are produced by halophytes to maintain their cellular functioning under oxidative stress conditions (e.g., high ionic content, waterlogging, and submersion) [13,14]. Studies show that the regular consumption of phenolic acids and flavonoids exerts cardiovascular protective effects and may reduce the onset or progression of cardiovascular diseases, particularly hypertension [15,16].

In this context, some studies have demonstrated that extracts from halophyte species could be used for the prevention and treatment of heart disease and hypertension (angiotensin-converting enzyme (ACE)-inhibitory activity) due to the presence of polyphenols and bioactive compounds [17,18,19]. For instance, *Mesembryanthemum crystallinum* extract displayed potent ACE-inhibitory activity (90.5% at a concentration of 1 mg/mL), presumably due to the polyphenols identified (three flavonoids (apigenin, diosmin, and luteolin), two phenolic acids (p-coumaric and 4-hydroxybenzoic acids), and a hydroxycinnamic acid derivative (2-O-(p-cumaroyl)-l-malic acid)) [12]. A similar response in terms of the antihypertensive effect was obtained with *Salicornia ramosissima* extract, which can also be attributed to its composition in bioactive compounds, mainly p-coumaric, flavonoids (quercetin and apigenin glycosides), and hydroxycinnamic acids (chlorogenic, ferulic, and dicaffeoylquinic acids) [11]. In the control of blood pressure, the enzyme ACE (a dipeptidyl carboxypeptidase) catalyzes the formation of vasoconstrictor Angiotensin II from Angiotensin I and promotes the degradation of vasodilator bradykinin (BK) [20,21]. Therefore, ACE inhibition has become an interesting target control for hypertension, a common progressive disorder leading to several chronic diseases such as cardiovascular disease, stroke, and renal disease [20,22].

In addition, halophyte plants have been recognized as promising natural ingredients [11]. For this purpose, the present study aimed to study different species of halophytes cultivated using a SCS in Portugal (*Disphyma crassifolium* L., *Crithmum maritimum* L., *Inula crithmoides* L., *Mesembryanthemum crystallinum* L., *Mesembryanthemum nodiflorum* L., *Salicornia ramosissima* J. Woods, and *Sarcocornia fruticosa* (Mill.) A. J. Scott.). Nutritional content, phenolic characterization, and in vitro antioxidant and antihypertensive activities were determined. For the first time, the volatile profile of these plants was described.

## 2. Materials and Methods

### 2.1. Halophyte Plants

The halophyte plants, mostly consumed in gourmet cuisine, were obtained from RiaFresh^®^ (Faro, Portugal), following a soilless cultivation system (SCS). The plants (*Disphyma crassifolium* L., *Crithmum maritimum* L., *Inula crithmoides* L., *Mesembryanthemum crystallinum* L., *Mesembryanthemum nodiflorum* L., *Salicornia ramosissima* J. Woods, and *Sarcocornia fruticosa* (Mill.) A. J. Scott.) were collected between April 2019 and January 2020. The halophyte plants were collected and sent to the laboratory (iBET, Oeiras, Portugal) in a refrigerated transport system (7 °C). Upon reception, the plants were stored in plastic bags at 7 °C for further nutritional, volatile, and phytochemical analyses. The analyses of volatile compounds and phytochemical extraction were performed within 48 h, and nutritional analyses were carried out within 4 days.

### 2.2. Reagents

Methanol (MeOH, 99.8% LC–MS) and acetonitrile (CH_3_CN, 99.9% LC–MS) were purchased from Fisher Scientific (Thermo Fisher Scientific, Waltham, MA, EUA). The ultra-pure water (18.2 MO.cm) was obtained from a Millipore-Direct Q3 UV system (Millipore, Billerica, MA, USA). Formic acid (HCOOH, 98% p.a.) was purchased from Merck (Darmstadt, Germany). Folin–Ciocalteu’s reagent, fluorescein sodium salt, AAPH (2,2-azobis(2-methylpropionamidine)dihydrochloride) and Trolox (6-hydroxy-2,5,7,8-tetramethyl chromane-2-carboxylic acid) were obtained from Sigma-Aldrich (St. Louis, MO, USA). Standards of phenolic compounds, quercetin-3-glucoside (PubChem CID: 25203368) and gallic acid (PubChem CID: 370), were obtained from Sigma-Aldrich (St. Louis, MO, USA), and chlorogenic acid (3-O-caffeoylquinic acid–PubChem CID: 1794427) was purchased from Extrasynthese (Genay, Rhône-Alpes, France).

### 2.3. Nutritional Characterization

#### 2.3.1. Nutritional Parameters

Total fat was determined using the Soxhlet extraction method, total protein was quantified by the Kjeldahl method (F = 6.25), and moisture was determined through oven-drying at 105 ± 1 °C, according to the Association of Official Analytical Chemists (AOAC) [23]. Ash content was determined using sample incineration in a muffle furnace (600 ± 1 °C), and total dietary fibre was quantified as described by the official method of the AOAC (nº.985.29) [23]. The total energy value was calculated according to the European Regulation 1169/2011 (2006/962/EC-European Parliament and Council of the European Union). The fatty acids profile was performed using the fatty acids methyl esters (FAMEs) of the sample analyzed by gas chromatography (GC Agilent 7820, Wilmington, DE, USA) with a flame ionization detector (FID), capillary column Sulpeco SP-2380 (60 m × 0.25 mm; 0.20 mm). The oven temperature was 75 °C for 5 min, 75 to 250 °C (5 °C/min), and 250 °C for 3 min; detector temperature: 280 °C; injector temperature: 250 °C; carrier gas: hydrogen, split ratio: 1:50; and injection volume: 0.6 μL, as described by the AOAC method (nº Ce 2–66) [23]. The FAMEs were identified by comparison of the retention times with FAMEs standard mixture under the same conditions (FAME Mix C4-C24, Sulpeco, Bellefonte, PA, USA) and quantified using area normalization. Carbohydrates were calculated by Equation (1). The analyses were performed in triplicates for each plant.

Carbohydrates = 100 − (moisture + ash + total fat + total protein)(1)

#### 2.3.2. Mineral Composition

The mineral composition of the halophyte plants was determined using Flame Atomic Absorption Spectrometry (FAAS) [23]. Toxic metals were extracted using the digestion process and quantified using total X-ray fluorescence spectroscopy (TXRF, S2 PICOFOX™ spectrometer, Bruker Nano GmbH) element analysis [24]. Briefly, the digestion was performed in Teflon reactors: 200 mg of the sample was weighed into digestion reactors, and 0.3 mL HClO_4_ and 1.7 mL HNO_3_ were added. The digestion occurred for 3 h in an oven at 110 °C in tightly closed reactors [25]. After cooling, 496 µL were recovered into 1.5 mL tubes, and 2 µL of Ga (final concentration 1 mg L^−1^) was added as the internal standard for mineral quantification, spiked with 2 µL Cd (final concentration 1 mg L^−1^) for each sample [26]. Samples were stored at 4 °C until analysis. The analyses were carried out in triplicates for each plant. The TXRF spectra and data evaluation interpretation were accomplished using the Spectra 7.8.2.0 software.

### 2.4. Volatile Composition by Gas Chromatography

A solid-phase microextraction (SPME) followed by the gas chromatography-mass spectrometry (GC-MS) method was carried out to determine the volatile compounds in halophyte plants [11]. Briefly, the fresh plants were crushed with a mortar and pestle until a paste was formed; after 1.5 g of paste was transferred to a 20 mL headspace vial (La-Pha-Pack^®^, Langerwehe, Germany), it was capped with a white PTFE silicone septum (Specanalitica, Carcavelos, Portugal GC-MS vial). The SPME conditions were as follows: extraction temperature was at 40 °C for 40 min, agitation of 10 s, rotating speed of 250 rpm, and desorption time of 3 min at 250 °C. For headspace SPME sampling, a divinylbenzene/carboxen/polydimethylsiloxane (DVB/Car/PDMS) fibre was used (Supelco Analytical, Bellefonte, PA, USA). Analyses were performed on a GCMS QP2010 Plus (Shimadzu^®^, Kyoto, Japan) equipped with an AOC-5000 autosampler (Shimadzu^®^, Kyoto, Japan). The volatile compounds were separated using a capillary column Sapiens (5-MS, Teknokroma, Barcelona, Spain) with dimensions (30 m × 0.25 mm (IS); 0.25 μm (film thickness). The GC-MS conditions were as follows: the injector and detector temperature were maintained at 250 °C; the injection mode was accomplished in the splitless mode for 1.5 min; and high-purity helium (≥99.999%) was used as the carrier gas. The column oven temperature was maintained at an initial temperature of 40 °C for 5 min, increased to 170 °C at a rate of 5 °C min^−1^, and then increased to 230 °C at 30 °C min^−1^ and kept for 4 min. The carrier gas (He) was maintained with a flow of 2.00 mL min^−1^, and the MS interface temperature was kept at 250 °C, as was the ion source temperature. Mass spectra were acquired in the electron ionization (EI) mode at 70 eV in an *m*/*z* range between 29 and 300 with a scan speed of 588 scans s^−1^. The Linear Retention Index (RI) and mass spectra library (NIST 2005 mass spectra database, Boulder, CO, USA) [27,28] were used to perform the putative identification of compounds. The volatile profile of samples was analyzed in duplicates.

### 2.5. Analysis of Phenolic Composition

#### 2.5.1. Extraction of Phenolic Compounds

An ultrasound extraction procedure (USE) was performed for the extraction of phenolic compounds from plants [11]. Briefly, the halophyte plants were crushed with a mortar and pestle after adding liquid nitrogen. To 10 g of the fresh plant, 100 mL of the extraction solvent, consisting of an ethanol:water (80:20, *v*/*v*) solution, was added. Then, the samples were placed in the vortex for 10 s and immediately transferred to an ultrasonic water bath (ArgoLab DU-100, Carpi, Modena, Italy) using the following conditions: 40 kHz, 220 W for 60 min at 25 ± 3 °C. Afterward, the samples were centrifuged at 6000× *g* for 15 min (Sorvall ST16 centrifuge, Thermo Scientific, Osterode, Germany), and the supernatant was collected. The supernatant was evaporated to almost dryness at 40 ± 1 °C under reduced pressure (120 Bar) using a rotavapor (Büchi R-114, Flawil, Switzerland). The residue was dissolved in 2 mL of ethanol:water (50:50, *v*/*v*) solution and filtered through a 0.22 mm SFCA membrane (Branchia, Barcelona, Spain), and then the samples were stored at −18 °C until analysis. The extractions were performed in triplicates for each plant.

#### 2.5.2. HPLC-DAD-ESI-MS/MS

Halophyte extracts were analyzed in a high-performance liquid chromatography (HPLC) system (Waters Alliance 2695, Milford, MA, USA), coupled to a diode array (DAD) detector (Detector Waters 2996, Milford, MA, USA), and outfitted with electrospray ionization source (ESI) and a triple quadrupole mass spectrometer (MS/MS) (Micromass, Waters) [11]. The HPLC system contained an autosampler, solvent degasser, quaternary pump, a pre-column (100RP-18, 5 mm), and a reversed-phase C18 column (LiCrospher 100 RP-18, 250 × 4 mm; 5 mm). Additionally, a thermostatic oven at 35 °C was used for the compounds’ separation. The mobile phase consisted of water-formic acid (99.5%:0.5%) as eluent A and acetonitrile-formic acid (99.5%:0.5%) as eluent B at a flow rate of 0.30 mL/min. All solvents were filtered through a 0.22 mm PVDF membrane (Millipore, Billerica, MA, USA) before analyses. The gradient elution program was as follows: 0–10 min from 99 to 95% A; 10–30 min from 95 to 82% A; 30–44 min from 82 to 64% A; 44–64 min at 64% A; 64–90 min from 64 to 10% A; 90–100 min at 10% A; 100–101 min from 10 to 95% A; 101–120 min at 95% A; and finally returning to the initial conditions. The auto sampler’s temperature was set at 7 °C, and the injection volume was 20 µL. DAD was used to scan the wavelength absorption from 200 to 650 nm. Tandem mass spectrometry (MS/MS) detection was carried out using an electrospray ionization source (ESI) at 120 °C and applying a capillary voltage of 2.5 kV and cone voltage of 30 V. The compounds were ionized in the negative mode and spectra were recorded in the range of *m*/*z* 60–1500. Analytical conditions were optimized to maximize the precursor ion signal ([M−H]^−^). Ultra-high purity argon (Ar) was used as a collision gas. High-purity nitrogen (N_2_) was used both as a drying gas and nebulizing gas. The data acquisition and processing were accomplished using MassLynx software (version 4.1, Waters, Milford, MA, EUA).

#### 2.5.3. HPLC-DAD

The quantification of phenolic compounds was carried out using high-performance liquid chromatography (HPLC) (Vanquish system, Thermo Fisher Scientific, Waltham, MA,USA) equipped with a pump, auto-sampler, and coupled a diode array (DAD) detector [11]. An RP-18 pre-column was also used and thermostated at 35 °C, and the chromatographic separation was performed on a Luna C18 reversed phase (Luna 5 µm C18 (2) 100 Å, 250 × 4 mm; Phenomenex) column. DAD detector was programmed to scan wavelength absorption from 200 to 798 nm at a speed of 1 Hz with a bandwidth of 5 nm. The detection of compounds was monitored using the individual channels (280, 320, and 360 nm) at a speed of 10 Hz with a bandwidth of 11 nm. The autosampler’s temperature was set at 7 °C, and the injection volume was set at 20 µL. The eluents used were eluent A (water-formic acid (99.5%:0.5%)) and eluent B (acetonitrile-formic acid (99.5%:0.5%)) at a flow rate of 0.30 mL/min. The solvents used in chromatography conditions were filtered through a 0.22 µm PVDF membrane (Millipore, Billerica, MA, USA) prior to analysis. The following gradient program was used: 0–10 min from 99 to 95% A; 10–30 min from 95 to 82% A; 30–44 min from 82 to 64% A; 44–64 min at 64% A; 64–90 min from 64 to 10% A; 90–100 min at 10% A; 100–101 min from 10 to 95% A; 101–120 min at 95% A; and finally returning to the initial conditions. Calibration curves (range from 0.78–100 ppm) for chlorogenic acid, gallic acid, and quercetin-3-hexoside were used for the quantification of the phenolic compounds. The DAD data acquisition was performed using the software Chromeleon version 7.0 (Waltham, MA, USA).

### 2.6. Total Phenolic Content

The extracts were quantified in terms of total phenolic content (TPC) according to Folin–Ciocalteu’s colorimetric method [29,30]. Summarily, 230 µL of milli-Q water, 10 µL of the sample, and 15 µL (0.25 N) of Folin–Ciocalteu’s reagent were added in the microplate and mixed at room temperature for 3 min. Afterward, 45 µL of sodium carbonate solution (solution 35%) was added, and the microplate was left to rest in the dark, protected from sunlight, at room temperature for 1 h. The absorbance of the samples was measured at 765 nm on a microplate spectrophotometer (Epoch2 Biotek, Winooski, VT, USA) with the Gen5 3.02 data analysis software spectrophotometer. The standard, gallic acid (1000 mg/L), was used for the calibration curve. The results were expressed as gallic acid equivalents per gram of fresh plant (mg GAE/g). The samples were analyzed in triplicates.

### 2.7. Antioxidant Activity

#### 2.7.1. Oxygen Radical Absorbance Capacity (ORAC) Assay

The ORAC assay was performed using a microplate fluorescent reader (FL800 Bio-Tek Instruments, Winooski, VT, USA) according to Serra et al. [31,32]. This method evaluates the antioxidant capacity of the antioxidant species present in the samples to inhibit the oxidation of fluorescein (3 × 10^−4^ mM) catalyzed by peroxyl radicals generated from AAPH. All samples were analyzed in triplicates. Trolox was used as a reference standard, and the results were expressed as Trolox equivalent antioxidant capacity per gram of fresh plant (µmol TEAC/g), Equation (2). Conc.: concentration.

(2) $$\mathrm{Conc}.\;(\mu \mathrm{mol}\;\mathrm{TEAC}/g\;\mathrm{fresh}\;\mathrm{plant})=\frac{Conc.\mu mol/L\;TEAC}{Conc.extract\;plant\;g/L}$$

#### 2.7.2. Hydroxyl Radical Scavenging Capacity (HOSC) Assay

The HOSC assay was carried out based on the method of Moore et al. [33], using the microplate fluorescence reader (FL800 Bio-Tek Instruments, Winooski, VT, USA). This assay measured the hydroxyl radical scavenging capacity of a sample using fluorescein (9.96 × 10^−8^ M) as a probe and a classic Fenton reaction with FeCl_3_ (3.42 mM) and H_2_O_2_ (0.20 M) as a source of hydroxyl radicals. Samples were analyzed in triplicates, and results were expressed as Trolox equivalent antioxidant capacity per gram of the fresh plant (μmol TEAC/g), see Equation (2).

### 2.8. Antihypertensive Activity Assay

The antihypertensive activity of the halophyte plants’ extracts was evaluated using an angiotensin-converting enzyme (ACE) activity assay kit (Sigma-Aldrich, Saint Louis, MO, USA) [11]. This kit provides a direct and sensitive procedure for measuring ACE levels with the goal of screening for ACE inhibitors on samples, as it is based on the cleavage of a synthetic fluorogenic peptide. Briefly, 10 µL of extract and 40 µL of ACE were added to a 96-well black microplate and incubated at 37 °C for 5 min. The incubation allows contact between the enzyme and the inhibitor. Afterward, 50 µL of the substrate (ACE fluorogenic) was added, and the fluorescence was read every minute for 5 min. A standard curve (0.1 to 0.8 nmol) was used for the quantification of the fluorescent product formed, and the percentage of inhibition was calculated according to the manufacturer’s protocol (CS0002, Sigma-Aldrich). A positive control, Lisinopril (Sigma-Aldrich, Darmstadt, Germany), was used during the assay. The IC_50_ values that correspond to the needed amount of extract to inhibit 50% of ACE, were calculated. The range of extracts’ concentrations tested was between 31.25 and 500 mg/mL.

### 2.9. Statistical Analysis

Descriptive statistical analysis, analysis of variance (ANOVA), and Tukey’s test for multiple data comparisons at a significance level of 5% were performed using GraphPad Prism 9.4 software (GraphPad Software, Inc., La Jolla, CA, USA). The pairwise correlation between the analyzed parameters was established based on Pearson’s correlation coefficient test, at a significance level of 5%. The IC_50_ values were determined using non-linear regression (dose-response inhibition) for the antihypertensive assay.

Multivariate analysis by partial least squares-discriminant analysis (PLS-DA) was performed in the Unscrambler X version 10.4, by CAMO software (Oslo, Norway) as a supervised classification technique that maximizes the differences between the samples and allows the correlation between the predictors, quantified parameters, and the response groups established based on the corresponding halophyte species. For data interpretation, the threshold of |0.5| was used to select the most relevant parameters associated with the sample discrimination [34].

## 3. Results and Discussion

### 3.1. Nutritional Characterization

Halophyte plant species are a category of marine vegetables and have a nutritional composition suitable for human consumption due to their content of micro and macronutrients, such as minerals, proteins, fibre, and polyunsaturated fatty acids. The nutritional content of the different studied halophyte species is shown in Table 1.

The increase of water content (succulence) in the tissues of halophytes is an adaptive strategy when exposed to salinity stress and extreme conditions [7,35]. These plants need to regulate their cellular Na^+^, Cl^−^, and K^+^ concentrations as they adjust to the external water potential and in the accumulated solutes [36]. The content of salts in vegetative organs of halophytes increases with ageing and might reach toxic levels; therefore, these plants tend to lower such unfavourable salt concentrations by increasing the water content in their tissues (succulence) [37]. According to Table 1, *D. crassifolium* and *M. crystallinum* displayed significantly higher moisture content compared to other halophyte species: 96.20 g/100 g FW and 96.30 g/100 g FW, respectively.

The increase of succulence (water content per unit area of the leaf) observed for halophytes makes them more attractive to consumers [7,35]. In this context, the moisture values reported in this study were higher than the ones reported for wild halophytes growing in Portugal, such as *S. ramosissima* (88.20 g/100 g FW), *M. nodiflorum* (91.40 g/100 g FW), and *Sarcocornia perennis* (84.00 g/100 g FW) [7,10,11,38]. The results obtained in this study are according to Castañeda-Loaiza et al. (2020) [7], who reported that cultivated halophytes (*S. maritima* and *S. fruticosa*) showed higher values of moisture content than wild plants. This can be related to the water availability in SCS, since these plants are irrigated twice a day, in a process simulating diurnal tidal floods, contributing to the higher moisture content of cultivated plants [7].

Halophytes have a higher ash content than other edible plants due to their ability to retain minerals, which is highly correlated with the saline environment in which they grow and their mechanisms of tolerance to salt stress [39,40,41]. According to Table 1, halophyte species contain a higher ash content and show a higher NaCl content. Among the halophyte species, *S. ramosissima* (1.50 g/100 g FW; 2.39 g/100 g FW) and *S. fruticosa* (2.80 g/100 g FW; 4.84 g/100 g FW) displayed higher NaCl and chloride concentrations, respectively. *Salicornia* sp. and *Sarcocornia* sp. are highly appreciated in gastronomy due to their salty taste, being recognized as a promising natural ingredient and salt substitute. The significantly greater value of ashes in *S. fruticosa* (5.70 g/100 g FW) is justified by the significantly higher detected salt content, but also other salts that may be present due to the mineral composition. The opposite is also visible since *M. crystallinum* contains a significantly lower ash content (1.39 g/100 g FW) and also presents the lowest salt content.

Total ashes, proteins, and salt contents were significantly higher in *S. fruticosa* and lower in *M. crystallinum,* while *C. maritimum* displayed higher total fat, TDF (total dietary fibre), and energy. A higher concentration of proteins was found in *S. fruticosa* (4.44 g/100 g FW)*, C. maritimum* (3.98 g/100 g FW), and *I. crithmoides* (3.13 g/100 g FW)*,* while in *D. crassifolium* (1.28 g/100 g FW) and *M. crystallinum* (1.27 g/100 g FW), contents were significantly lower. Plant proteins contribute to the down-regulation of insulin and the up-regulation of glucagon, and their intake has been shown to provide protective effects against cardiovascular diseases and cancer [42].

Halophytes have been described as a good source of dietary fibre [7,10,43]. Dietary fibre provides many health benefits, reducing the risk of developing diabetes, obesity, hypertension, and others [44]. However, according to European Regulation (EC) N° 1924/2006 (European Parliament & Council of the European Union, 2006) [45], the claim “source of dietary fibre” may be used for these halophytes when their content in total dietary fibre (TDF) is higher than 3 g/100 g FW. According to this claim, *C. maritimum* (4.40 g/100 g FW) can be considered a source of dietary fibre.

Table 2 shows the fatty acids profile of the halophytes; these plants are sources of essential fatty acids [46,47,48,49,50,51]. Results indicate that polyunsaturated fatty acids (PUFA) are predominant, making up more than 50% of the total fatty acids in all plants, ranging from 52.80% in *C. maritimum* to 63.10% in *S. ramosissima*. Palmitic, linoleic, and linolenic acids were the main fatty acids present in halophyte plants. Linoleic and linolenic acids present several physiological functions, such as intervention in blood coagulation and in inflammatory and immunological responses [52]. *C. maritimum* (28.7%) and *I. crithmoides* (30.5%) contain a higher content of linoleic acid, while *M. crystallinum, S. ramosissima,* and *M. nodiflorum* display a higher content of linolenic acid. Regarding linolenic acid, α-linolenic acid displayed values between 23.5% and 48%: (*M. crystallinum* > *S. ramosissima* > *M. nodiflorum* > *D. crassifolium* > *S. fruticosa* > *I. crithmoides* > *C. maritimum*). Linoleic acid displayed a value between 11.3% and 30.5%: (*I. crithmoides* > *C. maritimum* > *S. fruticosa* > *D. crassifolium* > *S. ramosissima* > *M. crystallinum* > *M. nodiflorum*). The amount of saturated fatty acid (SFA) varied from 27.90% in *M. nodiflorum* to 36.30% in *S. fruticosa*.

The mineral composition of halophyte plants is summarized in Table 3. Storage and distribution of minerals in halophytes are strongly influenced by environmental conditions (e.g., water and soil characteristics, pollutants, and climate) and can also be affected by some characteristics such as the plant maturation stage and part of the plant under study [5,41,53]. As previously described, *S. fruticosa* is the halophyte with the highest NaCl content and, consequently, has the highest sodium content (1120 mg/100 g FW) compared to other species. On the other quantified minerals, *I. crithmoides* and *S. fruticosa* showed the highest content in calcium and potassium, while *S. ramosissima* contained the highest content in magnesium, copper, and manganese.

Previous studies support the potential use of halophytes as natural salt substitutes due to their mineral composition, mainly as sources of potassium and magnesium, which contribute to vascular protection [54,55]. Hypertension and cardiovascular diseases in societies are associated with a high sodium content, as well as a low potassium and magnesium in human diets [56,57]. The interdependency of sodium and potassium in the pathogenesis of hypertension indicates that sodium restriction and increased potassium intake are important strategies for the primary prevention and treatment of hypertension and its cardiovascular consequences [58]. Furthermore, the risk factors for hypertension include, but are not limited to, age, race, family history, obesity, physical inactivity, and tobacco use [59]. In general, the nutritional parameters of halophyte species determined in this study are in accordance with data already reported in the literature [7,11,38,43,60,61].

### 3.2. Potentially Toxic Elements

Estuarine environments are extremely affected by anthropogenic-driven contamination, namely, potentially toxic metallic elements metal [53,62]. However, the use of the soilless cultivation system (SCS) allows for preventing the presence of contamination by toxic metals. Thus, the halophytes displayed lead and cadmium levels (values converted from dried weight to fresh weight according to the moisture content of each species) below 0.3 and 0.2 mg/kg FW, which is notably the maximum content allowed by Regulation (EU) number 1881/2006 for leaf vegetables [63]. Equally, arsenic levels (values were converted from dried weight to fresh weight according to the moisture content of each species) in plants are lower than the maximum levels allowed in certain foods by Commission Regulation (EU) number 2015/1006 (0.2 mg/kg FW) [64]. Hg was below the detection limit of the method of analysis in the plants analyzed (Table 4).

### 3.3. Phytochemical Characterization and Bioactivity

As shown in Table 5, from all the halophyte plants, *S. ramosissima* and *S. fruticosa* stood out for their highest total phenolic content (TPC), 0.41 mg GAE/g FW and 0.33 mg GAE/g FW, respectively. Nevertheless, the value obtained in the studied *S. ramosissima* plants was clearly below the described value for the same species in previous works with plants cropped in 2019 (1.02 ± 0.04 mg GAE/g FW) [11]. The difference could be attributed to the impact of the cultivation system and environmental conditions on the plants’ phenolic composition. Regarding the *S. fruticosa* plant, the TPC value of 0.33 mg GAE/g FW (2.47 mg GAE/g DW), was slightly lower than the values described for the *Sarcocornia* plants collected in the southwestern Iberian Peninsula (3.231–3.892 mg GAE/g DW) [65]. By opposition, *M. nodiflorum*, *M. crystallinum*, and *D. crassifolium* were the species with the lowest TPC values (0.098 to 0.11 mg GAE/g FW). For *M. nodiflorum* and *M. crystallinum*, the obtained TPC results (1.533 and 2.871 mg GAE/g DW, respectively) were higher than the ones described for the same species collected in south Tunisia (1.72 mg/g DW and 1.43 mg/g DW, respectively) [66]. Factors such as the variability in the halophyte species, the cultivation conditions, the cropping year, and the extraction conditions contributed to impairing the comparison of the results obtained and the ones described in the literature.

Following the same trend of the total phenolic content, the in vitro antioxidant activity, measured by ORAC and HOSC assays, expressed by fresh matter, reaches the highest values in *S. ramosissima* and *S. fruticosa*. However, the lowest values in *M. nodiflorum*, *M. crystallinum*, and *D. crassifolium*, suggesting the relevance of the halophytes’ total phenolic content for the peroxyl and hydroxyl radicals’ inhibition (R^2^ = 0.829 and R^2^ = 0.937; Figure S1—Supplementary Materials) and consequent prevention of the oxidative damage in cellular biomolecules.

Regarding the ACE-inhibitory activity, among the halophyte species evaluated, *M. nodiflorum*, *M. crystallinum*, *I. crithmoides*, *S. fruticosa,* and *S. ramosissima* showed the highest antihypertensive activity, requiring a lower extract concentration to inhibit an angiotensin-converting enzyme (ACE), and *C. maritimum* showed the lowest antihypertensive activity, showing a higher extract concentration to inhibit ACE. The weak correlation between the total phenolic content and the antihypertensive activity (R^2^ = 0.0186) indicated that in the phenolic extracts obtained from the halophytes’ specific phenolic compounds had a low impact on this inhibition [67]. However, the presence of other components in phenolic extracts, such as the ACE-inhibitory peptides (low molecular weight peptides), could contribute to the plant’s antihypertensive activity [68,69].

#### 3.3.1. Composition in Individual Phenolic Compounds

In the halophytes, the tentatively identified phenolic compounds were classified into different classes of phenolic acids (hydroxycinnamic and hydroxybenzoic acids), flavonoids (flavones, flavanals, flavonols, and flavanones), and hydrolysable tannins (gallotannins). These compounds exert potential antioxidant, anti-inflammatory, anti-carcinogenic [70,71], anti-microbial [72], and inhibitory activity in key enzymes associated with, neurodegenerative diseases, e.g., Alzheimer’s disease (e.g., acetylcholinesterase, AChE and butyrylcolinesterase, and BuChE), diabetes *mellitus* type 2 (α-amylase and α-glucosidase), and skin hyperpigmentation/food oxidation (tyrosinase) [73]. The qualitative and quantitative abundance of phenolic compounds in the halophytes is dependent on several factors, such as the environmental growth conditions and the analyzed species. Despite the existent amount of collected information in the plant database for the halophyte and salt-tolerant plants [74], the data regarding their phenolic composition remains limited, which compromises qualitative and quantitative data comparison. The study herein aims to fill the gap of knowledge, describing the phenolic composition of different halophyte species grown under the same soilless cultivation conditions.

**Figure A1:**
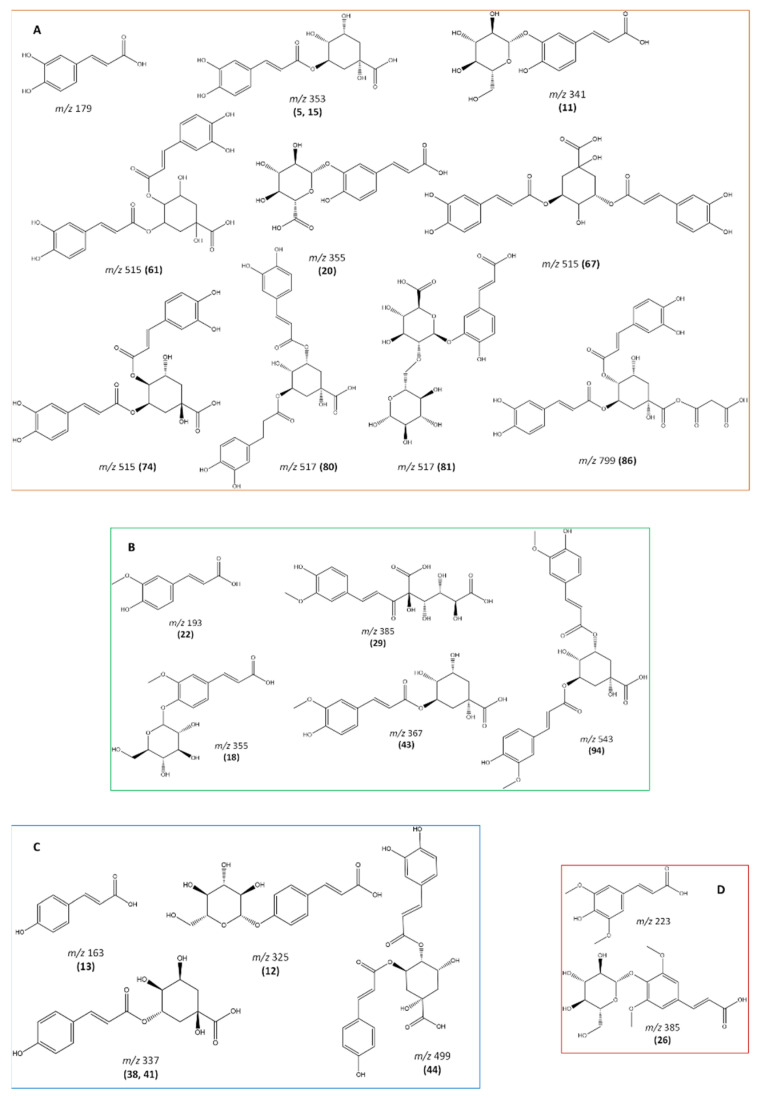
Molecular structure of some compounds, derived from caffeic acid (*m*/*z* 179), (**A**) ferulic acid (*m*/*z* 193, **22**), (**B**) *p*-coumaric acid (*m*/*z* 163, **13**), (**C**) and sinapic acid (*m*/*z* 223), (**D**) tentatively identified in the halophyte plants.

In the halophytes studied herein, 46 of the 94 tentatively identified compounds were hydroxycinnamic acids [75], mostly derivatives of caffeic acid (Figure A1A), ferulic acid (Figure A1B), *p*-coumaric acid (Figure A1C), and sinapic acid (Figure A1D), showing the typical molecular ion [M−H]^−^ at *m*/*z* 179 (compounds **5**, **8**, **11**, **15**, **20**, **21**, **30**, **32**, **61**, **67**, **74**, **80**, **81**, **84**, and **86**), *m*/*z* 193 (compounds **18**, **19**, **22**, **24**, **29**, **40**, **43**, **89**, **90**, and **94**), *m*/*z* 163 (compounds **1**, **12**, **13**, **23**, **34**, **38**, **39**, **41**, **44**, **47**, **54**, **56**, **62**, **66**, **71**, **75**, **77**, **78**, **88**, and **93**), and *m*/*z* 223 (compound **26**) in the corresponding mass fragmentation spectra, Table A1—Appendix A. The presence of these hydroxycinnamic acid derivatives was previously reported in *S. ramosisssima* [76], in conventional and microwave *S. ramosissima* extracts [77], as well as in other halophyte plants such as *Glaux maritima* roots [78], and in *C. maritimum* [79], as a survival molecular adaptation to saline soil conditions, as described by Pungin et al. [78].

As shown in Table A1, there was high qualitative diversity in the phenolic composition of the different studied halophyte plants, with some compounds, such as the ferulic acid derivatives being mostly detected in the *S. ramosissima*, *D. crassifolium*, *I. crithmoides*, *M. nodiflorum*, and *M. crystallinum*, but not in the *S. fruticosa* and *C. maritimum* species. Considering the quantified amounts of hydroxycinnamic acids in the different halophyte species, Table 6 and Table S1—Supplementary Materials, *S. ramosissima* stood out as the species with the highest content of hydroxycinnamic acids, namely, caffeic acid derivatives, such as 3,4-, 3,5-, and 4,5-dicaffeoylquinic acids (compounds **61**, **67**, and **74**) and caffeoylhydrocaffeoyl quinic acid (compound **80**). These compounds were also described in *S. europaea* extracts [80], inhibiting the accumulation of cholesteryl ester hydroperoxide (CE-OOH) and therefore the blood plasma oxidation and the atherosclerotic plaques [81]. *C. maritimum* is highlighted from the remaining species as the one with the highest chlorogenic acid content (compound **15**) [82], Table S1—Supplementary Materials. In the same way, *S. fruticosa* had the highest content of chlorogenic acid (compound **15**) content, and a similar amount (13.661 µg/g FW) was reported for *Sarcocornia perennis alpini* (12.240 µg/g FW) [83]. In addition to the high chlorogenic acid content, *C. maritimum* plants were an important source of *p*-coumaroylquinic acid isomeric forms, compounds **38** and **41**, 78.310 µg/g FW and 43.242 µg/g FW, respectively, which is in line with Siracusa et al. [84]. The compound *p*-coumaroylquinic acid was also present with a high content in *S. fruticosa* and *S. ramosissima* plants (11.764 µg/g FW and 3.644 µg/g FW, respectively). Diverse *p*-coumaric acid derivatives were found with considerably high contents among the analyzed halophytes (compounds **12** and **34** in *C. maritimum*; compounds **44** and **23** in *S. fruticosa*; compounds **88** in *D. crassifolium*; and compounds **54** and **56** in *M. crystallinum*). The non-conjugated form of *p*-coumaric acid (compound **13**) was quantified in *I. crithmoides* and *S. ramosissima* plants in a relatively lower content than the *p*-coumaric acid conjugated forms. The presence of *p*-coumaric acid and its derivatives in halophyte plants, such as *M. crystallinum*, have been related to the in vitro and in vivo antioxidant metal-ion scavenging properties of these plants [12]. *M. nodiflorum* was the species with the lowest amount of hydroxycinnamic acids.

**Figure A2:**
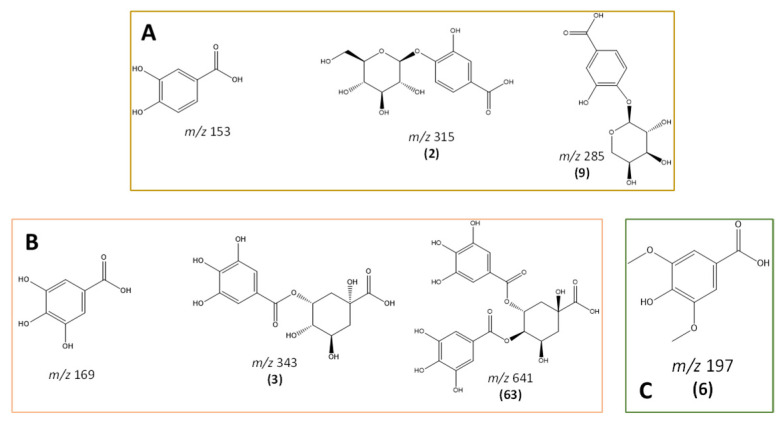
Molecular structure of some compounds, derived from protocatechuic acid (*m*/*z* 153), (**A**) gallic acid (*m*/*z*169), and (**B**) syringic acid (*m*/*z* 197, **6**), (**C**) tentatively identified in the halophyte plants.

Regarding the hydroxybenzoic acids, based on the respective fragmentation mass pattern, in the studied halophyte plants, the main compounds were tentatively identified as derivatives of protocatechuic acid (*m*/*z* 153), gallic acid (*m*/*z* 169), and syringic acid (*m*/*z* 197), Figure A2—Appendix A. As described previously for the hydroxycinnamic acids, in the halophyte species, there was also a huge qualitative diversity in the hydroxybenzoic acids composition. Unlike most halophytes, in the *D. crassifolium* and *M. crystallinum* species, none of the studied hydroxybenzoic acids were detected. The lower content of *D. crassifolium* and *M. crystallinum* species in salt showed a positive association with the absence of hydroxybenzoic acids in these plants. As described by Pungin et al. [78] and Qasim et al. [85], despite the plant’s adaptive variability in salt environmental conditions, the phenolic compounds, particularly the phenolic acids, accumulate in the halophytes as a protective response against the oxidate stress imposed by the soil salinization; therefore, in the plants with lower salt content a lower phenolic content is expected. From a quantitative point of view, *S.ramosissima* showed the highest content of hydroxybenzoic acids, which is particularly thanks to the protocatechuic acid arabinoside (compound **9**) content, Table 6 and Table S1—Supplementary Materials. This compound was also detected in *S. fruticosa* in a lower amount. In *C. maritimum*, the protocatechuic acid esterified to a glycoside moiety was the most abundant hydroxybenzoic acid compound. The quantification of the aglycone form of protocatechuic acid was made previously by Pungin et al. [78] for other halophytes, namely, *Spergularia marina* (L.) and *Glaux maritima*.

**Figure A3:**
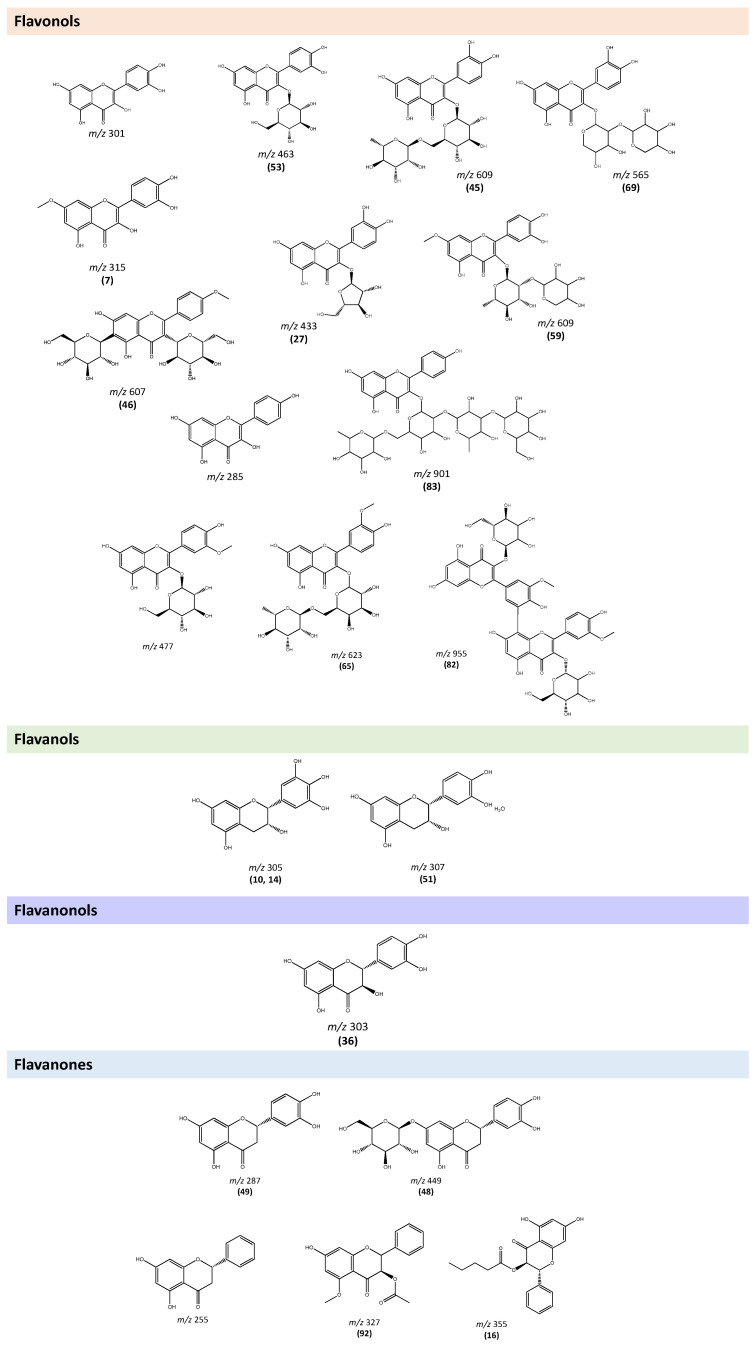
Molecular structure of some flavonoids derived from the flavonols quercetin, *m*/*z* 301; kaempferol, *m*/*z* 285; isorhamnetin-glucoside, *m*/*z* 477 and flavanone pinocembrin, *m*/*z* 255, identified in the halophyte species. The tentative identification of the remaining compound is detailed in Table A1.

**Figure A4:**
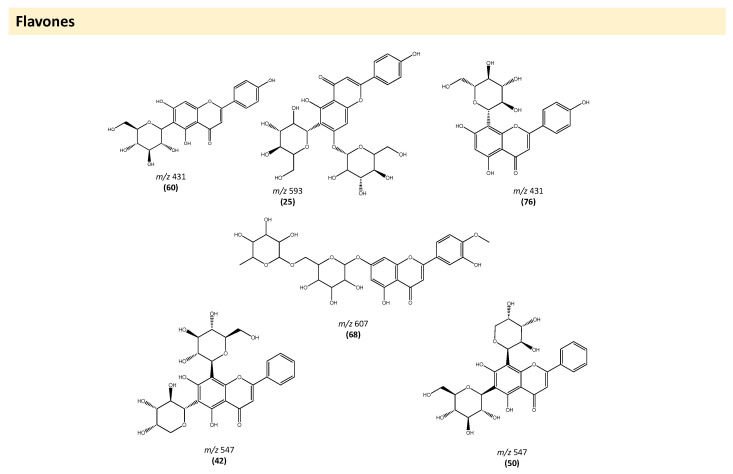
Molecular structure of some flavonoids derived from the flavones, apigenin 6-C-glucoside, *m*/*z* 431; apigenin 6-C-glucoside-7-O-glucoside, *m*/*z* 593; diosmetin-7-O-rutinoside, *m*/*z* 607; and chrysin-6-C-arabinoside-8-C-glucoside, *m*/*z* 547, identified in the halophyte species. The tentative identification of the remaining compound is detailed in Table A1.

In the studied halophyte plants, 35 compounds were classified as flavonoids. The most diverse flavonoids (Figure A3 and Figure A4—Appendix A) belong to the flavones and flavonols classes [86], which included 13 and 10 different compounds, respectively. The flavanol class included five tentatively identified compounds, and the remaining classes of flavanones and flavanonols comprised, respectively, six and one compounds, Table A1.

The flavonols, putatively identified in the halophytes, were derivatives of quercetin, [M−H]^−^ at *m*/*z* 301, kaempferol, [M−H]^−^ at *m*/*z* 285, and isorhamnetin, [M−H]^−^ at *m*/*z* 315, esterified with sugar units (e.g., glucoside, rutinose, and robinobioside) in C3 of C ring, but also methylated forms of quercetin such as rhamnetin (compound **7**). The kaempferol derivative (compound **83**) was tentatively identified for the first time in these plants, based on the fragmentation pattern, with the presence of the fragment ion *m*/*z* 285 (kaempferol), *m*/*z* 593 (kaempferol rhamnosyl glucosyl fragment), and *m*/*z* 739 (kaempferol 3-glucosyl (1-3) rhamnosyl (1-6) galactoside with -OH group loss) previously identified in *Cammellia sinensis* [87]. This flavonoids’ class was the most abundant in the halophytes, particularly in *S. fruticosa*, at the expense of isorhamnetin 3-O-robinobioside (compound **65**) and rhamnetin hexosyl pentoside (compound **59**), tentatively identified, for the first time in this species, based on the fragmentation pattern with fragment ions at *m*/*z* 315 (rhamnetin), *m*/*z* 477 (rhamnetin hexosyl), and *m*/*z* 609 (rhamnetin hexosyl attached to a pentose residue without -H_2_O group), and in *C. maritimum*, owing to quercetin 3-O-rutinoside and quercetin 3-O-glucoside (compounds **45** and **53**). In *D. crassifolium*, the most abundant flavonols were derivatives of isorhamnetin rutinoside (compound **58**) and isorhamnetin glucoside (compound **73**), and in *M. nodiflorum* the avicularin (compound **27**) was the major flavonol. In *S. ramosissima*, the flavonols, quercetin 3-O-glucoside (compound **53**), and the dimeric form of isorhamnetin glucoside (compound **82**) were the most abundant ones. This last compound was also previously reported by Hanen et al. [66] in *Mesembryanthemum* genus.

In the halophytes, the tentatively identified flavones were derivatives of apigenin, [M−H]^−^ at *m*/*z* 269, chrysin, [M−H]^−^ at *m*/*z* 253, acacetin, [M−H]^−^ at *m*/*z* 283, and luteolin, [M−H]^−^ at *m*/*z* 285, usually in their conjugated form with glucosyl (C_6_H_11_O_6_−), arabinosyl (C_5_H_9_O_5_-), and robinobiosyl (C_12_H_21_O_10_-) radicals. In *D. crassifolium*, luteolin (compound **64**) was the major flavone. In *C. maritimum*, the most abundant flavones were glycosidic forms of apigenin (compounds **25** and **60**), and in *M. crystallinum,* from the different flavones, the chrysin derivative (compound **50**) stood out by its abundance. This last result contradicts the higher relative abundance of apigenin, diosmin, and luteolin described by Calvo et al. [12] for *M. crystallinum* plants collected from the northwest coast of Spain (Galicia). Contrarily to most of the halophyte species, in *S. fruticosa* and *S. ramosissima* no flavones were detected.

The flavanols, derived from catechin isomer, with trans configuration, and epicatechin isomer, [M−H]^−^ at *m*/*z* 289, with cis configuration, were mostly found in their conjugated forms with galloyl, epigalloyl, and water residues. From the different studied halophytes, *S. fruticosa* was characterized by the highest abundance of the epicatechin derivative (compound **55**), and *M. nodiflorum* was characterized by the highest abundance of epicatechin hydrate (compound **51**) and gallocatechin (compound **10**). The presence of epicatechin was previously reported in *S. fruticosa* and *M. nodiflorum* plants [7], as well as in other halophyte species, such as *Spergularia marina* L. and *Glaux maritima* L. [78]. Contrarywise, in *C. maritimum,* no flavanols were detected.

The detected flavanones were derivatives of pinobanksin, [M−H]^−^ at *m*/*z* 271, pinocembrin, [M−H]^−^ at *m*/*z* 255, and eriodyctiol, [M−H]^−^ at *m*/*z* 287, conjugated to ether, ester, or sugar groups. While I. *crithmoides* was particularly rich in the pinobanksin derivative (compound **31**), *M. nodiflorum* showed a higher abundance of eriodyctiol (compound **49**) and its hexoside derivative (compound **48**), which was particularly abundant in *S. fruticosa*. The pinobanksin derivatives (compounds **16**, **31**, and **92**) were identified for the first time in the studied halophyte plants. However, the presence of pinobanksin in halophytes was previously described in the propolis produced from *Zuccagnia punctata*, *Larrea divaricata*, and *Larrea cuneifolia* halophytic shrubs, present in the vegetation of the Monte region in Argentina [88]. The presence of eriodictyol hexoside and pinocembrin was previously reported by Kramberger et al. [89], in the halophyte *Helichrysum italicum*.

In the flavanonol class, only dihydroquercetin, also known as taxifolin (*m*/*z* 303), compound **36**, was detected. *S. fruticosa* species was the only one with quantifiable amounts of this compound. This compound was recently associated with positive effects in nitric oxide production and endothelial dysfunction, contributing to a vasorelaxant effect and cardiovascular protection [90]. Compounds **28**, **57**, and **79**, not classified in the previous classes, were less abundant in the plants and present in *D. crassifolium*, *I. crithmoides,* and *M. nodiflorum,* respectively. Nevertheless, the positive impact of these compounds on human health has been extensively described [91,92,93].

#### 3.3.2. Integrating the Nutritional, Phytochemical, and Bioactivity Parameters 

**Figure 1:**
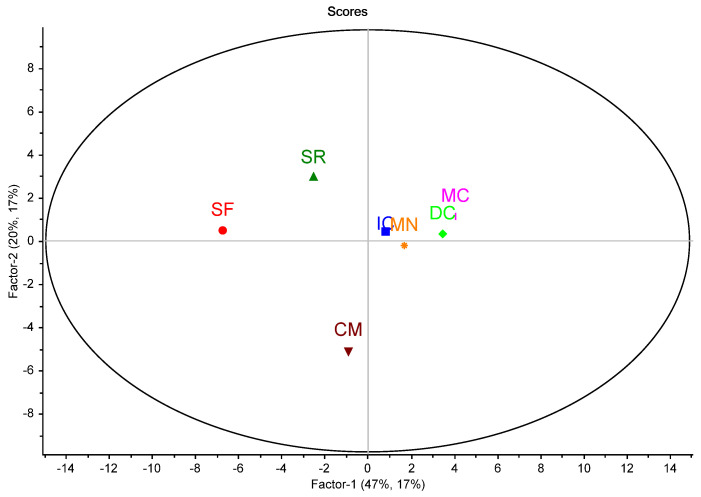
Partial least square-discriminant analysis (PLS-DA) score plot of the halophyte species distribution (IC, *Inula crithmoides*; SF, *Sarcocornia fruticosa*; SR, *Salicornia ramosisssima*; DC, *Disphyma crassifolium*; CM, *Crithmum maritimum;* MN, *Mesembryanthemum nodiflorum*; MC, *Mesembryanthemum crystallinum*) in a space defined by the two first factors. In the model, the average values for the nutritional and phytochemical parameters, determined for the analytical triplicates, were considered as predictor variables (accumulated variance of 67%) and the groups, defined by the halophyte’s specie, as the response variable, with an accumulated variance of 34%.

**Figure 2:**
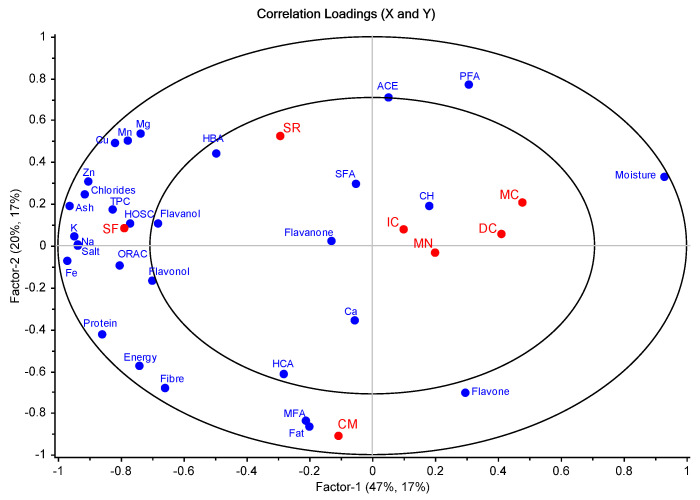
Partial least square-discriminant analysis (PLS-DA) correlation loading plot showing the correlation between the analyzed nutritional (moisture, protein, fat, ash, fibre, carbohydrates (CH), energy, salt, chlorides, Na, Ca, K, Fe, Mg, Mn, Zn, Cu, SFA, MFA, and PFA) and phytochemical parameters (TPC, ORAC, HOSC, inverse values of ACE inhibition, hydroxybenzoic acids (HBA), hydroxycinnamic acids (HCA), flavanol, flavonol, flavones, and flavanone) (in the picture in blue), and the groups based on the halophyte species (IC, *Inula crithmoides*; SF, *Sarcocornia fruticosa*; SR, *Salicornia ramosisssima*; DC, *Disphyma crassifolium*; CM, *Crithmum maritimum;* MN, *Mesembryanthemum nodiflorum*; MC, *Mesembryanthemum crystallinum*) (in red). In the model, the inner circle indicates correlation loadings lower than |0.5| and the space between the inner and the outer circles the correlation loadings higher than |0.5| and lower than |1.0|.

The diversity of the halophyte species, regarding the nutritional, phytochemical (total contents), and bioactivity parameters, can be summarized through multivariate analysis, namely, by partial least square-discriminant analysis (Figure 1 and Figure 2). For data systematization, the phenolic compounds were classified into different families. The ones only quantified in one species were excluded from the multivariate analysis. Concerning the nutritional and the phytochemical composition, as shown in Figure 1, the most different species, occupying an external position on the score plot, were *S. fruticosa, S. ramosissima,* and *M. crystallinum.*

Based on the established correlations between the analyzed parameters and the defined groups (Figure 2), along with the first factor, the position of the *S. fruticosa* in the left-extreme corner of the plot was explained by its lowest moisture content and highest content in protein, fibre, ash, salt, chlorides, minerals (Na, K, Fe, Mg, Mn, Zn, Cu), TPC, flavanol, flavonol, and antioxidant activity parameters (ORAC and HOSC assays). These last in vitro antioxidant parameters, ORAC and HOSC, were highly correlated to the TPC value (Pearson’s R of 0.945 and 0.968, respectively, Table S1—Supplementary Materials). The highest moisture content in plants *M. crystallinum* and *D. crassifolium* conjugated with their lowest protein, energy, fibre, ash, salt, chlorides, Na, K, Fe, Mg, Mn, Zn, Cu, TPC, flavanol, and flavonol contents, as well as to their lowest antioxidant activity (ORAC and HOSC assays), justifies the position of *M. crystallinum* and *D. crassifolium* in the right side of the horizontal first factor axis. On the first quadrant, along the second factor, the upper position of the *S. ramosisssima* plant could be attributed to the highest hydroxybenzoic acids (HBA) content, polyunsaturated fatty acids (PFA) contents, and their high antihypertensive activity (represented as the inverse values of the ACE inhibitor activity). In the diametrically opposite position, along the second factor, *C. maritimum* was distinguished from the remaining samples based on the highest fat and monounsaturated fatty acids (MFA), hydroxycinammic acids (HCA), and flavone contents.

From the set of studied halophyte species, *M. nodiflorum* and *S. fruticosa* are not only different in terms of the total phenolic content (Section 3.3 and Section 3.3.1) but also opposed in their individual phenolic composition, Table S1—Supplementary Materials. The halophytes *S. fruticosa* and *M. nodiflorum* had the most contrasting phenolic profiles. While in *M. nodiflorum*, the hydroxybenzoic acids, compounds **37**, **63**, **85**, and **91**; the hydroxycinnamic acids, compounds **18**, **19**, **39**, **84**, and **94**; the flavonols, compounds **10** and **51**; the flavonols, compounds **27**, **69**, and **72**; the flavones, compounds **42** and **87**; and the flavanones, compounds **33**, **49**, and **92** were the most abundant compounds, in *S. fruticosa* the most relevant compounds were the hydroxybenzoic acid, compound 3; the hydroxycinnamic acids, compounds **1**, **8**, **15**, **23**, **30**, **32**, **44**, **71**, **75**, and **77**; the flavanol, compound **55**; the flavonols, compounds 36 and 65; the flavanone, compound 48; and the flavone, compound **59**. Whilst *I. crithmoides* had a relatively higher abundance of specific hydroxybenzoic acids, C4, C6, and C17; hydroxycinnamic acids, C8, C21, C43, C44, C66, and C81; flavonoids, C7 (flavonol), C31 (flavanone), C52 (flavanol), and C76 (flavone); and monocarboxylic acid, C57, *C. maritimum* was particularly rich in the hydroxybenzoic acid, C2; hydroxycinnamic acids, C11, C12, C15, C34, C38, C41, and C78; flavonols, C45 and C53; and flavones, C60 and C68, Table S1—Supplementary Materials. *S. ramosissima* was characterized by a high abundance of the hydroxybenzoic acid C9; hydroxycinnamic acids C22, C40, C61, C67, C74, and C80; and the flavonol C82. In *D. crassifolium*, the hydroxycinnamic acids C13, C20, C47, C86, C88, C89, C90, and C93, the flavonoids C58, C64 (flavones), C73 (flavonol), and C14 (flavanol), and the coumarin C28 stood out as the most abundant compounds. Finally, in *M. crystallinum*, the compounds with higher abundance were the hydroxycinnamic acids C54, C56, and C62; flavones C46 and C50; flavanone C16; and the flavonols C35, C70, and C83.

Specific phenolic compounds such as the hydroxycinnamic acids C18, C19, C39, C84, and C94; the hydroxybenzoic acids C37, C63, C85, and C91; the flavonoids, C10, C51 (flavanols), C21, C69, C72 (flavonol), C33, C92 (flavanone), and C87 (flavone); and the gallotannin C79, were highly correlated to the in vitro antioxidant activity measured by ORAC and HOSC, showing Pearson’s R values higher than 0.5. Regarding the anti-hypertensive activity of extracts, in this study, the presence of lower amounts of specific hydroxycinnamic acids, C1, C8, C15, C23, C30, C32, C44, C71, C75, and C77; hydroxybenzoic acids, C3; and flavonoids, C36 (flavanonol), C48 (flavanone), C55 (flavanol), C59, and C65 (flavonols), was correlated to higher antihypertensive activity (Pearson’s R was lower than −0.5, Table S1—Supplementary Materials). This result was in agreement to the reduced impact of the compounds classified as flavonoids linked to sugars, and to the phenolic acids, in the anti-hypertensive activity [67].

### 3.4. Volatile Compounds Profile

The volatile profile of halophytes was studied by SPME-GC-MS, and the putative identification of their volatile compounds was carried out by comparing the mass spectra with the mass spectra bank from libraries and the Linear Retention Index (LRI). Compounds were classified by their chemical classes and odor descriptions according to the literature. In order to compare the volatile profile among species, the percentage of peak area was measured in relation to the total area of the chromatogram (Supplementary Materials, Table S2).

**Figure 3:**
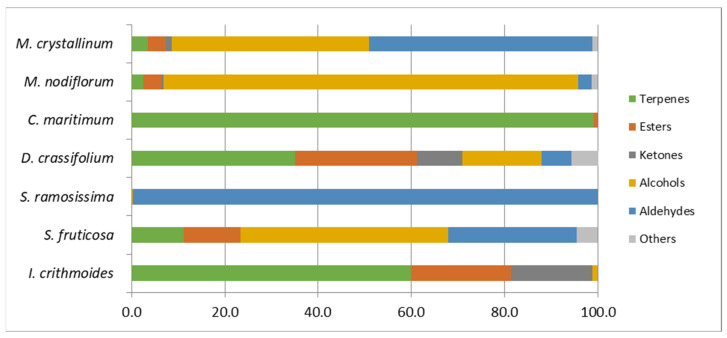
Percentage component bar chart showing the relative abundance of the chemical classes of volatile compounds in the halophyte species.

Results showed that the main chemical classes detected in the volatile profile of these halophyte species were the terpenes (30.36%), alcohols (27.76%), and aldehydes (26.34%), while esters (9.78%) and ketones (4.14%) were minor classes. *C. maritimum* (99.10%; 0.90%), *I. crithmoides* (59.85%; 21.48%), and *D. crassifolium* (35.12%; 26.09%) displayed as most abundant volatiles terpenes and esters, respectively (Figure 3). In contrast, *M. nodiflorum* (88.90%; 2.85%), *S. fruticosa* (44.56%; 27.56%), and *M. crystallinum* (42.44%; 47.88%) showed predominance of alcohols and aldehydes, respectively, while *S. ramosissima* (99.70%) was richer in aldehydes.

The main terpenes identified in *C. maritimum* were the m-mentha-4,8-diene (30.44%), thymol methyl ether (23.59%), alpha-fenchene (17.24%), and p-cymene (13.41%). In *I. crithmoides*, the main terpenes were p-cymene (22.94%), alpha-phellandrene (17.41%), and camphene (8.34%), and in *D. crassifolium,* thymol methyl ether (20.74%), p-cymene (9.03%), and alpha-gurjunene (3.68%) were the most abundant terpenes, as detailed in Supplementary Materials, Table S2. Concerning previous studies, the leaves of *C. maritimum* have been reported to contain thymolmethylether (12.9 to 15.5%) and p-cymene (3.7 to 9.3%) [94,95]. In the aerial part of *I. crithmoides*, the presence of alpha-phellandrene (0.9–26.2%), p-cymene (trace-53.8%), and camphene (5.2%) were also reported [96,97].

In the *S. ramosissima*, the two aldehydes identified as octanal (47.68%) and 2-hexenal (45.65%) were responsible by the typical grassy green aroma of this plant [98]. The major compounds identified, respectively, in *M. crystallinum* and *S. fruticosa* included 1-hexanol (40.33%; 44.04%) and 2-hexenal (36.66%; 27.56%) as being responsible for odors described as herbal and green [99,100]. In addition, 1-hexanol (77.80%) and 3-hexen-1-ol (8.58%) were major volatile compounds in the *M. nodiflorum*. The 3-hexen-1-ol also identified in *I. crithmoides*, *D. crassifolium,* and *C. maritimum* has been described in the literature as having an odor of green, marine, and seaweed [101]. A previous study exhibited that 3-hexen-1-ol was the major volatile compound responsible for the marine odors in *S. ramosissima* [11].

In fact, there was a qualitative diversity of phenolic compounds [102,103,104,105,106,107,108,109,110,111,112,113,114,115,116,117,118,119,120,121,122,123,124,125,126,127,128,129,130,131,132,133,134,135,136,137,138,139,140,141,142,143,144,145,146,147,148,149,150,151,152] identified in halophytes, as shown in Table A1 (Appendix A). While, for the first time, the profile of vol-atile compounds [153,154,155,156,157,158,159,160,161,162,163,164,165,166,167,168,169,170,171,172,173,174,175,176,177,178,179,180,181,182,183,184,185,186,187,188,189,190,191,192,193,194,195,196,197,198,199,200,201,202,203,204,205,206,207,208,209,210,211,212,213,214,215,216,217,218,219,220,221,222,223,224,225,226,227,228,229,230,231,232,233,234,235,236,237,238,239,240] was studied (Table S2—Supplementary Materials). 

## 4. Conclusions

The halophyte species (*I. crithmoides*, *S. fruticosa*, *S. ramosissima*, *D. crassifolium*, *C. maritimum*, *M. nodiflorum*, and *M. crystallinum*) can be considered alternatives to the traditional table salt, with advantages due to their added nutritional and phytochemical values. When used as natural salt alternatives, these plants contribute with a salty taste due to their mineral composition, mainly as sources of sodium, potassium, and magnesium. This study showed that there are significant differences between species. Under the same soilless cultivation conditions, *S. fruticosa* contained a higher content of protein, ash, salt, chlorides, minerals (Na, K, Fe, Mg, Mn, Zn, Cu), and flavonoids. In contrast, *S. ramosissima* presented a higher content of phenolic acids and polyunsaturated fatty acids. Considering the phenolic profile of the plants, *S. fruticosa* and *M. nodiflorum* showed the most distinct profiles, with a relatively higher abundance of hydroxycinnamic acids in *S. fruticosa,* and a relatively higher abundance of flavonoids in *M. nodiflorum* and *S. ramosisssima* showed the highest content of hydroxycinnamic and hydroxybenzoic acids. The most abundant flavonoids class, especially in *S. fruticosa*, was the flavonols, namely, the compound tentatively identified, for the first time in this species, as rhamnetin hexosyl pentoside. The phytochemical diversity of the studied halophytes clearly indicates the advantages of consuming these species in the diet as a strategy to enhance the diet’s nutraceutical value and, consequently, their antioxidant activity and anti-hypertensive effect. Future work is mandatory in order to increment the knowledge about these halophytes and the relationship between their bioactive composition and possible health effects.

## Figures and Tables

**Table 1:** Nutritional parameters of the halophyte species.

*Nutritional Parameters*	*I. crithmoides*	*S. fruticosa*	*S. ramosissima*	*D. crassifolium*	*C. maritimum*	*M. nodiflorum*	*M. crystallinum*
*(g/100 g FW)*							
Moisture	91.8 ± 0.91 ^b^	86.6 ± 0.87 ^d^	91.30 ± 0.91 ^bc^	96.20 ± 0.96 ^a^	88.80 ± 0.89 ^cd^	93.50 ± 0.93 ^b^	96.30 ± 0.96 ^a^
Total ash	2.41 ± 0.09 ^c^	5.70 ± 0.23 ^a^	3.44 ± 0.14 ^b^	1.40 ± 0.06 ^d^	2.18 ± 0.09 ^c^	2.19 ± 0.09 ^c^	1.39 ± 0.05 ^d^
Protein	3.13 ± 0.12 ^c^	4.44 ± 0.17 ^a^	2.65 ± 0.10 ^d^	1.28 ± 0.05 ^e^	3.98 ± 0.15 ^b^	2.41 ± 0.09 ^d^	1.27 ± 0.05 ^e^
Total fat	0.30 ± 0.01 ^c^	0.30 ± 0.01 ^c^	0.20 ± 0.002 ^d^	0.20 ± 0.002 ^d^	0.50 ± 0.005 ^a^	0.40 ± 0.004 ^b^	0.20 ± 0.002 ^d^
Carbohydrates	0.70 ± 0.03 ^a^	0.06 ± 0.002 ^d^	0.31 ± 0.01 ^b^	0.32 ± 0.01 ^b^	0.14 ± 0.05 ^c^	0.20 ± 0.008 ^c^	0.04 ± 0.001 ^d^
Total dietary fibre	1.70 ± 0.05 ^d^	2.90 ± 0.09 ^b^	2.10 ± 0.06 ^c^	0.60 ± 0.02 ^g^	4.40 ± 0.13 ^a^	1.30 ± 0.04 ^e^	0.80 ± 0.02 ^f^
Salt ^1^	1.05 ± 0.14 b^c^	2.80 ± 0.36 ^a^	1.50 ± 0.19 ^b^	0.77 ± 0.10 ^c^	1.42 ± 0.18 ^b^	1.30 ± 0.17 ^bc^	0.98 ± 0.13 ^bc^
Chloride	1.55 ± 0.09 ^c^	4.84 ± 0.29 ^a^	2.39 ± 0.14 ^b^	1.01 ± 0.06 ^d^	1.19 ± 0.07 ^cd^	1.25 ± 0.07 ^cd^	0.98 ± 0.06 ^cd^
Energy (kcal/100 g FW)	21.40 ± 0.86 ^c^	26.30 ± 1.05 ^b^	16.60 ± 0.66 ^d^	8.10 ± 0.32 ^e^	29.20 ± 1.17 ^a^	15.80 ± 0.63 ^d^	8.50 ± 0.34 ^e^

Results are expressed as mean values ± standard deviation (n = 3). ^1^ Calculated: salt (g) = sodium (g) × 2.5. The letters (a–f) denote significant differences between the halophytes using Tukey’s test (*p* < 0.05). For all halophytes the phenotypic stage was green.

**Table 2:** Fatty acids profile (relative percentage, %) in the halophyte species.

*Fatty Acids* *(Relative Percentage,%)*		*I. crithmoides*	*S. fruticosa*	*S. ramosissima*	*D. crassifolium*	*C. maritimum*	*M. nodiflorum*	*M. crystallinum*
Capric acid	C10:0	<0.05 *	<0.05 *	0.10 ± 0.01 ^c^	0.50 ± 0.01 ^b^	0.40 ± 0.01 ^b^	1.80 ± 0.01 ^a^	0.40 ± 0.01 ^b^
Lauric acid	C12:0	<0.05 *	0.20 ± 0.01 ^d^	0.30 ± 0.01 ^cd^	0.70 ± 0.01 ^a^	0.40 ± 0.01 ^bc^	0.20 ± 0.01 ^d^	0.50 ± 0.01 ^b^
Myristic acid	C14:0	0.80 ± 0.01 ^c^	0.50 ± 0.01 ^d^	0.60 ± 0.01 ^d^	1.40 ± 0.01 ^a^	1.00 ± 0.01 ^b^	0.60 ± 0.01 ^d^	0.90 ± 0.01 ^bc^
Myristoleic acid	C14:1	0.10 ± 0.01 ^b^	<0.05 *	<0.05 *	<0.05 *	<0.05 *	<0.05 *	0.40 ± 0.01 ^a^
Pentadecanoic acid	C15:0	0.40 ± 0.01 ^a^	0.10 ± 0.01 ^b^	0.10 ± 0.01 ^b^	0.50 ± 0.01 ^a^	0.20 ± 0.01 ^b^	0.10 ± 0.01 ^b^	0.40 ± 0.01 ^a^
Palmitic acid	C16:0	27.50 ± 0.01 ^b^	30.80 ± 0.01 ^a^	22.80 ± 0.01 ^d^	23.00 ± 0.01 ^d^	19.30 ± 0.01 ^e^	18.00 ± 0.01 ^f^	24.90 ± 0.01 ^c^
Palmitoleic acid	C16:1	0.20 ± 0.01 ^bc^	0.30 ± 0.01 ^b^	0.50 ± 0.01 ^a^	0.30 ± 0.01 ^b^	0.40 ± 0.01 ^a^	0.40 ± 0.01 ^a^	0.50 ± 0.01 ^a^
Stearic acid	C18:0	3.80 ± 0.01 ^a^	2.80 ± 0.01 ^c^	3.10 ± 0.01 ^b^	3.30 ± 0.01 ^b^	3.80 ± 0.01 ^a^	1.80 ± 0.01 ^d^	2.70 ± 0.01 ^c^
Oleic acid	C18:1	6.50 ± 0.01 ^d^	9.20 ± 0.01 ^c^	5.90 ± 0.01 ^e^	4.90 ± 0.01 ^f^	16.70 ± 0.01 ^a^	13.20 ± 0.01 ^b^	2.50 ± 0.01 ^g^
Linoleic acid	C18:2	30.50 ± 0.01 ^a^	20.60 ± 0.01 ^c^	15.50 ± 0.01 ^e^	17.20 ± 0.01 ^d^	28.70 ± 0.01 ^b^	11.30 ± 0.01 ^g^	13.20 ± 0.01 ^f^
*α*-linolenic acid	C18:3	26.40 ± 0.01 ^e^	32.90 ± 0.01 ^d^	46.50 ± 0.01 ^b^	36.90 ± 0.01 ^c^	23.50 ± 0.01 ^f^	46.40 ± 0.01 ^b^	48.00 ± 0.01 ^a^
Arachidic acid	C20:0	0.70 ± 0.01 ^d^	0.50 ± 0.01 ^e^	0.50 ± 0.01 ^e^	1.00 ± 0.01 ^c^	1.20 ± 0.01 ^bc^	1.40 ± 0.01 ^b^	1.80 ± 0.01 ^a^
Cis-11-Eicosenoic acid	C20:1	0.20 ± 0.01 ^c^	0.20 ± 0.01 ^c^	<0.05 *	0.80 ± 0.01 ^a^	0.20 ± 0.01 ^c^	0.50 ± 0.01 ^b^	0.80 ± 0.01 ^a^
Cis-11.14 Eicosenoic acid	C20:2	<0.05 *	0.10 ± 0.01 ^b^	0.10 ± 0.01 ^b^	<0.05 *	0.30 ± 0.01 ^a^	0.10 ± 0.01 ^b^	<0.05 *
Eicosatrienoic acid	C20:3	<0.05 *	0.10 ± 0.01 ^b^	0.30 ± 0.01 ^a^	<0.05 *	<0.05 *	0.20 ± 0.01 ^ab^	<0.05 *
Arachidonic acid	C20:4	<0.05 *	0.20 ± 0.01 ^b^	0.60 ± 0.01 ^a^	<0.05 *	0.30 ± 0.01 ^b^	<0.05 *	<0.05 *
Heneicosylic acid	C21:0	<0.05 *	0.10 ± 0.01 ^a^	0.10 ± 0.01 ^a^	<0.05 *	<0.05 *	<0.05 *	<0.05 *
Behenic acid	C22:0	1.20 ± 0.01 ^c^	0.20 ± 0.01 ^e^	0.70 ± 0.01 ^d^	1.20 ± 0.01 ^c^	1.20 ± 0.01 ^c^	2.70 ± 0.01 ^a^	2.10 ± 0.01 ^b^
Tricosanoic acid	C23:0	<0.05 *	0.20 ± 0.01 ^b^	0.40 ± 0.01 ^a^	<0.05 *	0.10 ± 0.01 ^b^	<0.05 *	<0.05 *
Lignoceric acid	C24:0	1.30 ± 0.01 ^c^	0.60 ± 0.01 ^f^	1.60 ± 0.01 ^b^	1.40 ± 0.01 ^bc^	1.90 ± 0.01 ^a^	1.00 ± 0.01 ^d^	0.80 ± 0.01 ^e^
	SFA	36.10 ± 0.01 ^a^	36.30 ± 0.01 ^a^	30.50 ± 0.01 ^d^	35.50 ± 0.01 ^b^	29.90 ± 0.01 ^e^	27.90 ± 0.01 ^f^	34.60 ± 0.01 ^c^
	MUFA	7.00 ± 0.01 ^e^	9.80 ± 0.01 ^c^	6.40 ± 0.01 ^f^	8.20 ± 0.01 ^d^	17.30 ± 0.01 ^a^	14.10 ± 0.01 ^b^	4.20 ± 0.01 ^g^
	PUFA	56.90 ± 0.01 ^d^	53.90 ± 0.01 ^f^	63.10 ± 0.01 ^a^	56.30 ± 0.01 ^e^	52.80 ± 0.01 ^g^	58.00 ± 0.01 ^c^	61.20 ± 0.01 ^b^

SFA—total saturated fatty acids; MUFA—total monounsaturated fatty acids; and PUFA—total polyunsaturated fatty acids. Results are expressed in percentages of total methyl esters ± standard deviation (n = 2).* LOQ < 0.05 g/100 g. The letters (a–g) denote the significant differences between the halophytes using Tukey’s test (*p* < 0.05).

**Table 3:** Mineral composition of the halophyte species.

*Minerals*	*I. crithmoides*	*S. fruticosa*	*S. ramosissima*	*D. crassifolium*	*C. maritimum*	*M. nodiflorum*	*M. crystallinum*
*(mg/100 g FW)*							
Na	420 ± 54.60 ^bc^	1120 ± 145.60 ^a^	600 ± 78.00 ^b^	309 ± 40.17 ^c^	570 ± 74.10 ^b^	520 ± 67.60 ^bc^	391 ± 50.81 ^bc^
Ca	45.00 ± 5.40 ^a^	36.10 ± 4.33 ^b^	9.20 ± 1.10 ^c^	41.00 ± 4.92 ^a^	30.70 ± 3.70 ^b^	27.60 ± 3.30 ^b^	11.70 ± 1.40 ^c^
K	194 ± 40.70 ^b^	400 ± 84.00 ^a^	186 ± 39.00 ^b^	72 ± 15.00 ^c^	159 ± 33.90 ^b^	110 ± 23.10 ^bc^	58 ± 12.18 ^c^
Mg	45.60 ± 6.38 ^b^	55.60 ± 7.78 ^ab^	66.00 ± 9.34 ^a^	18.30 ± 2.56 ^c^	13.30 ± 1.86 ^c^	16.70 ± 2.33 ^c^	8.60 ± 1.20 ^c^
Cu	0.008 ± 0.0007 ^c^	0.110 ± 0.0009 ^a^	0.120 ± 0.0010 ^a^	0.008 ± 0.0007 ^c^	0.007 ± 0.0006 ^c^	0.016 ± 0.0010 ^bc^	0.006 ± 0.0005 ^c^
Fe	0.48 ± 0.07 ^c^	1.42 ± 0.19 ^a^	0.71 ± 0.09 ^bc^	0.22 ± 0.01 ^d^	0.73 ± 0.10 ^bc^	0.41 ± 0.05 ^cd^	0.32 ± 0.05 ^cd^
I	<0.03 *	<0.03 *	<0.03 *	<0.03 *	<0.03 *	<0.03 *	<0.03 *
Mn	0.16 ± 0.02 ^b^	0.47 ± 0.06 ^a^	0.57 ± 0.08 ^a^	0.12 ± 0.02 ^b^	0.16 ± 0.02 ^b^	0.24 ± 0.03 ^b^	0.16 ± 0.02 ^b^
Se	<0.02 *	<0.02 *	<0.02 *	<0.02 *	<0.02 *	<0.02 *	<0.02 *
Zn	0.18 ± 0.03 ^c^	0.50 ± 0.07 ^a^	0.36 ± 0.05 ^b^	0.12 ± 0.02 ^c^	0.14 ± 0.02 ^c^	0.26 ± 0.04 ^b^	0.05 ± 0.007 ^d^

Results are expressed as means values ± standard deviation (n = 3). * LOD: limit of detection µg/g. The letters (a–d) correspond to the significant difference between halophytes using Tukey’s test (*p* < 0.05).

**Table 4:** Potentially toxic metallic elements content in the halophyte species.

*Heavy Metals*	*I. crithmoides*	*S. fruticosa*	*S. ramosissima*	*D. crassifolium*	*C. maritimum*	*M. nodiflorum*	*M. crystallinum*
*(µg/g DW)*							
As	0.057 ± 0.004 ^d^	0.310 ± 0.001 ^a^	0.130 ± 0.001 ^bc^	<0.03 *	0.090 ± 0.002 ^cd^	0.170 ± 0.004 ^b^	<0.03 *
Cd	<0.10 *	1.22 ± 0.38	<0.10 *	<0.10 *	<0.10 *	<0.10 *	<0.10 *
Hg	<0.03 *	<0.03 *	<0.03 *	<0.03 *	<0.03 *	<0.03 *	<0.03 *
Pb	1.02 ± 0.05 ^c^	0.73 ± 0.02 ^d^	0.42 ± 0.03 ^e^	1.95 ± 0.05 ^b^	<0.03 *	0.44 ± 0.05 ^e^	2.99 ± 0.04 ^a^

Results are expressed as means values ± standard deviation (n = 3). * LOD: limit of detection µg/g. The letters (a–e) correspond to the significant difference between halophytes using Tukey’s test (*p* < 0.05).

**Table 5:** Total phenolic content (TPC) and antioxidant activity measured with Oxygen Radical Absorbance Capacity (ORAC) and Hydroxyl Radical Scavenging Capacity (HOSC) and antihypertensive activities (Angiotensin converting enzyme-ACE inhibition) in the halophyte species.

*Phytochemical Parameters*	*I. crithmoides*	*S. fruticosa*	*S. ramosissima*	*D. crassifolium*	*C. maritimum*	*M. nodiflorum*	*M. crystallinum*
***TPC* (mg GAE/g FW) **	0.25 ± 0.02 ^b^	0.33 ± 0.02 ^ab^	0.41 ± 0.09 ^a^	0.11 ± 0.007 ^c^	0.25 ± 0.04 ^b^	0.098 ± 0.003 ^c^	0.10 ± 0.005 ^c^
*Antioxidant activity*							
ORAC (µmol TEAC/g FW)	4.71 ± 1.35 ^bc^	8.17 ± 0.97 ^a^	10.27 ± 3.20 ^a^	2.69 ± 0.22 ^c^	8.51 ± 2.26 ^a^	1.08 ± 0.08 ^c^	1.28 ± 0.05 ^c^
HOSC (µmol TEAC/g FW)	4.16 ± 0.41 ^bc^	7.66 ± 1.00 ^a^	11.64 ± 4.40 ^a^	2.41 ± 0.35 ^c^	6.89 ± 0.95 ^a^	0.92 ± 0.09 ^c^	1.03 ± 0.05 ^c^
*Antihypertensive activity*							
ACE inhibition (IC_50_ = mg/mL)	93.01 ± 7.92 ^a^	95.61 ± 14.13 ^a^	102.30 ± 14.42 ^a^	175.61 ± 12.15 ^b^	561.50 ± 21.35 ^c^	73.98 ± 4.62 ^a^	80.79 ± 3.05 ^a^

Results are expressed as means values ± standard deviation (n = 3). The letters (a–c) indicate, per row, statistically significant differences between halophytes using Tukey’s test (*p* < 0.05).

**Table 6:** Quantified phenolic compounds’ families in the halophyte species.

Phenolic Compounds’ Families * *µg/g FW*	*I. crithmoides*	*S. fruticosa*	*S. ramosissima*	*D. crassifolium*	*C. maritimum*	*M. nodiflorum*	*M. crystallinum*
Hydroxycinnamic acids	9.478 ± 0.960 ^e^	40.138 ± 1.099 ^c^	102.462 ± 2.302 ^b^	30.726 ± 2.058 ^d^	191.869 ± 1.003 ^a^	6.050 ± 0.03 ^e^	37.786 ± 0.942 ^c^
Hydroxybenzoic acids	1.368 ± 0.059 ^c^	2.241 ± 0.304 ^b^	9.609 ± 0.202 ^a^	ND	1.508 ± 0.048 ^c^	0.974 ± 0.004 ^c^	ND
Phenolic acids	10.846 ± 0.078 ^f^	42.379 ± 0.092 ^c^	112.071 ± 0.178 ^b^	30.726 ± 0.275 ^e^	193.377 ± 0.112 ^a^	7.024 ± 0.004 ^g^	37.786 ± 0.109 ^d^
Flavone	0.319 ± 0.010 ^e^	ND	ND	1.684 ± 0.105 ^c^	10.863 ± 0.174 ^a^	0.803 ± 0.010 ^d^	7.806 ± 0.518 ^b^
Flavanol	0.716 ± 0.059 ^c^	11.773 ± 0.168 ^a^	0.299 ± 0.001 ^d^	0.862 ± 0.107 ^c^	ND	4.600 ± 0.017 ^b^	0.701 ± 0.007 ^c^
Flavanone	6.382 ± 0.176 ^c^	7.071 ± 0.114 ^b^	ND	ND	0.731 ± 0.048 ^e^	13.065 ± 0.162 ^a^	1.571 ± 0.273 ^d^
Flavonol	0.194 ± 0.082 ^g^	48.259 ± 0.270 ^a^	6.166 ± 0.459 ^d^	21.106 ± 0.203 ^b^	17.786 ± 0.183 ^c^	2.871 ± 0.010 ^e^	1.857 ± 0.142 ^f^
Flavanonol	ND	6.404 ± 0.792	ND	ND	ND	ND	ND
Flavonoids	7.611 ± 0.065 ^f^	73.507 ± 0.224 ^a^	6.465 ± 0.153 ^g^	23.652 ± 0.103 ^c^	29.38 ± 0.068 ^b^	21.339 ± 0.015 ^d^	11.935 ± 0.125 ^e^
Coumarin	ND	ND	ND	1.458 ± 0.215	ND	ND	ND
Monocarboxylic acid	1.828 ± 0.219	ND	ND	ND	ND	ND	ND
Gallotannin	ND	ND	ND	ND	ND	0.177 ± 0.001	ND
∑ Phenolic compounds	18.457 ± 0.074 ^f^	115.886 ± 0.137 ^b^	118.536 ± 0.174 ^b^	55.836 ± 0.225 ^c^	222.757 ± 0.094 ^a^	28.363 ± 0.010 ^e^	49.721 ± 0.114 ^d^

* Quantified phenolic compounds’ families, determined as the sum of individual phenolic compounds quantified in the different halophyte species (Table S1—Supplementary Materials). Results are expressed as mean ± standard deviation (n = 2), µg/g of fresh weight (FW). The hydroxycinnamic acids (HCA) and their derivatives were quantified as a 3-O-caffeoylquinic acid equivalent (CQAE), flavonoids were quantified as quercetin-3-glucoside equivalent (QGE), and hydroxybenzoic acids (HBA) were quantified as a gallic acid equivalent (GAE). The letters (a–g) indicate significant differences between the halophytes using Tukey’s test (*p* < 0.05). ND: not detected; LOQ = 0.01 µg compound/g FW.

## Data Availability

The data supporting the findings of this study are available within the article and its supplementary material.

## Supplementary Materials

The following supporting information can be downloaded at: https://www.mdpi.com/article/10.3390/antiox12061161/s1, Figure S1: Correlation matrix (Pearson’s R values from −1 to 1) between the phenolic composition and the bioactivity measured by in vitro antioxidant and anti-hypertensive activities. ACE inhibition expressed as inverse values for the anti-hypertensive activity. Table S1: Individual phenolic compounds quantified in the different halophyte species. Results are expressed as mean ± standard deviation (n = 2), µg/g of fresh weight (FW). The hydroxycinnamic acids (HCA) and their derivatives were quantified as a 3-O-caffeoylquinic acid equivalent (CQAE), flavonoids were quantified as quercetin-3-glucoside equivalent (QGE), and hydroxybenzoic acids (HBA) were quantified as a gallic acid equivalent (GAE). Table S2. References [153,154,155,156,157,158,159,160,161,162,163,164,165,166,167,168,169,170,171,172,173,174,175,176,177,178,179,180,181,182,183,184,185,186,187,188,189,190,191,192,193,194,195,196,197,198,199,200,201,202,203,204,205,206,207,208,209,210,211,212,213,214,215,216,217,218,219,220,221,222,223,224,225,226,227,228,229,230,231,232,233,234,235,236,237,238,239,240] are cited in the Supplementary Materials.

## Appendix A

**Table A1:** Phenolic compounds, sorted by ascending retention time (RT), putatively identified in the halophyte extracts, IC (*I. crithmoides*), SF (*S. fruticosa*), SR (*S. ramosissima*), DC (*D. crassifolium*), CM (*C. maritimum*), MN (*M. nodiflorum*), and MC (*M. crystallinum*). HCA—Hydroxycinnamic acid; HBA—Hydroxybenzoic acid. * Phenolic compound identified with standard.

Peak	RT (min)	λ_max_ (nm)	[M-H]^−^, *m*/*z*	HPLC–DAD–ESI-MS/MS *m*/*z* (% Base Peak)	Tentative Identification	Compounds’ Class	References	Plant Extracts
1	18.72	275	261	261: 181(20), 163(15), 135(10), 119(5), 97(100)	*p*-Coumaric acid derivative	HCA	[95,102,103]	SF
2	26.67	289	315	315: 153(100), 109(80)	Protocatechuic acid-glucoside	HBA	[103,104,105]	IC,CM
3	27.8	275	343	343: 191(10)	5-Galloylquinic acid	HBA	[106]	SF
4	28.17	281	407	407: 407(60), 244(30), 169(10), 165(20), 125(10)	Gallic acid derivative	HBA	[102,107,108]	IC
5	29.7	296,325	353	353: 191(100), 179(85), 135(60)	5-O-Caffeoylquinic acid	HCA	[11,109,110]	SF,SR
6	29.72	259,298	197	197: 197(65), 182(60), 167(40), 153(25), 123(100), 121(20)	Syringic acid	HBA	[102,103]	IC
7	30.5	282, 345	315	315: 300(40), 151(40)	Rhamnetin	Flavonol	[111,112]	IC
8	32.63	307	259	259: 179(65), 135(100)	Caffeic acid derivative	HCA	[103,112]	IC,SF
9	32.81	317	285	285: 153(30), 152(100), 108(100), 109(20)	Protocatechuic-acid-arabinoside	HBA	[102,103,112]	SF,SR
10	33.95	280,310	305	305: 225(30), 97(100), 59(75)	Gallocatechin	Flavanol	[102,113]	IC,SF,SR,MN,MC
11	34.03	301	341	341: 179(30), 135(100)	Caffeic acid-O-glucoside	HCA	[102,113,114]	IC,CM
12	35.16	272	325	325: 163(40), 119(10)	*p*-Coumaric acid-O-glucoside	HCA	[102,103,115]	CM,MN,MC
13	35.62	274	163	163: 119(100)	*p*-Coumaric acid	HCA	[95,102,108]	IC,SR,DC
14	36.69	276,310	305	305: 219(10), 179(10), 221(10), 261(10)	Epigallocatechin	Flavanol	[105]	DC,MN,MC
15	37.46	296,326	353	353: 191(100), 179(10), 173(20)	3-O-Caffeoylquinic acid *	HCA	[11,95,104,116,117]	IC,SF,SR,CM
16	38.14	328	355	355: 253(50), 181(60), 165(10), 143(10), 107(20)	Pinobanksin-3-O-pentanoate	Flavanone	[118,129]	CM,MC
17	38.23	284	455	455: 455(80), 169(40), 125(20)	Gallic acid derivative	HBA	[102,107,108]	IC
18	38.49	269,330	355	355: 193(30), 178(15), 161(20), 134(10)	Ferulic acid-glucoside	HCA	[102,103,119]	SR,DC,MN,MC
19	38.95	279,330	321	321: 193(15), 119(25)	Ferulic acid derrivative	HCA	[102,103,119]	MN
20	38.95	268,325	355	355: 179(30), 134(15)	Caffeic acid glucuronide	HCA	[103,119,120]	DC
21	39.03	284,324	411	411: 411(100), 179(15), 135(35)	Caffeic acid derivative	HCA	[103,114,119]	IC
22	39.07	284,318	193	193: 134(10), 161(50), 178(5)	Ferulic acid	HCA	[103,119,120]	SR
23	39.15	275	387	387: 387(85), 163(20), 119(65)	*p*-Coumaric acid derivative	HCA	[95,102,103]	SF
24	39.30	280,319	355	355: 193(20), 178(40), 135(20)	Ferulic acid derivative	HCA	[102,103,119]	MC
25	39.33	270,330	593	593: 593(100), 341(5), 311(10)	Apigenin 6-C-glucoside-7-O-glucoside	Flavone	[121]	CM
26	39.72	280	385	385: 223(100), 208(40), 164(40)	Sinapic acid-glucoside	HCA	[122,123,124]	MC
27	40.30	268,340,447	433	433: 271(40), 301(20), 151(20)	Avicularin	Flavonol	[125,126]	MN
28	40.32	273,325	351	351: 351(40), 145(10), 307(10)	Coumarin glycoside ester	Coumarin	[127]	DC
29	40.38	269,330	385	385: 191(10), 173(50)	Feruloylglucaric acid	HCA	[128]	MC
30	40.40	269,325	455	455: 353(30), 179(50), 173(30), 191(20)	Caffeoylquinic acid derivative	HCA	[95,116,119]	SF
31	40.42	280	327	327: 285(40), 267(10), 239(20), 180(20), 165(30), 139(50)	Pinobanksin-5-methyl ether-3-O-acetate (isomer)	Flavanone	[118,129]	IC
32	41.05	268,311	209	209: 179(65), 135(30)	Caffeic acid derivative	HCA	[103,114,119]	SF
33	41.12	279,325	387	387: 255(80), 211(20), 213(10), 151(40)	Pinocembrin derivative	Flavanone	[129,130]	MN
34	41.21	278,320	259	259: 163(50), 119(20)	*p*-Coumaric acid derivative	HCA	[95,102,103]	SR,CM
35	41.23	277,324	433	433: 417(40), 285(60), 229(50), 151(40)	Kaempferol derivative	Flavonol	[111,118,130]	MC
36	41.60	270,332	303	303: 303(5), 97(100)	Dihydroquercetin	Flavanonol	[131]	SF
37	41.80	280,330	423	423: 197(80), 182(40), 167(45), 152(45), 125(45)	Syringic acid derivative	HBA	[102,103]	MN
38	42.37	288,312	337	337: 191(100), 173(20), 163(25)	*p*-Coumaroylquinic acid (isomer 1)	HCA	[11,132,133,134]	SF,SR,CM,MC
39	43.57	284,330	391	391: 337(90), 163(10)	*p*-Coumaric acid derivative	HCA	[95,102,103]	MN
40	44.22	284,320	443	443: 193(45), 134(30), 178(10)	Ferulic acid derivative	HCA	[102,103,119]	SR
41	44.27	302	337	337: 191(100), 179(20), 173(10), 163(5)	*p*-Coumaroylquinic acid (isomer 2)	HCA	[11,132,133,134]	SR,CM
42	44.32	280,320	547	547: 487(100), 529(10), 457(10), 427(10), 367(15), 337(15)	Chrysin-6-C-ara-8-C-glu	Flavone	[135]	MN
43	44.65	284,324	367	367: 247(55), 193(15), 191(100)	Feruloylquinic acid	HCA	[122,127,128]	IC
44	43.17	280,320	499	499: 337(40), 163(10), 111(5), 93(5)	3-O-*p*-Coumaroyl-5-O-caffeoylquinic acid	HCA	[136]	IC,SF
45	45.83	255,352	609	609: 609(100), 429(30), 301(5), 300(100)	Quercetin-3-O-rutinoside	Flavonol	[128,132,134]	CM
46	45.88	270,333	607	607: 487(30)	Acacetin 3,6-di-C-glucoside	Flavone	[137]	MC
47	46.15	280,311	289	289: 163(10), 119(10)	*p*-Coumaric acid derivative	HCA	[95,102,103]	DC
48	46.2	314	449	449: 287(50), 151(30), 135(15)	Eriodictyol-O-hexoside	Flavanone	[109,119,138]	SF,MN
49	46.2	279,315	287	287: 135(50), 151(20), 107(10)	Erydictiol	Flavanone	[138]	MN
50	46.3	282,311	547	547: 529(10), 337(10), 367(20)	Chrysin-6-C-glucosyl-8-C-arabinoside	Flavone	[139]	MC
51	46.78	279,332	307	307: 289(60), 245(20), 179(20), 205(10)	Epicatechin hydrate	Flavanol	[140]	MN
52	46.87	280	419	305: 305(80), 225(10), 97(50)	Gallocatechin derivative	Flavanol	[113]	IC
53	47.72	255,355	463	463: 463(50), 301(55), 300(100)	Quercetin 3-O-glucoside *	Flavonol	[11,95,116]	SR,CM
54	47.12	314	581	581: 163(100), 119(10)	*p*-Coumaric acid derivative	HCA	[95,102,103]	MC
55	47.25	274,320	437	437: 437(40), 289(20)	Epicatechin derivative	Flavanol	[105,126,141]	SF
56	47.65	311	559	559: 163(40)	*p*-Coumaric acid derivative	HCA	[95,102,103]	MC
57	47.73	284	369	369: 369(10), 255(10), 193(40), 179(15), 165(100), 107(30)	Piscidic acid derivative	Monocarboxylic acid	[142]	IC
58	47.78	260,352	767	767: 623(100), 315(20)	Isorhamnetin-rutinoside derivative	Flavonol	[109,135,142]	DC
59	47.95	255,265,350	609	609: 609(100), 477(30), 459(10), 315(60), 299(30), 165(25)	Rhamnetin hexosyl pentoside	Flavonol	[143]	SF
60	48.14	265,325	431	431: 413(5), 341(15), 312(100), 311(20)	Apigenin 6-C-glucoside	Flavone	[109,136,138]	CM
61	48.5	296,326	515	515: 353(100), 335(20), 191(25), 179(55), 173(80)	3,4-Dicaffeoylquinic acid	HCA	[11,95,116]	SR
62	48.5	319	589	589: 325(60), 163(10)	*p*-Coumaric acid glucoside derivative	HCA	[144]	MC
63	48.67	280,320	641	641: 495(10), 191(10)	Digalloyl quinic acid rhamnoside	HBA	[145,146]	MN
64	48.82	260, 351	393	393: 299(40), 255(25), 277(20), 285(10)	Luteolin derivative	Flavone	[118,138,147]	DC
65	49.07	254,266,352	623	623: 623(100), 477(10), 487(25), 315(35), 215(10), 214(40)	Isorhamnetin 3-O-robinobioside	Flavonol	[148]	SF
66	49.23	284	395	395: 395(100), 163(10), 119(20)	*p*-Coumaric acid derivative	HCA	[95,102,103]	IC
67	49.25	296,326	515	515: 353(100), 191(85), 179(55)	3,5-Dicaffeoylquinic acid	HCA	[11,122,134]	SR,CM
68	49.5	270,286,334	607	607: 607(20), 299(100)	Diosmetin 7-O-rutinoside	Flavone	[138,149]	CM
69	49.57	280,352	565	565: 301(10), 300(20), 179(30), 151(10)	Quercetin dipentoside	Flavonol	[143]	MN
70	49.60	272,354	575	575: 463(15), 300(80), 301(40), 179(20), 151(10)	Quercetin-3-O-glucoside derivative	Flavonol	[11,134,138]	MC
71	50.08	307	429	429: 391(100), 337(40), 173(80), 163(15), 119(20)	*p*-Coumaric acid derivative	HCA	[95,102,103]	SF
72	50.30	279,352	415	415: 301(40), 300(10)	Quercetin derivative	Flavonol	[110,119,147]	MN
73	50.35	260, 352	621	621: 477(100), 315(90), 519(88), 559(30)	Isorhamnetin-glucoside derivative	Flavonol	[142,147,148]	DC
74	50.62	294,326	515	515: 353(100), 191(15), 179(55), 173(80)	4,5-Dicaffeoylquinic acid	HCA	[11,95,149]	SR
75	50.92	278,319	335	335: 335(90), 163(100)	*p*-Coumaric acid derivative	HCA	[95,102,103]	SF
76	50.98	280,325	431	431: 311(35), 341(30), 283(20), 269(10), 243(10), 209(10)	Vitexin (apigenin-8-C-glu)	Flavone	[109,132,136]	IC
77	51.37	279,321	561	561: 561(100), 337(50), 163(60), 119(40)	*p*-Coumaric acid derivative	HCA	[95,102,103]	SF
78	51.77	285	475	475: 163(100)	*p*-Coumaric acid derivative	HCA	[95,102,103]	CM
79	51.82	280,330	481	481: 481(100), 301(10), 275(10)	Hexahydroxydiphenoyl-Glucose	Gallotannin	[150]	MN
80	51.87	294,326	517	517: 355(100), 191(15), 181(55), 173(80)	Caffeoylhydrocaffeoylquinic acid	HCA	[116]	SR
81	52.47	281,324	517	517: 397(15), 355(15), 179(35), 135(35)	Caffeic acid-glucuronide-glucoside (isomer 2)	HCA	[11,118,119]	IC
82	52.48	284,322	955	955: 955(100), 477(5)	Isorhamnetin-3-O-glucoside (dimer)	Flavonol	[142,147,148]	SR
83	52.88	285	901	901: 739(40), 593(85), 285(10)	Kaempferol 3-(2″-[gluc-(1->3)-rhamn]-6″-rhamnosylgalactoside)	Flavonol	[151]	MC
84	52.93	290	565	565: 353(70), 179(10), 111(20), 191(10)	Caffeoylquinic acid derivative	HCA	[11,95,103]	MN
85	53.85	279,325	565	565: 299(30), 343(10), 169(15)	Galloylquinic acid derivative	HBA	[106,112,136]	MN
86	54.02	328	799	799: 601(80), 191(20), 515(20), 173(15), 179(10)	Malonyl-3,4-O-dicaffeoylquinic acid derivative	HCA	[152]	DC
87	55.4	279,325	575	575: 431(20), 311(10), 161(10), 215(10)	Vitexin derivative	Flavone	[109,132,136]	MN
88	56.62	317	525	525: 163(100), 119(10)	*p*-Coumaric acid derivative	HCA	[95,102,103]	DC
89	57.05	325	555	555: 193(100), 134(10)	Ferulic acid derivative	HCA	[102,103,141]	DC
90	60.80	328	757	757: 555(100), 193(100), 134(10)	Ferulic acid derivative	HCA	[102,103,141]	DC
91	61.37	280	575	575: 343(30), 191(50), 169(20)	Galloylquinic acid derivative	HBA	[106,112,136]	MN
92	62.87	280,320	327	327: 285(40), 267(10), 239(10), 195(40), 180(40)	Pinobanksin-5-methyl ether-3-O-acetate (isomer)	Flavanone	[129,130]	MN
93	64.05	317	539	539: 419(80), 163(100)	*p*-Coumaric acid derivative	HCA	[95,102,103]	DC
94	64.28	279,325	543	543: 261(10), 191(10), 349(10), 367(10)	3,5-Diferuoylquinic acid	HCA	[135]	MN

## References

[1] Mishra A., Tanna B. (2017). Halophytes: Potential Resources for Salt Stress Tolerance Genes and Promoters. Front Plant Sci..

[2] FAO (2022). Halt soil salinization, boost soil productivity. Proceedings of the Global Symposium on Salt-Affected Soils.

[3] FAO Global Map of Salt-Affected Soils. GSASmap. https://www.fao.org/soils-portal/data-hub/soil-maps-and-databases/global-map-of-salt-affected-soils/en/.

[4] Flowers T.J., Muscolo A. (2015). Introduction to the Special Issue: Halophytes in a changing world. AoB Plants.

[5] Flowers T.J., Colmer T.D. (2008). Salinity tolerance in halophytes. New Phytol..

[6] Duarte B., Caçador I. (2021). Iberian Halophytes as Agroecological Solutions for Degraded Lands and Biosaline Agriculture. Sustainability.

[7] Castañeda-Loaiza V., Oliveira M., Santos T., Schüler L., Lima A., Gama F., Salazar M., Neng N., Nogueira J., Varela J. (2020). Wild vs cultivated halophytes: Nutritional and functional differences. Food Chem..

[8] Duarte B., Santos D., Caçador I. (2013). Halophyte anti-oxidant feedback seasonality in two salt marshes with different degrees of metal contamination: Search for an efficient biomarker. Funct. Plant Biol..

[9] Duarte B., Caetano M., Almeida P.R., Vale C., Caçador I. (2010). Accumulation and biological cycling of heavy metal in four salt marsh species from Tagus Estuary (Portugal). Environ. Pollut..

[10] Barreira L., Resek E., Rodrigues M.J., Rocha M.I., Pereira H., Bandarra N., da Silva M.M., Varela J., Custódio L. (2017). Halophytes: Gourmet food with nutritional health benefits?. J. Food Compos. Anal..

[11] Oliveira-Alves S.C., Andrade F., Prazeres I., Silva A.B., Capelo J., Duarte B., Caçador I., Coelho J., Serra A.T., Bronze M.R. (2021). Impact of Drying Processes on the Nutritional Composition, Volatile Profile, Phytochemical Content and Bioactivity of *Salicornia ramosissima.* J. Woods. Antioxidants.

[12] Calvo M.M., Martín-Diana A.B., Rico D., López-Caballero M.E., Martínez-Álvarez O. (2022). Antioxidant, Antihypertensive, Hypoglycaemic and Nootropic Activity of a Polyphenolic Extract from the Halophyte Ice Plant (*Mesembryanthemum crystallinum*). Foods.

[13] Duarte B., Santos D., Marques J.C., Caçador I. (2013). Ecophysiological adaptations of two halophytes to salt stress: Photosynthesis, PS II photochemistry and anti-oxidant feedback—Implications for resilience in climate change. Plant Physiol. Biochem..

[14] Duarte B., Sleimi N., Caçador I. (2014). Biophysical and biochemical constraints imposed by salt stress: Learning from halophytes. Front. Plant Sci..

[15] Onakpoya I.J., Spencer E.A., Thompson M.J., Heneghan C.J. (2015). The effect of chlorogenic acid on blood pressure: A systematic review and meta-analysis of randomized clinical trials. J. Hum. Hypertens..

[16] Maaliki D., Shaito A.A., Pintus G., El-Yazbi A., Eid A.H. (2019). Flavonoids in hypertension: A brief review of the underlying mechanisms. Curr. Opin. Pharmacol..

[17] Men R., Li N., Xing Y., Tang Y., Tan C., Meng F., Zhang J., Ni H., Jia X. (2013). Chemical constituents and ACE inhibitory activity of desert plant *Suaeda physophora* Pall. Acta Pharm. Sin. B.

[18] Jenis J., Kim J.Y., Uddin Z., Song Y.H., Lee H.-H., Park K.H. (2017). Phytochemical profile and angiotensin I converting enzyme (ACE) inhibitory activity of *Limonium michelsonii* Lincz. J. Nat. Med..

[19] Akinniyi G., Lee J., Kim H., Lee J.-G., Yang I. (2022). A Medicinal Halophyte *Ipomoea pes-caprae* (Linn.) R. Br.: A Review of Its Botany, Traditional Uses, Phytochemistry, and Bioactivity. Mar. Drugs.

[20] Paiva L., Lima E., Neto A.I., Baptista J. (2016). Angiotensin I-converting enzyme (ACE) inhibitory activity of *Fucus spiralis* macroalgae and influence of the extracts storage temperature—A short report. J. Pharm. Biomed. Anal..

[21] Santos M.C., Toson N.S.B., Pimentel M.C.B., Bordignon S.A.L., Mendez A.S.L., Henriques A.T. (2020). Polyphenols composition from leaves of *Cuphea* spp. and inhibitor potential, *in vitro*, of angiotensin I-converting enzyme (ACE). J. Ethnopharmacol..

[22] Zhang Y., Lee E.T., Devereux R.B., Yeh J., Best L.G., Fabsitz R.R., Howard B.V. (2006). Prehypertension, diabetes, and cardiovascular disease risk in a population-based sample: The strong heart study. Hypertension.

[23] AOAC Association of Official Analytical Chemists (2012). Official Methods of Analysis of AOAC International.

[24] Cruz de Carvalho R., Feijão E., Kletschkus E., Marques J.C., Reis-Santos P., Fonseca V.F., Papenbrock J., Caçador I., Duarte B. (2020). Halophyte bio-optical phenotyping: A multivariate photochemical pressure index (Multi-PPI) to classify salt marsh anthropogenic pressures levels. Ecol. Indic..

[25] Sghaier D.B., Pedro S., Diniz M.S., Duarte B., Caçador I., Sleimi N. (2016). Tissue localization and distribution of as and al in the halophyte *Tamarix gallica* under controlled conditions. Front. Mar. Sci..

[26] Towett E.K., Shepherd K.D., Cadisch G. (2013). Quantification of total element concentrations in soils using total X-ray fluorescence spectroscopy (TXRF). Sci. Total Environ..

[27] Castello G. (1999). Retention index systems: Alternatives to the n-alkanes as calibration standards. J. Chromatogr. A.

[28] Van Den Dool H., Kratz P.D. (1963). A Generalization of the Retention Index System including Linear Temperature Programmed Gas-Liquid Partition Chromatography. J. Chromatogr..

[29] Swain T., Hillis W.E. (1959). The phenolic constituents of *Prunus domestica*. I.—The quantitative analysis of phenolic constituents. J. Sci. Food Agric..

[30] Singleton V.L., Rossi J.A. (1965). Colorimetry of Total Phenolics with Phosphomolybdic-Phosphotungstic Acid Reagents. Am. J. Enol. Vitic..

[31] Huang D., Ou B., Hampsch-Woodill M., Flanagan J.A., Prior R.L. (2002). High-throughput assay of oxygen radical absorbance capacity (ORAC) using a multichannel liquid handling system coupled with a microplate fluorescence reader in 96-well format. J. Agric. Food Chem..

[32] Serra A.T., Duarte R.O., Bronze M.R., Duarte C.M.M. (2011). Identification of bioactive response in traditional cherries from Portugal. Food Chem..

[33] Moore J., Yin J.J., Yu L. (2006). Novel fluorometric assay for hydroxyl radical scavenging capacity (HOSC) estimation. J. Agric. Food Chem..

[34] Mecha E., Feliciano R.P., Rodriguez-Mateos A., Silva S.D., Figueira M.E., Vaz Patto M.C., Bronze M.R. (2020). Human bioavailability of phenolic compounds found in common beans: The use of high resolution mass spectrometry to evaluate inter individual variability. Br. J. Nutr..

[35] Zaier M.M., Ciudad-Mulero M., Cámara M., Pereira C., Ferreira I.C.F.R., Achour L., Kacem A., Morales P. (2020). Revalorization of Tunisian wild *Amaranthaceae* halophytes: Nutritional composition variation at two different phenotypes stages. J. Food Compos. Anal..

[36] Ghazanfar S.A., Altundag E., Yaprak A.E., Osborne J., Tug G.N., Vural M., Khan M.A., Böer B., Öztürk M., Al Abdessalaam T.Z., Clüsener-Godt M., Gul B. (2014). Halophytes of Southwest Asia. Sabkha Ecosystems.

[37] Dajic Z., Madhava Rao K., Raghavendra A., Janardhan Reddy K. (2006). Salt Stress. Physiology and Molecular Biology of Stress Tolerance in Plants.

[38] Rocha M.I., Rodrigues M.J., Pereira C., Pereira H., da Silva M.M., Neng N.d.R., Nogueira J.M.F., Varela J., Barreira L., Custódio L. (2017). Biochemical profile and in vitro neuroprotective properties of *Carpobrotus edulis* L., a medicinal and edible halophyte native to the coast of South Africa. S. Afr. J. Bot..

[39] Borah S., Baruah A.M., Das A.K., Borah J. (2009). Determination of mineral content in commonly consumed leafy vegetables. Food Anal. Methods.

[40] Custódio M., Lillebø A.I., Calado R., Villasante S. (2021). Halophytes as novel marine products—A consumers’ perspective in Portugal and policy implications. Mar. Policy.

[41] Lopes M., Roque M.J., Cavaleiro C., Ramos F. (2021). Nutrient value of *Salicornia ramosissima*—A green extraction process for mineral analysis. J. Food Compos. Anal..

[42] Krajcovicova-Kudlackova M., Babinska K., Valachovicova M. (2005). Health benefits and risks of plant proteins. Bratisl Lek Listy.

[43] Lima A.R., Gama F., Castañeda-Loaiza V., Costa C., Schüler L.M., Santos T., Salazar M., Nunes C., Cruz R.M.S., Varela J. (2021). Nutritional and Functional Evaluation of *Inula crithmoides* and *Mesembryanthemum nodiflorum* Grown in Different Salinities for Human Consumption. Molecules.

[44] Anderson J.W., Baird P., Davis R.H., Ferreri S., Knudtson M., Koraym A., Waters V., Williams C.L. (2009). Health benefits of dietary fiber. Nutr. Rev..

[45] European Commission, Regulation (EC) No 1924/2006 of 20 December 2006 on Nutrition and Health Claims Made on Foods. http://data.europa.eu/eli/reg/2006/1924/oj.

[46] Ventura Y., Eshel A., Pasternak D., Sagi M. (2015). The development of halophyte-based agriculture: Past and present. Ann. Bot..

[47] Vizetto-Duarte C., Figueiredo F., Rodrigues M.J., Polo C., Rešek E., Custódio L. (2019). Sustainable Valorization of Halophytes from the Mediterranean Area: A Comprehensive Evaluation of Their Fatty Acid Profile and Implications for Human and Animal Nutrition. Sustainability.

[48] Patel M.K., Pandey S., Brahmbhatt H.R., Mishra A., Jha B. (2019). Lipid content and fatty acid profile of selected halophytic plants reveal a promising source of renewable energy. Biomass Bioenergy.

[49] Centofanti T., Bañuelos G. (2019). Practical uses of halophytic plants as sources of food and fodder. Halophytes and Climate Change: Adaptive Mechanisms and Potential Uses.

[50] Maciel E., Domingues P., Domingues M.R.M., Calado R., Lillebø A. (2020). Halophyte plants from sustainable marine aquaponics are a valuable source of omega-3 polar lipids. Food Chem..

[51] Duarte B., Feijão E., Pinto M.V., Matos A.R., Silva A., Figueiredo A., Fonseca V.F., Reis-Santos P., Caçador I. (2022). Nutritional valuation and food safety of endemic mediterranean halophytes species cultivated in abandoned salt pans under a natural irrigation scheme. Estuar. Coast. Shelf Sci..

[52] Barbosa K.B.F., Volp A.C.P., Renhe I.R.T., Stringheta P.C. (2007). Omega-3 and 6 fatty acids and implications on human health. Nutr. Rev. Soc. Bras. Alim. Nutr..

[53] Duarte B., Carreiras J., Pérez-Romero J.A., Mateos-Naranjo E., Redondo-Gómez S., Matos A.R., Marques J.C., Caçador I. (2018). Halophyte fatty acids as biomarkers of anthropogenic-driven contamination in Mediterranean marshes: Sentinel species survey and development of an integrated biomarker response (IBR) index. Ecol. Indic..

[54] Clavel-Coibrié E., Sales J.R., da Silva A.M., Barroca M.J., Sousa I., Raymundo A. (2021). *Sarcocornia perennis*: A Salt Substitute in Savory Snacks. Foods.

[55] Alfheeaid H.A., Raheem D., Ahmed F., Alhodieb F.S., Alsharari Z.D., Alhaji J.H., BinMowyna M.N., Saraiva A., Raposo A. (2022). *Salicornia bigelovii*, *S. brachiate* and *S. herbacea*: Their Nutritional Characteristics and an Evaluation of Their Potential as Salt Substitutes. Foods.

[56] Houston M.C., Harper K.J. (2008). Potassium, Magnesium, and Calcium: Their Role in Both the Cause and Treatment of Hypertension. J. Clin. Hypertens..

[57] Castro H., Raij L. (2013). Potassium in Hypertension and Cardiovascular Disease. Semin. Nephrol..

[58] Adrogué H.J., Madias N.E. (2007). Sodium and Potassium in the Pathogenesis of Hypertension. N. Engl. J. Med..

[59] Schutten J.C., Joosten M.M., de Borst M.H., Bakker S.J.L. (2018). Magnesium and Blood Pressure: A Physiology-Based Approach. Adv. Chronic Kidney Dis..

[60] Renna M. (2018). Reviewing the Prospects of Sea Fennel (*Crithmum maritimum* L.) as Emerging Vegetable Crop. Plants.

[61] Cardoso M., Silva H., Patinha C., Costa N., Nunes S., Cunha A. (2021). From the saltpan to the plate: An evaluation of the use of the edible halophyte *Salicornia ramosissima* in catering. Ann. Appl. Biol..

[62] Pedro S., Duarte B., Almeida P.R., Caçador I. (2015). Metal speciation in salt marsh sediments: Influence of halophyte vegetation in salt marshes with different morphology. Estuar. Coast. Shelf Sci..

[63] European Commission, Regulation (EC) No 1881/2006 of 19 December 2006 Setting Maximum Levels for Certain Contaminants in Foodstuffs. http://data.europa.eu/eli/reg/2006/1881/oj.

[64] European Commission, Regulation (EC) No 2015/1006 of 25 June 2015 Amending Regulation (EC) No 1881/2006 as Regards Maximum Levels of Inorganic Arsenic in Foodstuffs. http://data.europa.eu/eli/reg/2015/1006/oj.

[65] Sánchez-Gavilán I., Chueca E.R., García V.F. (2021). Bioactive compounds in *Sarcocornia* and *Arthrocnemum*, two wild halophilic genera from the Iberian Peninsula. Plants.

[66] Hanen F., Riadh K., Samia O., Sylvain G., Christian M., Chedly A. (2009). Interspecific variability of antioxidant activities and phenolic composition in *Mesembryanthemum genus*. Food Chem. Toxicol..

[67] Al Shukor N., Van Camp J., Gonzales G.B., Staljanssens D., Struijs K., Zotti M.J., Raes K., Smagghe G. (2013). Angiotensin-Converting Enzyme Inhibitory Effects by Plant Phenolic Compounds: A Study of Structure Activity Relationships. J. Agric. Food Chem..

[68] Aluko R.E. (2015). Structure and function of plant protein-derived antihypertensive peptides. Curr. Opin. Food Sci..

[69] Barashkova A.S., Rogozhin E.A. (2020). Isolation of antimicrobial peptides from different plant sources: Does a general extraction method exist?. Plant Methods.

[70] Oliveira M., Sales P.A., Rodrigues M.J., DellaGreca M., Barreira L., Murta S.M., Romanha A.J., Custódio L. (2016). Unlocking the in vitro anti-Trypanosoma cruzi activity of halophyte plants from the southern Portugal. Asian Pac. J. Trop. Med..

[71] Custodio L., Garcia-Caparros P., Pereira C.G., Castelo-Branco P. (2022). Halophyte Plants as Potential Sources of Anticancer Agents: A Comprehensive Review. Pharmaceutics.

[72] Ferreira M.J., Pinto D.C.G.A., Cunha Â., Silva H. (2022). Halophytes as Medicinal Plants against Human Infectious Diseases. Appl. Sci..

[73] Rodrigues M.J., Jekő J., Cziáky Z., Pereira C.G., Custódio L. (2022). The Medicinal Halophyte *Frankenia laevis* L. (Sea Heath) Has In Vitro Antioxidant Activity, α-Glucosidase Inhibition, and Cytotoxicity towards Hepatocarcinoma Cells. Plants.

[74] eHALOPH Database of Halophytes and Other Salt-Tolerant Plants. https://ehaloph.uc.pt/listplants.

[75] Kumar N., Goel N. (2019). Phenolic acids: Natural versatile molecules with promising therapeutic applications. Biotechnol. Rep..

[76] Correia A., Silva A.M., Moreira M.M., Salazar M., Švarc-Gajić J., Brezo-Borjan T., Cádiz-Gurrea M.D.L.L., Carretero A.S., Loschi F., Dall’Acqua S. (2022). *Salicornia ramosissima*: A New Green Cosmetic Ingredient with Promising Skin Effects. Antioxidants.

[77] Silva A., Lago J., Pinto D., Moreira M., Grosso C., Fernandes V., Delerue-Matos C., Rodrigues F. (2021). *Salicornia ramosissima* Bioactive Composition and Safety: Eco-Friendly Extractions Approach (Microwave-Assisted Extraction vs. Conventional Maceration). Appl. Sci..

[78] Pungin A., Lartseva L., Loskutnikova V., Shakhov V., Krol O., Popova E., Kolomiets A., Nikolaeva N., Volodina A. (2022). The Content of Certain Groups of Phenolic Compounds and the Biological Activity of Extracts of Various Halophyte Parts of *Spergularia marina* (L.) Griseb. and *Glaux maritima* L. at Different Levels of Soil Salinization. Plants.

[79] Guerreiro Pereira C., Barreira L., Neng N., Nogueira J., Marques C., Santos T., Varela J., Custódio L. (2017). Searching for new sources of innovative products for the food industry within halophyte aromatic plants: In vitro antioxidant activity and phenolic and mineral contents of infusions and decoctions of *Crithmum maritimum* L.. Food Chem. Toxicol..

[80] Kim S., Lee E.-Y., Hillman P.F., Ko J., Yang I., Nam S.-J. (2021). Chemical Structure and Biological Activities of Secondary Metabolites from *Salicornia europaea* L.. Molecules.

[81] Limongelli F., Crupi P., Clodoveo M.L., Corbo F., Muraglia M. (2022). Overview of the Polyphenols in *Salicornia*: From Recovery to Health-Promoting Effect. Molecules.

[82] Politeo O., Popović M., Veršić Bratinčević M., Kovačević K., Urlić B., Generalić Mekinić I. (2023). Chemical Profiling of Sea Fennel (*Crithmum maritimum* L., Apiaceae) Essential Oils and Their Isolation Residual Waste-Waters. Plants.

[83] Lopes M., Silva A.S., Séndon R., Barbosa-Pereira L., Cavaleiro C., Ramos F. (2023). Towards the Sustainable Exploitation of Salt-Tolerant Plants: Nutritional Characterisation, Phenolics Composition, and Potential Contaminants Analysis of *Salicornia ramosissima* and *Sarcocornia perennis alpini*. Molecules.

[84] Siracusa L., Kulisic-Bilusic T., Politeo O., Krause I., Dejanovic B., Ruberto G. (2011). Phenolic composition and antioxidant activity of aqueous infusions from *Capparis spinosa* L. and *Crithmum maritimum* L. before and after submission to a two-step in vitro digestion model. J. Agric. Food Chem..

[85] Qasim M., Abideen Z., Adnan M.Y., Gulzar S., Gul B., Rasheed M., Khan M.A. (2017). Antioxidant properties, phenolic composition, bioactive compounds and nutritive value of medicinal halophytes commonly used as herbal teas. South Afr. J. Bot..

[86] Panche A.N., Diwan A.D., Chandra S.R. (2016). Flavonoids: An overview. J. Nutr. Sci..

[87] Finger A., Engelhardt U.H., Wray V. (1991). Flavonol triglycosides containing galactose in tea. Phytochemistry.

[88] Zampini I.C., Salas A.L., Maldonado L.M., Simirgiotis M.J., Isla M.I. (2021). Propolis from the Monte Region in Argentina: A Potential Phytotherapic and Food Functional Ingredient. Metabolites.

[89] Kramberger K., Barlič-Maganja D., Bandelj D., Baruca Arbeiter A., Peeters K., Miklavčič Višnjevec A., Jenko Pražnikar Z. (2020). HPLC-DAD-ESI-QTOF-MS Determination of Bioactive Compounds and Antioxidant Activity Comparison of the Hydroalcoholic and Water Extracts from Two *Helichrysum italicum* Species. Metabolites.

[90] Seong E.-H., Gong D.-S., Shiwakoti S., Adhikari D., Kim H.J., Oak M.-H. (2022). Taxifolin as a Major Bioactive Compound in the Vasorelaxant Effect of Different Pigmented Rice Bran Extracts. Front. Pharmacol..

[91] Sharifi-Rad J., Cruz-Martins N., Lopez Jornet P., Pons-Fuster E., Harun N., Yeskaliyeva B., Beyatli A., Sytar O., Shaheen S., Sharopov F. (2021). Natural Coumarins: Exploring the Pharmacological Complexity and Underlying Molecular Mechanisms. Oxidative Med. Cell. Longev..

[92] Ressaissi A., Attia N., Falé P.L., Pacheco R., Victor B.L., Machuqueiro M., Serralheiro M.L. (2017). Isorhamnetin derivatives and piscidic acid for hypercholesterolemia: Cholesterol permeability, HMG-CoA reductase inhibition, and docking studies. Arch. Pharm. Res..

[93] Manzoor F., Nisa M.U., Amjad H., Ahmad N., Umbreen H. (2020). Effect of different levels of hydrolysable tannin intake on the reproductive hormones and serum biochemical indices in healthy female rats. Sci. Rep..

[94] Atia A., Barhoumi Z., Mokded R., Abdelly C., Smaoui A. (2011). Environmental eco-physiology and economical potential of the halophyte *Crithmum maritimum* L. (*Apiaceae*). J. Med. Plants Res..

[95] Alves-Silva J.M., Guerra I., Gonçalves M.J., Cavaleiro C., Cruz M.T., Figueirinha A., Salgueiro L. (2020). Chemical composition of *Crithmum maritimum* L. essential oil and hydrodistillation residual water by GC-MS and HPLC-DAD-MS/MS, and their biological activities. Ind. Crops Prod..

[96] Fontana G., Bruno M., Senatore F., Formisano C. (2014). Volatile constituents of aerial parts of two Mediterranean species of Inula: *Inula crithmoides* L. and *I. verbascifolia* (Willd.) Hausskn. (Asteraceae). Nat. Prod. Res..

[97] D’Agostino G., Badalamenti N., Franco P., Bruno M., Gallo G. (2022). The chemical composition of the flowers essential oil of *Inula crithmoides* (*Asteraceae*) growing in aeolian islands, Sicily (Italy) and its biocide properties on microorganisms affecting historical art crafts. Nat. Prod. Res..

[98] Amanpour A., Selli S. (2016). Differentiation of Volatile Profiles and Odor Activity Values of Turkish Coffee and French Press Coffee. J. Food Process. Preserv..

[99] Aisala H., Sola J., Hopia A., Linderborg K.M., Sandell M. (2019). Odor-contributing volatile compounds of wild edible Nordic mushrooms analyzed with HS–SPME–GC–MS and HS–SPME–GC–O/FID. Food Chem..

[100] Choi H.S. (2005). Characteristic odor components of kumquat (*Fortunella japonica* Swingle) peel oil. J. Agric. Food Chem..

[101] Delort E., Jaquier A., Chapuis C., Rubin M., Starkenmann C. (2012). Volatile composition of oyster leaf (*Mertensia maritima* (L.) Gray). J. Agric. Food Chem..

[102] Hossain M.B., Rai D.K., Brunton N.P., Martin-Diana A.B., Barry-Ryan C. (2010). Characterization of Phenolic Composition in *Lamiaceae* Spices by LC-ESI-MS/MS. J. Agric. Food Chem..

[103] Oliveira-Alves S.C., Vendramini-Costa D.B., Betim Cazarin C.B., Maróstica Júnior M.R., Borges Ferreira J.P., Silva A.B., Prado M.A., Bronze M.R. (2017). Characterization of phenolic compounds in chia (*Salvia hispanica* L.) seeds, fiber flour and oil. Food Chem..

[104] Cádiz-Gurrea M.L., Fernández-Arroyo S., Joven J., Segura-Carretero A. (2013). Comprehensive characterization by UHPLC-ESI-Q-TOF-MS from an *Eryngium bourgatii* extract and their antioxidant and anti-inflammatory activities. Food Res. Int..

[105] Perestrelo R., Lu Y., Santos S.A.O., Silvestre A.J.D., Neto C.P., Câmara J.S., Rocha S.M. (2012). Phenolic profile of Sercial and Tinta Negra *Vitis vinifera* L. grape skins by HPLC–DAD–ESI-MSn: Novel phenolic compounds in *Vitis vinifera* L. grape. Food Chem..

[106] Romani A., Campo M., Pinelli P. (2012). HPLC/DAD/ESI-MS analyses and anti-radical activity of hydrolyzable tannins from different vegetal species. Food Chem..

[107] Lee J.-H., Johnson J.V., Talcott S.T. (2005). Identification of Ellagic Acid Conjugates and Other Polyphenolics in Muscadine Grapes by HPLC-ESI-MS. J. Agric. Food Chem..

[108] Oliveira-Alves S.C., Pereira R.S., Pereira A.B., Ferreira A., Mecha E., Silva A.B., Serra A.T., Bronze M.R. (2020). Identification of functional compounds in baru (*Dipteryx alata* Vog.) nuts: Nutritional value, volatile and phenolic composition, antioxidant activity and antiproliferative effect. Food Res. Int..

[109] Ziani B.E.C., Barros L., Zineddine B.A., Bachari K., Heleno S., Alves M.J., Ferreira I.C.F.R. (2018). Profiling polyphenols composition by HPLC-DAD-ESI/MSn and antibacterial activity of infusion preparations obtained from four medicinal plants. Food Funct..

[110] Kelebek H., Sevindik O., Selli S. (2019). LC-DAD-ESI-MS/MS-based phenolic profiling of St John’s Wort Teas and their antioxidant activity: Eliciting infusion induced changes. J. Liq. Chromatogr. Relat. Technol..

[111] Justesen U. (2000). Negative atmospheric pressure chemical ionisation low-energy collision activation mass spectrometry for the characterisation of flavonoids in extracts of fresh herbs. J. Chromatogr. A.

[112] Kheyar-Kraouche N., da Silva A.B., Serra A.T., Bedjou F., Bronze M.R. (2018). Characterization by liquid chromatography–mass spectrometry and antioxidant activity of an ethanolic extract of *Inula viscosa* leaves. J. Pharm. Biomed. Anal..

[113] Nabet N., Gilbert-López B., Madani K., Herrero M., Ibáñez E., Mendiola J.A. (2019). Optimization of microwave-assisted extraction recovery of bioactive compounds from *Origanum glandulosum* and *Thymus fontanesii*. Ind. Crops Prod..

[114] Sánchez-Salcedo E.M., Tassotti M., Del Rio D., Hernández F., Martínez J.J., Mena P. (2016). (Poly)phenolic fingerprint and chemometric analysis of white (*Morus alba* L.) and black (*Morus nigra* L.) mulberry leaves by using a non-targeted UHPLC-MS approach. Food Chem..

[115] Mäkilä L., Laaksonen O., Alanne A.-L., Kortesniemi M., Kallio H., Yang B. (2016). Stability of Hydroxycinnamic Acid Derivatives, Flavonol Glycosides and Anthocyanins in Black Currant Juice. J. Agric. Food Chem..

[116] Surget G., Stiger-Pouvreau V., Le Lann K., Kervarec N., Couteau C., Coiffard L.J.M., Gaillard F., Cahier K., Guérard F., Poupart N. (2015). Structural elucidation, in vitro antioxidant and photoprotective capacities of a purified polyphenolic-enriched fraction from a saltmarsh plant. J. Photochem. Photobiol. B Biol..

[117] Faustino M.V., Faustino M.A.F., Silva H., Cunha Â., Silva A.M.S., Pinto D.C.G.A. (2019). *Puccinellia maritima*, *Spartina maritime*, and *Spartina patens* Halophytic Grasses: Characterization of Polyphenolic and Chlorophyll Profiles and Evaluation of Their Biological Activities. Molecules.

[118] Falcão S.I., Vale N., Gomes P., Domingues M.R.M., Freire C., Cardoso S.M., Vilas-Boas M. (2013). Phenolic Profiling of Portuguese Propolis by LC-MS Spectrometry: Uncommon Propolis Rich in Flavonoid Glycosides. Phytochem. Anal..

[119] Vallverdú-Queralt A., Jáuregui O., Medina-Remón A., Andrés-Lacueva C., Lamuela-Raventós R.M. (2010). Improved characterization of tomato polyphenols using liquid chromatography/electrospray ionization linear ion trap quadrupole Orbitrap mass spectrometry and liquid chromatography/electrospray ionization tandem mass spectrometry. Rapid Commun. Mass Spectrom..

[120] Sinosaki N.B.M., Tonin A.P.P., Ribeiro M.A.S., Poliseli C.B., Roberto S.B., Silveira R., Visentainer J.V., Santos O.A., Meurer E.C. (2019). Structural Study of Phenolic Acids by Triple Quadrupole Mass Spectrometry with Electrospray Ionization in Negative Mode and H/D Isotopic Exchange. J. Braz. Chem. Soc..

[121] Barros L., Dueñas M., Ferreira I.C.F.R., Carvalho A.M., Santos-Buelga C. (2011). Use of HPLC–DAD–ESI/MS to profile phenolic compounds in edible wild greens from Portugal. Food Chem..

[122] Chandradevan M., Simoh S., Mediani A., Ismail N.H., Ismail I.S., Abas F. (2020). UHPLC-ESI-Orbitrap-MS Analysis of Biologically Active Extracts from *Gynura procumbens* (Lour.) Merr. and *Cleome gynandra* L. Leaves. Evid. Based Complement. Altern. Med..

[123] Abu-Reidah I.M., Arráez-Román D., Segura-Carretero A., Fernández-Gutiérrez A. (2013). Profiling of phenolic and other polar constituents from hydro-methanolic extract of watermelon (*Citrullus lanatus*) by means of accurate-mass spectrometry (HPLC–ESI–QTOF–MS). Food Res. Int..

[124] Simirgiotis M.J., Caligari P.D.S., Schmeda-Hirschmann G. (2009). Identification of phenolic compounds from the fruits of the mountain papaya *Vasconcellea pubescens* A. DC. grown in Chile by liquid chromatography–UV detection–mass spectrometry. Food Chem..

[125] Díaz-de-Cerio E., Verardo V., Gómez-Caravaca A.M., Fernández-Gutiérrez A., Segura-Carretero A. (2015). Determination of Polar Compounds in Guava Leaves Infusions and Ultrasound Aqueous Extract by HPLC-ESI-MS. J. Chem..

[126] Saldanha A.A., de Siqueira J.M., Castro A.H.F., de Azambuja Ribeiro R.I.M., de Oliveira F.M., de Oliveira Lopes D., Pinto F.C.H., Silva D.B., Soares A.C. (2016). Anti-inflammatory effects of the butanolic fraction of *Byrsonima verbascifolia* leaves: Mechanisms involving inhibition of tumor necrosis factor alpha, prostaglandin E2 production and migration of polymorphonuclear leucocyte in vivo experimentation. Int. Immunopharmacol..

[127] Ghareeb M.A., Mohamed T., Saad A.M., Refahy L.A.-G., Sobeh M., Wink M. (2018). HPLC-DAD-ESI-MS/MS analysis of fruits from *Firmiana simplex* (L.) and evaluation of their antioxidant and antigenotoxic properties. J. Pharm. Pharmacol..

[128] M’rabet Y., Rokbeni N., Cluzet S., Boulila A., Richard T., Krisa S., Marzouki L., Casabianca H., Hosni K. (2017). Profiling of phenolic compounds and antioxidant activity of *Melia azedarach* L. leaves and fruits at two stages of maturity. Ind. Crops Prod..

[129] Hussain F., Jahan N., Rahman K., Sultana B., Jamil S. (2018). Identification of Hypotensive Biofunctional Compounds of *Coriandrum sativum* and Evaluation of Their Angiotensin-Converting Enzyme (ACE) Inhibition Potential. Oxidative Med. Cell. Longev..

[130] Nina N., Quispe C., Jiménez-Aspee F., Theoduloz C., Feresín G., Lima B., Leiva E., Schmeda-Hirschmann G. (2015). Antibacterial Activity, Antioxidant Effect and Chemical Composition of Propolis from the Región del Maule, Central Chile. Molecules.

[131] Ye M., Yang W.-Z., Liu K.-D., Qiao X., Li B.-J., Cheng J., Feng J., Guo D.-A., Zhao Y.-Y. (2012). Characterization of flavonoids in *Millettia nitida* var. hirsutissima by HPLC/DAD/ESI-MS^n^. J. Pharm. Anal..

[132] Barros L., Dueñas M., Carvalho A.M., Ferreira I.C.F.R., Santos-Buelga C. (2012). Characterization of phenolic compounds in flowers of wild medicinal plants from Northeastern Portugal. Food Chem. Toxicol..

[133] Ayouaz S., Oliveira-Alves S.C., Lefsih K., Serra A.T., Silva A.B., Samah M., Karczewski J., Madani K., Bronze M.R. (2020). Quercetin and chlorogenic derivatives rich Phenolic content from *Nerium oleander* leaves: Microwave assisted extraction, characterization, antiproliferative and cytotoxic activities. Food Funct..

[134] Ayouaz S., Oliveira-Alves S.C., Serra A.T., Lefsih K., Samah M., Silva A.B., Madani K., Bronze M.R. (2021). LC-DAD-ESI-MS/MS analysis and cytotoxic and antiproliferative effects of chlorogenic acid derivative rich extract from *Nerium oleander* L. pink flowers. Food Funct..

[135] Han J., Ye M., Xu M., Sun J., Wang B., Guo D. (2007). Characterization of flavonoids in the traditional Chinese herbal medicine-Huangqin by liquid chromatography coupled with electrospray ionization mass spectrometry. J. Chromatogr B Anal. Technol Biomed Life Sci..

[136] Can T.H., Tufekci E.F., Altunoglu Y.C., Baloglu M.C., Llorent-Martínez E.J., Stefanucci A., Mollica A., Cichelli A., Zengin G. (2020). Chemical characterization, computational analysis and biological views on *Daphne gnidioides* Jaub. & Spach extracts: Can a new raw material be provided for biopharmaceutical applications?. Comput. Biol. Chem..

[137] Barreca D., Bellocco E., Caristi C., Leuzzi U., Gattuso G. (2011). Kumquat (*Fortunella japonica* Swingle) juice: Flavonoid distribution and antioxidant properties. Food Res. Int..

[138] Lin L.-Z., Mukhopadhyay S., Robbins R.J., James M., Harnly J.M. (2007). Identification and quantification of flavonoids of Mexican oregano (*Lippia graveolens*) by LC-DAD-ESI/MS analysis. Food Compost. Anal..

[139] Wang J., Liu S., Li S., Song F., Zhang Y., Liu Z., Liu C. (2014). Ultrafiltration LC-PDA-ESI/MS combined with reverse phase-medium pressure liquid chromatography for screening and isolation potential α-glucosidase inhibitors from *Scutellaria baicalensis* Georgi. Anal. Methods.

[140] Tarola A.M., Van de Velde F., Salvagni L., Preti R. (2013). Determination of Phenolic Compounds in Strawberries (*Fragaria ananassa* Duch) by High Performance Liquid Chromatography with Diode Array Detection. Food Anal. Methods.

[141] Kammerer D., Claus A., Carle R., Schieber A. (2004). Polyphenol Screening of Pomace from Red and White Grape Varieties (*Vitis vinifera* L.) by HPLC-DAD-MS/MS. J. Agric. Food Chem..

[142] Mata A., Ferreira J.P., Semedo C., Serra T., Duarte C.M.M., Bronze M.R. (2016). Contribution to the characterization of *Opuntia* spp. juices by LC-DAD-ESI-MS/MS. Food Chem..

[143] Engels C., Gräter D., Esquivel P., Jiménez V.M., Gänzle M.G., Schieber A. (2012). Characterization of phenolic compounds in jocote (*Spondias purpurea* L.) peels by ultra high-performance liquid chromatography/electrospray ionization mass spectrometry. Food Res. Int..

[144] Graça V.C., Dias M.I., Barros L., Calhelha R.C., Santos P.F., Ferreira I.C.F.R. (2018). Fractionation of the more active extracts of *Geranium molle* L.: A relationship between phenolic profile and biological activity. Food Funct..

[145] Wyrepkowski C.C., Gomes da Costa D.L.M., Sinhorin A.P., Vilegas W., De Grandis R.A., Resende F.A., Varanda E.A., Dos Santos L.C. (2014). Characterization and Quantification of the Compounds of the Ethanolic Extract from *Caesalpinia ferrea* Stem Bark and Evaluation of Their Mutagenic Activity. Molecules.

[146] Aouadi M., Escribano-Bailón M.T., Guenni S.H., Hannachi A.S., Montserrat D. (2019). Qualitative and quantitative analyses of phenolic compounds by HPLC–DAD–ESI/MS in *Tunisian Pistacia vera* L. Leaves unveiled a rich source of phenolic compounds with a significant antioxidant potential. Food Meas..

[147] Grochowski D.M., Uysal S., Aktumsek A., Granica S., Zengin G., Ceylan R., Locatelli M., Tomczyk M. (2017). In vitro enzyme inhibitory properties, antioxidant activities, and phytochemical profile of *Potentilla thuringiaca*. Phytochem. Lett..

[148] De Leo M., De Abreu M.B., Pawlowska A.M., Cioni P.L., Braca A. (2010). Profiling the chemical content of *Opuntia ficus-indica* flowers by HPLC–PDA-ESI-MS and GC/EIMS analyses. Phytochem. Lett..

[149] Spínola V., Pinto J., Castilho P.C. (2015). Identification and quantification of phenolic compounds of selected fruits from Madeira Island by HPLC-DAD–ESI-MS^n^ and screening for their antioxidant activity. Food Chem..

[150] Mena P., Calani L., Dall’Asta C., Galaverna G., García-Viguera C., Bruni R., Crozier A., Del Rio D. (2012). Rapid and Comprehensive Evaluation of (Poly)phenolic Compounds in Pomegranate (*Punica granatum* L.) Juice by UHPLC-MSn. Molecules.

[151] Ding S., Dudley E., Plummer S., Tang J., Newton R.P., Brenton A.G. (2008). Fingerprint profile of *Ginkgo biloba* nutritional supplements by LC/ESI-MS/MS. Phytochemical.

[152] Gouveia S., Castilho P.C. (2011). Characterisation of phenolic acid derivatives and flavonoids from different morphological parts of *Helichrysum obconicum* by a RP-HPLC–DAD-(−)–ESI-MS^n^ method. Food Chem..

[153] Lin L., Zhuang M., Lei F., Yang B., Zhao M. (2013). GC/MS analysis of volatiles obtained by headspace solid-phase microextraction and simultaneous–distillation extraction from *Rabdosia serra* (MAXIM.) HARA leaf and stem. Food Chem..

[154] Radulović N., Blagojević P., Palić R. (2010). Comparative study of the leaf volatiles of *Arctostaphylos uva-ursi* (L.) Spreng. and *Vaccinium vitis-idaea* L. (Ericaceae). Molecules.

[155] Aparicio R., Morales M.T. (1998). Characterization of Olive Ripeness by Green Aroma Compounds of Virgin Olive Oil. J. Agric. Food Chem..

[156] Costa R., d’Acampora Zellner B., Crupi M.L., Fina M.R.D., Valentino M.R., Dugo P., Dugo G., Mondello L. (2008). GC–MS, GC–O and enantio–GC investigation of the essential oil of *Tarchonanthus camphoratus* L.. Flavour Fragr. J..

[157] Jirovetz L., Smith D., Buchbauer G. (2002). Aroma Compound Analysis of *Eruca sativa* (Brassicaceae) SPME Headspace Leaf Samples Using GC, GC−MS, and Olfactometry. J. Agric. Food Chem..

[158] Aligiannis N., Kalpoutzakis E., Mitaku S., Chinou I.B. (2001). Composition and antimicrobial activity of the essential oils of two *Origanum* species. J. Agric. Food Chem..

[159] Hazzit M., Baaliouamer A., Faleiro M.L., Graça M.M. (2006). Composition of the essential oils of *Thymus* and *Origanum* species from Algeria and their antioxidant and antimicrobial activities. J. Agric. Food Chem..

[160] Stashenko E.E., Martínez J.R., Ruíz C.A., Arias G., Durán C., Salgar W., Cala M. (2010). *Lippia origanoides* chemotype differentiation based on essential oil GC-MS and principal component analysis. J. Sep. Sci..

[161] Karabagias I.K., Karabagias V.K., Riganakos K.A. (2019). Physico-Chemical Parameters, Phenolic Profile, In Vitro Antioxidant Activity and Volatile Compounds of Ladastacho (*Lavandula stoechas*) from the Region of Saidona. Antioxidants.

[162] Gerretzen J., Buydens L.M.C., Tromp-van den Beukel A.O., Koussissi E., Brouwer E.R., Jansen J.J., Szymańska E. (2015). A novel approach for analyzing gas chromatography-mass spectrometry/olfactometry data. Chemom. Intell. Lab. Syst..

[163] Nguir A., Besbes M., Ben Jannet H., Flamini G., harzallah-Skhiri F., Hamza M.H. (2011). Chemical Composition, Antioxidant and Anti-acetylcholinesterase activities of Tunisian *Crithmum maritimum* L. Essential oils. Mediterr. J. Chem..

[164] Minh Tu N.T., Onishi Y., Choi H.-S., Kondo Y., Bassore S.M., Ukeda H., Sawamura M. (2002). Characteristic Odor Components of *Citrus sphaerocarpa* Tanaka (Kabosu) Cold-Pressed Peel Oil. J. Agric. Food Chem..

[165] Vasta V., D’Alessandro A.G., Priolo A., Petrotos K., Martemucci G. (2012). Volatile compound profile of ewe’s milk and meat of their suckling lambs in relation to pasture vs. indoor feeding system. Small Rumin. Res..

[166] Yi L., Dong N., Liu S., Yi Z., Zhang Y. (2015). Chemical features of *Pericarpium Citri Reticulatae* and *Pericarpium Citri Reticulatae Viride* revealed by GC–MS metabolomics analysis. Food Chem..

[167] Pino J.A., Quijano C.E. (2012). Estudo de compostos voláteis de ameixa (*prunus domestica* L. cv. horvin) e estimativa da sua contribuição ao aroma. Cienc. Tecnol. Aliment..

[168] Figuérédo G., Chalchat J.-C., Petrovic S., Maksimovic Z., Gorunovic M., Boza P., Radic J. (2009). Composition of Essential Oils of Flowers, Leaves, Stems and Rhizome of *Peucedanum officinale* L. (Apiaceae). J. Essent. Oil Res..

[169] Pino J.A., Marbot R., Vázquez C. (2001). Characterization of Volatiles in Strawberry Guava (*Psidium cattleianum* Sabine) Fruit. J. Agric. Food Chem..

[170] Wang Y., Finn C., Qian M.C. (2005). Impact of growing environment on Chickasaw blackberry (*Rubus* L) aroma evaluated by gas chromatography olfactometry dilution analysis. J. Agric. Food Chem..

[171] Flamini G., Tebano M., Cioni P.L. (2007). Volatiles emission patterns of different plant organs and pollen of *Citrus limon*. Anal. Chim. Acta.

[172] Cai X., Mai R.-Z., Zou J.-J., Zhang H.-Y., Zeng X.-L., Zheng R.-R., Wang C.-Y. (2014). Analysis of aroma-active compounds in three sweet osmanthus (*Osmanthus fragrans*) cultivars by GC-olfactometry and GC-MS. J. Zhejiang Univ.-Sci. B.

[173] Xie J., Sun B., Zheng F., Wang S. (2008). Volatile flavor constituents in roasted pork of Mini-pig. Food Chem..

[174] Mustapha M.B., Zardi-Bergaoui A., Chaieb I., Flamini G., Ascrizzi R., Jannet H.B. (2020). Chemical Composition and Insecticidal Activity of *Crithmum maritimum* L. Essential Oil against Stored-Product Beetle *Tribolium Castaneum*. Chem. Biodivers..

[175] Flamini G., Cioni P.L., Morelli I. (2002). Differences in the Fragrances of Pollen and Different Floral Parts of Male and Female Flowers of *Laurus nobilis*. J. Agric. Food Chem..

[176] Hudaib M., Speroni E., Di Pietra A.M., Cavrini V. (2002). GC/MS evaluation of thyme (*Thymus vulgaris* L.) oil composition and variations during the vegetative cycle. J. Pharm. Biomed. Anal..

[177] Schreiner L., Bauer J., Ortner E., Buettner A. (2020). Structure–Odor Activity Studies on Derivatives of Aromatic and Oxygenated Monoterpenoids Synthesized by Modifying p-Cymene. J. Nat. Prod..

[178] Laokuldilok N., Utama-ang N., Kopermsub P., Thakeow P. (2017). Characterization of Odor Active Compounds of Fresh and Dried Turmeric by Gas Chromatography-Mass Spectrometry, Gas Chromatography Olfactometry and Sensory Evaluation. FAB J..

[179] Stoppacher N., Kluger B., Zeilinger S., Krska R., Schuhmacher R. (2010). Identification and profiling of volatile metabolites of the biocontrol fungus *Trichoderma atroviride* by HS-SPME-GC-MS. J. Microbiol. Methods.

[180] Sampaio T.S., Nogueira P.C.L. (2006). Volatile components of mangaba fruit (*Hancornia speciosa* Gomes) at three stages of maturity. Food Chem..

[181] Quijano C.E., Linares D., Pino J.A. (2007). Changes in volatile compounds of fermented cereza agria [*Phyllanthus acidus* (L.) Skeels] fruit. Flavour Fragr. J..

[182] Lykomitros D., Fogliano V., Capuano E. (2016). Flavor of roasted peanuts (*Arachis hypogaea*)—Part II: Correlation of volatile compounds to sensory characteristics. Food Res. Int..

[183] Telci I., Demirtas I., Sahin A. (2009). Variation in plant properties and essential oil composition of sweet fennel (*Foeniculum vulgare* Mill.) fruits during stages of maturity. Ind. Crops Prod..

[184] Jordán M.J., Tandon K., Shaw P.E., Goodner K.L. (2001). Aromatic Profile of Aqueous Banana Essence and Banana Fruit by Gas Chromatography−Mass Spectrometry (GC-MS) and Gas Chromatography−Olfactometry (GC-O). J. Agric. Food Chem..

[185] Gürbüz O., Rouseff J.M., Rouseff R.L. (2006). Comparison of Aroma Volatiles in Commercial Merlot and Cabernet Sauvignon Wines Using Gas Chromatography−Olfactometry and Gas Chromatography−Mass Spectrometry. J. Agric. Food Chem..

[186] Cardeal Z.L., Gomes da Silva M.D., Marriott P.J. (2006). Comprehensive two-dimensional gas chromatography/mass spectrometric analysis of pepper volatiles. Rapid Commun. Mass Spectrom..

[187] Beaulieu J.C., Grimm C.C. (2001). Identification of Volatile Compounds in Cantaloupe at Various Developmental Stages Using Solid Phase Microextraction. J. Agric. Food Chem..

[188] Varlet V., Knockaert C., Prost C., Serot T. (2006). Comparison of Odor-Active Volatile Compounds of Fresh and Smoked Salmon. J. Agric. Food Chem..

[189] Purcaro G., Tranchida P.Q., Jacques R.A., Caramão E.B., Moret S., Conte L., Dugo P., Dugo G., Mondello L. (2009). Characterization of the yerba mate (*Ilex paraguariensis*) volatile fraction using solid-phase microextraction-comprehensive 2-D GC-MS. J. Sep. Sci..

[190] Jirovetz L., Buchbauer G., Denkova Z., Slavchev A., Stoyanova A., Schmidt E. (2006). Chemical composition, antimicrobial activities and odor descriptions of various *Salvia* sp. and *Thuja* sp. essential oils. Nutrition-Vienna.

[191] Nezhadali A., Shirvan B.Z. (2010). Separation, Identification and Determination of Volatile Compounds of *Ziziphora persica* Bunge Using HS-SPME/GC-MS. Int. J. Environ. Sci. Dev..

[192] Niu Y., Wang P., Xiao Q., Xiao Z., Mao H., Zhang J. (2020). Characterization of Odor-Active Volatiles and Odor Contribution Based on Binary Interaction Effects in Mango and Vodka Cocktail. Molecules.

[193] Jeribi C., Karoui I.J., Benhassine D., Abderrabba M. (2016). Chemical Composition of *Cardopatium corymbosum* Leaves Essential Oil. J. Essent. Oil Bear. Plants.

[194] Villa-Ruano N., Pacheco-Hernández Y., Cruz-Durán R., Lozoya-Gloria E. (2015). Volatiles and seasonal variation of the essential oil composition from the leaves of *Clinopodium macrostemum* var. *laevigatum* and its biological activities. Ind. Crops Prod..

[195] Mockute D., Bernotiene G. (1999). The Main Citral−Geraniol and Carvacrol Chemotypes of the Essential Oil of *Thymus pulegioides* L. Growing Wild in Vilnius District (Lithuania). J. Agric. Food Chem..

[196] Moon S.-Y., Cliff M.A., Li-Chan E.C.Y. (2006). Odour-active components of simulated beef flavour analysed by solid phase microextraction and gas chromatography–mass spectrometry and –olfactometry. Food Res. Int..

[197] Giorgi A., Panseri S., Nanayakkara N.N.M.C., Chiesa L. (2012). HS-SPME-GC/MS analysis of the volatile compounds of *Achillea collina*: Evaluation of the emissions fingerprint induced by *Myzus persicae* infestation. J. Plant Biol..

[198] Kokoska L., Urbanova K., Kloucek P., Nedorostova L., Polesna L., Malik J., Jiros P., Havlik J., Vadlejch J., Valterova I. (2012). Essential Oils in the Ranunculaceae Family: Chemical Composition of Hydrodistilled Oils from *Consolida regalis*, *Delphinium elatum*, *Nigella hispanica*, and *N. nigellastrum* Seeds. Chem. Biodivers..

[199] Cho I.H., Lee S.M., Kim S.Y., Choi H., Kim K.-O., Kim Y.-S. (2007). Differentiation of aroma characteristics of pine-mushrooms (*Tricholoma matsutake* Sing.) of different grades using gas chromatography−olfactometry and sensory analysis. J. Agric. Food Chem..

[200] Gong W.-c., Chen G., Liu C.-q., Dunn B.L., Sun W.-b. (2014). Comparison of floral scent between and within *Buddleja fallowiana* and *Buddleja officinalis* (*Scrophulariaceae*). Biochem. Syst. Ecol..

[201] Ma R., Liu X., Tian H., Han B., Li Y., Tang C., Zhu K., Li C., Meng Y. (2020). Odor-active volatile compounds profile of triploid rainbow trout with different marketable sizes. Aquac. Rep..

[202] Wang P., Ma X., Wang W., Xu D., Zhang X., Zhang J., Sun Y. (2019). Characterization of flavor fingerprinting of red sufu during fermentation and the comparison of volatiles of typical products. Food Sci. Hum. Wellness.

[203] Tontul I., Torun M., Dincer C., Sahin-Nadeem H., Topuz A., Turna T., Ozdemir F. (2013). Comparative study on volatile compounds in Turkish green tea powder: Impact of tea clone, shading level and shooting period. Food Res. Int..

[204] Villberg K., Veijanen A., Gustafsson I., Wickström K. (1997). Analysis of odour and taste problems in high-density polyethene. J. Chromatogr. A.

[205] Guo X., Ho C.-T., Wan X., Zhu H., Liu Q., Wen Z. (2021). Changes of volatile compounds and odor profiles in Wuyi rock tea during processing. Food Chem..

[206] Villa C., Trucchi B., Bertoli A., Pistelli L., Parodi A., Bassi A.M., Ruffoni B. (2009). *Salvia somalensis* essential oil as a potential cosmetic ingredient: Solvent-free microwave extraction, hydrodistillation, GC–MS analysis, odour evaluation and in vitro cytotoxicity assays. Int. J. Cosmet. Sci..

[207] Liu J., Tang X., Zhang Y., Zhao W. (2012). Determination of the Volatile Composition in Brown Millet, Milled Millet and Millet Bran by Gas Chromatography/Mass Spectrometry. Molecules.

[208] Zhu J., Chen F., Wang L., Niu Y., Yu D., Shu C., Chen H., Wang H., Xiao Z. (2015). Comparison of Aroma-Active Volatiles in Oolong Tea Infusions Using GC–Olfactometry, GC–FPD, and GC–MS. J. Agric. Food Chem..

[209] Huang X.-H., Zheng X., Chen Z.-H., Zhang Y.-Y., Du M., Dong X.-P., Qin L., Zhu B.-W. (2019). Fresh and grilled eel volatile fingerprinting by e-Nose, GC-O, GC–MS and GC × GC-QTOF combined with purge and trap and solvent-assisted flavor evaporation. Food Res. Int..

[210] Angioni A., Barra A., Coroneo V., Dessi S., Cabras P. (2006). Chemical composition, seasonal variability, and antifungal activity of *Lavandula stoechas* L. ssp. *stoechas* Essential Oils from Stem/Leaves and Flowers. J. Agric. Food Chem..

[211] Lu Q., Liu F., Bao J. (2019). Volatile components of *American silver* carp analyzed by electronic nose and MMSE-GC-MS-O. J. Food Biochem..

[212] Kishimoto N., Kashiwagi A. Prediction of Specific Odor Markers in Oil from Olive Fruit Infested with Olive Scale Using an Electronic Nose. Proceedings of the 2019 IEEE International Symposium on Olfaction and Electronic Nose (ISOEN).

[213] Kiatbenjakul P., Intarapichet K.-O., Cadwallader K.R. (2015). Characterization of potent odorants in male giant water bug (*Lethocerus indicus* Lep. and Serv.), an important edible insect of Southeast Asia. Food Chem..

[214] Mahattanatawee K., Luanphaisarnnont T., Rouseff R. (2018). Comparison of Aroma Character Impact Volatiles of Thummong Leaves (*Litsea petiolata* Hook. f.), Mangdana Water Beetle (*Lethocerus indicus*), and a Commercial Product as Flavoring Agents in Thai Traditional Cooking. J. Agric. Food Chem..

[215] Pino J.A., Mesa J., Muñoz Y., Martí M.P., Marbot R. (2005). Volatile components from mango (*Mangifera indica* L.) cultivars. J. Agric. Food Chem..

[216] Nóbrega I.C.C., Ataíde C.S., Moura O.M., Livera A.V., Menezes P.H. (2007). Volatile constituents of cooked bullfrog (*Rana catesbeiana*) legs. Food Chem..

[217] Cuevas F.J., Moreno-Rojas J.M., Ruiz-Moreno M.J. (2017). Assessing a traceability technique in fresh oranges (*Citrus sinensis* L. Osbeck) with an HS-SPME-GC-MS method. Towards a volatile characterisation of organic oranges. Food Chem..

[218] Baião L.F., Oliveira A.S., Gonçalves A., Guedes de Pinho P., Valente L.M.P., Cunha L.M. (2020). Analysis of volatile compounds in *Paracentrotus lividus* by HS-SPME/GS-MS and relation to its sensorial properties. LWT.

[219] Bianchi G., Falcinelli B., Tosti G., Bocci L., Benincasa P. (2019). Taste quality traits and volatile profiles of sprouts and wheatgrass from hulled and non-hulled *Triticum* species. J. Food Biochem..

[220] Yang D.S., Shewfelt R.L., Lee K.-S., Kays S.J. (2008). Comparison of Odor-Active Compounds from Six Distinctly Different Rice Flavor Types. J. Agric. Food Chem..

[221] Cai L., Ao Z., Tang T., Tong F., Wei Z., Yang F., Shu Y., Liu S., Mai K. (2021). Characterization of difference in muscle volatile compounds between triploid and diploid crucian carp. Aquac. Rep..

[222] Zhang K., Lin T.F., Zhang T., Li C., Gao N. (2013). Characterization of typical taste and odor compounds formed by *Microcystis Aeruginosa*. J. Environ. Sci..

[223] Li Q., Shi X., Zhao Q., Cui Y., Ouyang J., Xu F. (2016). Effect of cooking methods on nutritional quality and volatile compounds of Chinese chestnut (*Castanea mollissima* Blume). Food Chem..

[224] Yang C., Wang Y., Liang Z., Fan P., Wu B., Yang L., Wang Y., Li S. (2009). Volatiles of grape berries evaluated at the germplasm level by headspace-SPME with GC–MS. Food Chem..

[225] Chen J.L., Wu J.H., Wang Q., Deng H., Hu X.S. (2006). Changes in the Volatile Compounds and Chemical and Physical Properties of Kuerle Fragrant Pear (*Pyrus serotina* Reld) during Storage. J. Agric. Food Chem..

[226] Radulović N., Stojanović G., Milovanovic V., Dokovic D., Randjelovic V. (2008). Volatile constituents of *Equisetum fluviatile* L.. J. Essent. Oil Res..

[227] Velasco-Negueruela A., Pérez-Alonso M.J., de Paz P.L.P., Palá-Paúl J., Sanz J. (2005). Analysis by gas chromatography–mass spectrometry of the essential oils from the aerial parts of *Pimpinella anagodendron* Bolle and *Pimpinella rupicola* Svent., two endemic species to the Canary Islands, Spain. J. Chromatogr. A.

[228] García-Aguilar L., Rojas-Molina A., Ibarra-Alvarado C., Rojas-Molina J.I., Vázquez-Landaverde P.A., Luna-Vázquez F.J., Zavala-Sánchez M.A. (2015). Nutritional Value and Volatile Compounds of Black Cherry (*Prunus serotina*) Seeds. Molecules.

[229] Costa R., Tedone L., De Grazia S., Dugo P., Mondello L. (2013). Multiple headspace-solid-phase microextraction: An application to quantification of mushroom volatiles. Anal. Chim. Acta.

[230] Pino J.A., Trujillo R. (2021). Characterization of odour-active compounds of sour guava (*Psidium acidum*[DC.]*Landrum*) fruit by gas chromatography-olfactometry and odour activity value. Flavour Fragr. J..

[231] Ali S.B., Ghatak B., Gupta S.D., Debabhuti N., Chakraborty P., Sharma P., Ghosh A., Tudu B., Mitra S., Sarkar M.P. (2016). Detection of 3-Carene in mango using a quartz crystal microbalance sensor. Sens. Actuators B Chem..

[232] Selli S., Cayhan G.G. (2009). Analysis of volatile compounds of wild gilthead sea bream (*Sparus aurata*) by simultaneous distillation–extraction (SDE) and GC–MS. Microchem. J..

[233] Villière A., Arvisenet G., Lethuaut L., Prost C., Sérot T. (2012). Selection of a representative extraction method for the analysis of odourant volatile composition of French cider by GC–MS–O and GC×GC–TOF-MS. Food Chem..

[234] Shen X., Chen W., Zheng Y., Lei X., Tang M., Wang H., Song F. (2017). Chemical composition, antibacterial and antioxidant activities of hydrosols from different parts of *Areca catechu* L. and *Cocos nucifera* L.. Ind. Crops Prod..

[235] Dussort P., Deprêtre N., Bou-Maroun E., Fant C., Guichard E., Brunerie P., Le Fur Y., Le Quéré J.L. (2012). An original approach for gas chromatography-olfactometry detection frequency analysis: Application to gin. Food Res. Int..

[236] Miranda R.F., de Paula M.M., da Costa G.M., Barão C.E., da Silva A.C.R., Raices R.S.L., Gomes R.G., Pimentel T.C. (2019). Orange juice added with *L. casei*: Is there an impact of the probiotic addition methodology on the quality parameters?. LWT.

[237] Amanpour A., Kelebek H., Kesen S., Selli S. (2016). Characterization of Aroma-Active Compounds in Iranian cv. Mari Olive Oil by Aroma Extract Dilution Analysis and GC–MS-Olfactometry. J. Am. Oil Chem. Soc..

[238] Evstatieva L., Todorova M., Antonova D., Staneva J. (2010). Chemical composition of the essential oils of *Rhodiola rosea* L. of three different origins. Pharm. Mag..

[239] Fu M., Shen X., Peng H., Zhou Q., Yun J., Sun Y., Ho C.-T., Cai H., Hou R. (2020). Identification of rancidity markers in roasted sunflower seeds produced from raw materials stored for different periods of time. LWT.

[240] Matsuishi M., Kume J., Itou Y., Takahashi M., Arai M., Nagatomi H., Watanabe K., Hayase F., Okitani A. (2004). Aroma components of Wagyu beef and imported beef. Nihon Chikusan Gakkaiho.

