# Supplementary material for: Soilless Cultivated Halophyte Plants: Volatile, Nutritional, Phytochemical, and Biological Differences
Source: Antioxidants (Basel). 2023 May 26;12(6):1161. doi: 10.3390/antiox12061161 (PMC10295272; doi:10.3390/antiox12061161)
Supplement: Supplementary file 1 [file antioxidants-12-01161-s001.zip › antioxidants-2387483-supplementary.pdf]

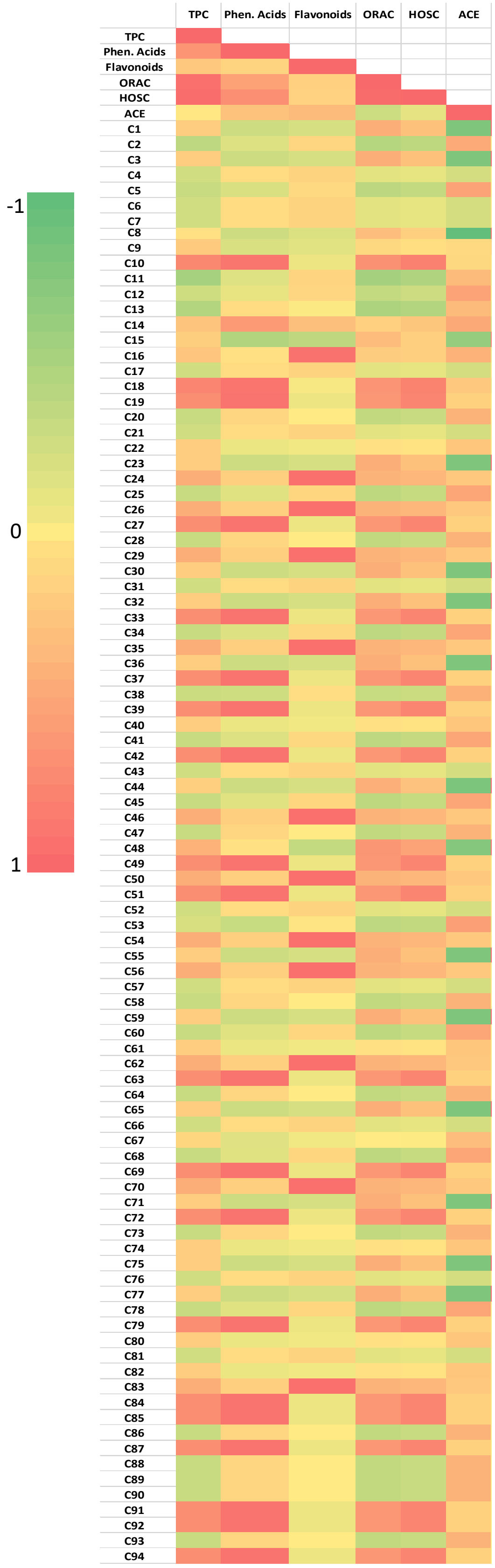

Figure S1. Correlation matrix (Pearson's R values from -1 to 1) between the phenolic composition and the bioactivity measured by *in vitro* antioxidant and anti-hypertensive activities. ACE inhibition expressed as inverse values for the anti-hypertensive activity.

Table S1. Individual phenolic compounds quantified in the different halophyte species. Results expressed as mean ± standard deviation (n = 2), μg/g of fresh weight (FW). The hydroxycinnamic acids (HCA) and their derivatives were quantified as a 3-O-caffeoylquinic acid equivalent (CQAE), flavonoids were quantified as quercetin-3-glucoside equivalent (QGE) and hydroxybenzoic acids (HBA) were quantified as a gallic acid equivalent (GAE). * Phenolic compound identified with standard.

Class	Peak	Phenolic Compounds μg/g FW	*I. crithmoides*	*S. fruticosa*	*S. ramosissima*	*D. crassifolium*	*C. maritimum*	*M. nodiflorum*	*M. crystallinum*
HCA	1	*p*-Coumaric acid derivative	ND	0.370 ± 0.003	ND	ND	ND	ND	ND
	5	5-O-Caffeoylquinic acid	ND	0.680 ± 0.003	4.253 ± 0.033	ND	ND	ND	ND
	8	Caffeic acid derivative	0.482 ± 0.004	1.138 ± 0.028	ND	ND	ND	ND	ND
	11	Caffeic acid-O-glucoside	0.356 ± 0.001	ND	ND	ND	0.744 ± 0.028	ND	ND
	12	*p*-Coumaric acid-O-glucoside	ND	ND	ND	ND	10.495 ± 0.127	0.482 ± 0.008	0.247 ± 0.095
	13	*p*-Coumaric acid	2.868 ± 0.264	ND	1.538 ± 0.003	10.457 ± 1.670	ND	ND	ND
	15	3-O-Caffeoylquinic acid*	2.494 ± 0.326	13.661 ± 0.115	9.543 ± 0.049	ND	47.754 ± 0.141	ND	ND
	18	Ferulic acid-glucoside	ND	ND	0.839 ± 0.001	0.208 ± 0.057	ND	3.912 ± 0.014	0.498 ± 0.062
	19	Ferulic acid derivative	ND	ND	ND	ND	ND	0.408 ± 0.003	ND
	20	Caffeic acid glucuronide	ND	ND	ND	0.302 ± 0.006	ND	ND	ND
	21	Caffeic acid derivative	0.240 ± 0.033	ND	ND	ND	ND	ND	ND
	22	Ferulic acid	ND	ND	1.904 ± 0.011	ND	ND	ND	ND
	23	*p*-Coumaric acid derivative	ND	2.528 ± 0.042	ND	ND	ND	ND	ND
	24	Ferulic acid derivative	ND	ND	ND	ND	ND	ND	0.774 ± 0.073
	26	Sinapic acid -glucoside	ND	ND	ND	ND	ND	ND	3.354 ± 0.356
	29	Feruloylglucaric acid	ND	ND	ND	ND	ND	ND	0.509 ± 0.064
	30	Caffeoylquinic acid derivative	ND	2.720 ± 0.195	ND	ND	ND	ND	ND
	32	Caffeic acid derivative	ND	0.283 ± 0.106	ND	ND	ND	ND	ND
	34	*p*-Coumaric acid derivative	ND	-	0.175 ± 0.001	ND	4.536 ± 0.141	ND	ND
	38	*p*-Coumaroylquinic acid (isomer 1)	ND	11.764 ± 0.200	3.644 ± 0.003	ND	78.310 ± 0.141	ND	0.623 ± 0.078

Table S1. *Cont.*

Class	Peak	Phenolic Compounds	*I. crithmoides*	*S. fruticosa*	*S. ramosissima*	*D. crassifolium*	*C. maritimum*	*M. nodiflorum*	*M. crystallinum*
HCA	39	*p*-Coumaric acid derivative	ND	ND	ND	ND	ND	0.694 ± 0.003	ND
	40	Ferulic acid derivative	ND	ND	0.496 ± 0.014	ND	ND	ND	ND
	41	*p*-Coumaroylquinic acid (isomer 2)	ND	ND	1.307 ± 0.304	ND	43.242 ± 0.283	ND	ND
	43	Feruloylquinic acid	1.623 ± 0.285	ND	ND	ND	ND	ND	ND
	44	3-O-*p*-Coumaroyl-5-O-caffeoylquinic acid	0.132 ± 0.007	3.240 ± 0.082	ND	ND	ND	ND	ND
	47	*p*-Coumaric acid derivative	ND	ND	ND	0.991 ± 0.010	ND	ND	ND
	54	*p*-Coumaric acid derivative	ND	ND	ND	ND	ND	ND	14.490 ± 0.020
	56	*p*-Coumaric acid derivative	ND	ND	ND	ND	ND	ND	17.150 ± 0.184
	61	3,4-Dicaffeoylquinic acid	ND	ND	25.724 ± 0.368	ND	ND	ND	ND
	62	*p*-Coumaric acid glucoside derivative	ND	ND	ND	ND	ND	ND	0.141 ± 0.010
	66	*p*-Coumaric acid derivative	1.120 ± 0.032	ND	ND	ND	ND	ND	ND
	67	3,5-Dicaffeoylquinic acid	ND	ND	31.440 ± 0.557	ND	5.560 ± 0.085	ND	ND
	71	*p*-Coumaric acid derivative	ND	1.985 ± 0.205	ND	ND	ND	ND	ND
	74	4,5-Dicaffeoylquinic acid	ND	ND	18.938 ± 0.945	ND	ND	ND	ND
	75	*p*-Coumaric acid derivative	ND	1.145 ± 0.055	ND	ND	ND	ND	ND
	77	*p*-Coumaric acid derivative	ND	0.624 ± 0.065	ND	ND	ND	ND	ND
	78	*p*-Coumaric acid derivative	ND	ND	ND	ND	1.228 ± 0.057	ND	ND
	80	Caffeoylhydrocaffeoylquinic acid	ND	ND	2.661 ± 0.013	ND	ND	ND	ND
	81	Caffeic acid-glucuronide-glucoside (isomer 2)	0.163 ± 0.008	ND	ND	ND	ND	ND	ND
	84	Caffeoylquinic acid derivative	ND	ND	ND	ND	ND	0.387 ± 0.001	ND
	86	Malonyl-3,4-O-dicaffeoylquinic acid derivative	ND	ND	ND	0.814 ± 0.127	ND	ND	ND
	88	*p*-Coumaric acid derivative	ND	ND	ND	7.747 ± 0.242	ND	ND	ND
	89	Ferulic acid derivative	ND	ND	ND	8.577 ± 0.129	ND	ND	ND
	90	Ferulic acid derivative	ND	ND	ND	0.801 ± 0.208	ND	ND	ND
	93	*p*-Coumaric acid derivative	ND	ND	ND	0.829 ± 0.131	ND	ND	ND
	94	3,5-Diferuoylquinic acid	ND	ND	ND	ND	ND	0.167 ± 0.001	ND

Table S1. *Cont.*

Class	Peak	Phenolic Compounds	*I. crithmoides*	*S. fruticosa*	*S. ramosissima*	*D. crassifolium*	*C. maritimum*	*M. nodiflorum*	*M. crystallinum*
		Σ **HCA**	9.478 ± 0.960[e]	40.138 ± 1.099[c]	102.462 ± 2.302[b]	30.726 ± 2.058[d]	191.869 ± 1.003[a]	6.050 ± 0.03[e]	37.786 ± 0.942[c]
HBA	2	Protocatechuic acid-glucoside	0.179 ± 0.005	ND	ND	ND	1.508 ± 0.048	ND	ND
	3	5-Galloylquinic acid	ND	0.269 ± 0.075	ND	ND	ND	ND	ND
	4	Gallic acid derivative	0.429 ± 0.032	ND	ND	ND	ND	ND	ND
	6	Syringic acid	0.538 ± 0.013	ND	ND	ND	ND	ND	ND
	9	Protocatechuic-acid-arabinoside	ND	1.972 ± 0.229	9.609 ± 0.202	ND	ND	ND	ND
	17	Gallic acid derivative	0.222 ± 0.009	ND	ND	ND	ND	ND	ND
	37	Syringic acid derivative	ND	ND	ND	ND	ND	0.179 ± 0.001	ND
	63	Digalloyl quinic acid rhamnoside	ND	ND	ND	ND	ND	0.253 ± 0.001	ND
	85	Galloylquinic acid derivative	ND	ND	ND	ND	ND	0.163 ± 0.001	ND
	91	Galloylquinic acid derivative	ND	ND	ND	ND	ND	0.379 ± 0.001	ND
		Σ **HBA**	1.368 ± 0.059[c]	2.241 ± 0.304[b]	9.609 ± 0.202[a]	ND	1.508 ± 0.048[c]	0.974 ± 0.004[c]	ND
		Σ **Phenolic acids**	10.846 ± 0.078[f]	42.379 ± 0.092[c]	112.071 ± 0.178[b]	30.726 ± 0.275[e]	193.377 ± 0.112[a]	7.024 ± 0.004[g]	37.786 ± 0.109[d]
Flavone	25	Apigenin 6-C-glucoside-7-O-glucoside	ND	ND	ND	ND	8.688 ± 0.113	ND	ND
	42	Chrysin-6-C-ara-8-C-glu	ND	ND	ND	ND	ND	0.643 ± 0.007	ND
	46	Acacetin 3,6-di-C-glucoside	ND	ND	ND	ND	ND	ND	0.449 ± 0.038
	50	Chrysin-6-C-glucosyl-8-C-arabinoside	ND	ND	ND	ND	ND	ND	7.357 ± 0.480
	60	Apigenin 6-C-glucoside	ND	ND	ND	ND	1.288 ± 0.057	ND	ND
	64	Luteolin derivative	ND	ND	ND	1.684 ± 0.105	ND	ND	ND
	68	Diosmetin 7-O-rutinoside	ND	ND	ND	ND	0.887 ± 0.004	ND	ND
	76	Vitexin (apigenin-8-C-glu)	0.319 ± 0.010	ND	ND	ND	ND	ND	ND
	87	Vitexin derivative	ND	ND	ND	ND	ND	0.160 ± 0.003	ND
		Σ **Flavone**	0.319 ± 0.010[e]	ND	ND	1.684 ± 0.105[c]	10.863 ± 0.174[a]	0.803 ± 0.010[d]	7.806 ± 0.518[b]

Table S1. *Cont.*

Class	Peak	Phenolic Compounds	*I. crithmoides*	*S. fruticosa*	*S. ramosissima*	*D. crassifolium*	*C. maritimum*	*M. nodiflorum*	*M. crystallinum*
Flavanol	10	Gallocatechin	0.167 ± 0.024	0.239 ± 0.004	0.299 ± 0.001	ND	ND	3.026 ± 0.003	0.182 ± 0.023
	14	Epigallocatechin	ND	ND	ND	0.862 ± 0.107	ND	0.510 ± 0.003	0.519 ± 0.047
	51	Epicatechin hydrate	ND	ND	ND	ND	ND	1.064 ± 0.011	ND
	52	Gallocatechin derivative	0.549 ± 0.035	ND	ND	ND	ND	ND	ND
	55	Epicatechin derivative	ND	11.534 ± 0.164	ND	ND	ND	ND	ND
		**Σ Flavanol**	0.716 ± 0.059[c]	11.773 ± 0.168[a]	0.299 ± 0.001[d]	0.862 ± 0.107[c]	ND	4.600 ± 0.017[b]	0.701 ± 0.007[c]
Flavanone	16	Pinobanksin-3-O-pentanoate	ND	ND	ND	ND	0.731 ± 0.048	ND	1.571 ± 0.273
	31	Pinobanksin-5-methyl ether-3-O-acetate (isomer)	6.382 ± 0.176	ND	ND	ND	ND	ND	ND
	33	Pinocembrin derivative	ND	ND	ND	ND	ND	0.338 ± 0.057	ND
	48	Eriodictyol-O-hexoside	ND	7.071 ± 0.114	ND	ND	ND	2.606 ± 0.011	ND
	49	Erydictiol	ND	ND	ND	ND	ND	8.568 ± 0.090	ND
	92	Pinobanksin-5-methyl ether-3-O-acetate (isomer)	ND	ND	ND	ND	ND	1.553 ± 0.004	ND
		**Σ Flavanone**	6.382 ± 0.176[c]	7.071 ± 0.114[b]	ND	ND	0.731 ± 0.048[e]	13.065 ± 0.162[a]	1.571 ± 0.273[d]
Flavonol	7	Rhamnetin	0.194 ± 0.082	ND	ND	ND	ND	ND	ND
	27	Avicularin	ND	ND	ND	ND	ND	2.422 ± 0.003	ND
	35	Kaempferol derivative	ND	ND	ND	ND	ND	ND	0.576 ± 0.014
	45	Quercetin-3-O-rutinoside	ND	ND	ND	ND	11.854 ± 0.141	ND	ND
	53	Quercetin 3-O-glucoside*	ND	ND	3.338 ± 0.009	ND	5.932 ± 0.042	ND	ND
	58	Isorhamnetin-rutinoside derivative	ND	ND	ND	13.825 ± 0.100	ND	ND	ND
	59	Rhamnetin hexosyl pentoside	ND	27.388 ± 0.127	ND	ND	ND	ND	ND
	65	Isorhamnetin 3-O-robinobioside	ND	20.871 ± 0.143	ND	ND	ND	ND	ND
	69	Quercetin dipentoside	ND	ND	ND	ND	ND	0.133 ± 0.001	ND
	70	Quercetin-3-O-glucoside derivative	ND	ND	ND	ND	ND	ND	0.687 ± 0.066
	72	Quercetin derivative	ND	ND	ND	ND	ND	0.316 ± 0.006	ND
	73	Isorhamnetin-glucoside derivative	ND	ND	ND	7.281 ± 0.103	ND	ND	ND

Table S1. *Cont.*

Class	Peak	Phenolic Compounds	*I. crithmoides*	*S. fruticosa*	*S. ramosissima*	*D. crassifolium*	*C. maritimum*	*M. nodiflorum*	*M. crystallinum*
**Flavonols**	82	Isorhamnetin-3-O-glucoside (dimer)	ND	ND	2.828 ± 0.450	ND	ND	ND	ND
	83	Kaempferol 3-(2''-[glucosyl-(1->3)-rhamnosyl]-6''-rhamnosyl galactoside)	ND	ND	ND	ND	ND	ND	0.594 ± 0.062
		Σ **Flavonol**	0.194 ± 0.082^{g}	48.259 ± 0.270^{a}	6.166 ± 0.459^{d}	21.106 ± 0.203^{b}	17.786 ± 0.183^{c}	2.871 ± 0.010^{e}	1.857 ± 0.142^{f}
**Flavanonol**	36	Dihydroquercetin	ND	6.404 ± 0.792	ND	ND	ND	ND	ND
		Σ **Flavanonol**	ND	6.404 ± 0.792	ND	ND	ND	ND	ND
		Σ **Flavonoids**	7.611 ± 0.065^{f}	73.507 ± 0.224^{a}	6.465 ± 0.153^{g}	23.652 ± 0.103^{c}	29.38 ± 0.068^{b}	21.339 ± 0.015^{d}	11.935 ± 0.125^{e}
**Coumarin**	28	Coumarin glycoside ester	ND	ND	ND	1.458 ± 0.215	ND	ND	ND
**Monocarboxylic acid**	57	Piscidic acid derivative	1.828 ± 0.219	ND	ND	ND	ND	ND	ND
**Gallotannin**	79	Hexahydroxydiphenoyl-Glucose	ND	ND	ND	ND	ND	0.177 ± 0.001	ND
		Σ **Phenolic compounds**	**18.457 ± 0.074^{f}**	**115.886 ± 0.137^{b}**	**118.536 ± 0.174^{b}**	**55.836 ± 0.225^{c}**	**222.757 ± 0.094^{a}**	**28.363 ± 0.010^{e}**	**49.721 ± 0.114^{d}**

The letters (a-g) indicate significant differences between the halophytes using the Tukey's test ($p < 0.05$). ND: Not Detected; LOQ = 0.01 μg compound/g FW.

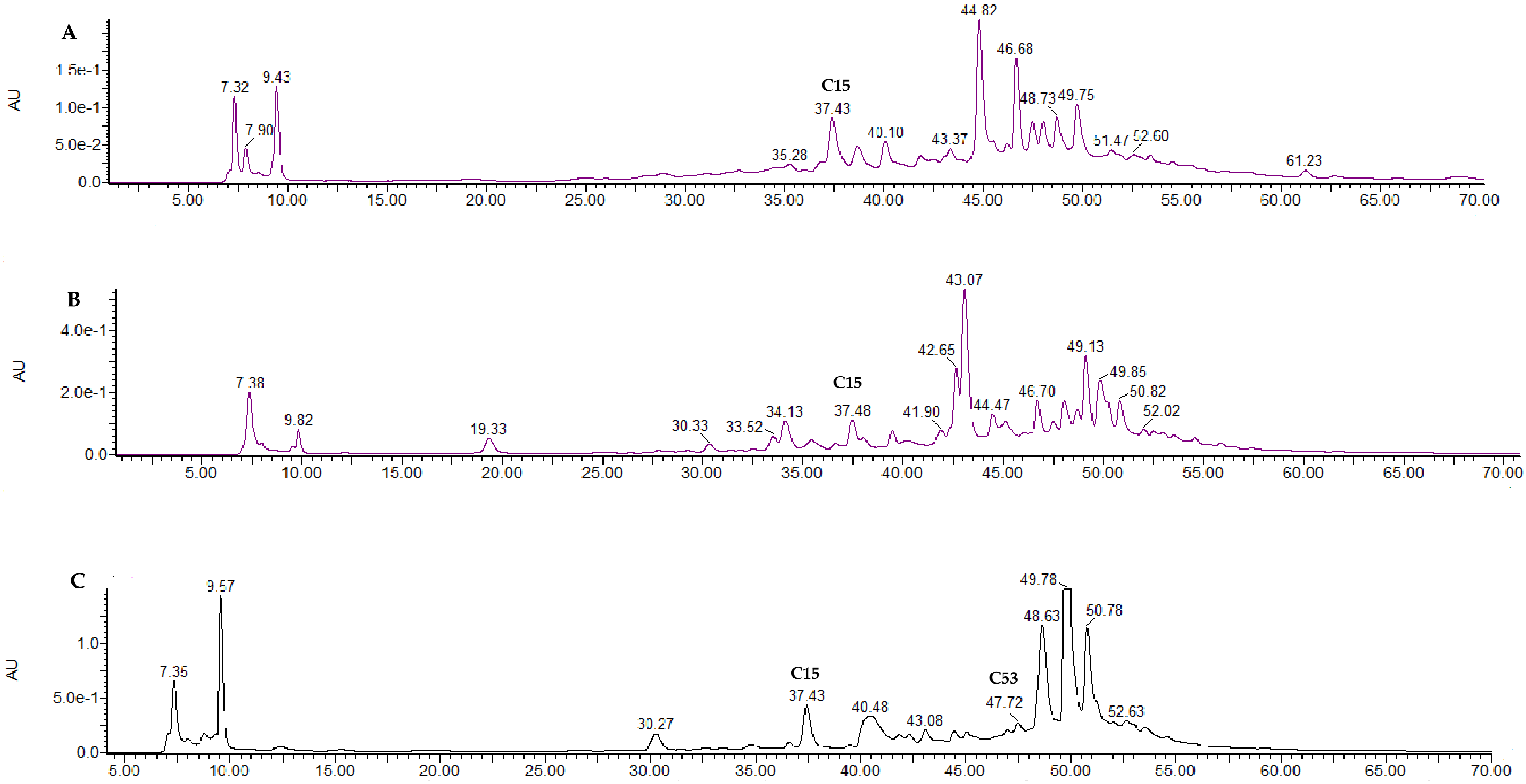

Figure S2. Chromatographic profile at 280 nm by HPLC-DAD-ESI-MS/MS of the halophytes species. *I. crithmoides* (A); *S. fruticosa* (B); *S. ramosissima* (C); *D. crassifolium*(D); *C. maritimum* (E); *M. nodiflorum* (F) and *M. crystallinum* (G).

Figure S2. *Cont.*

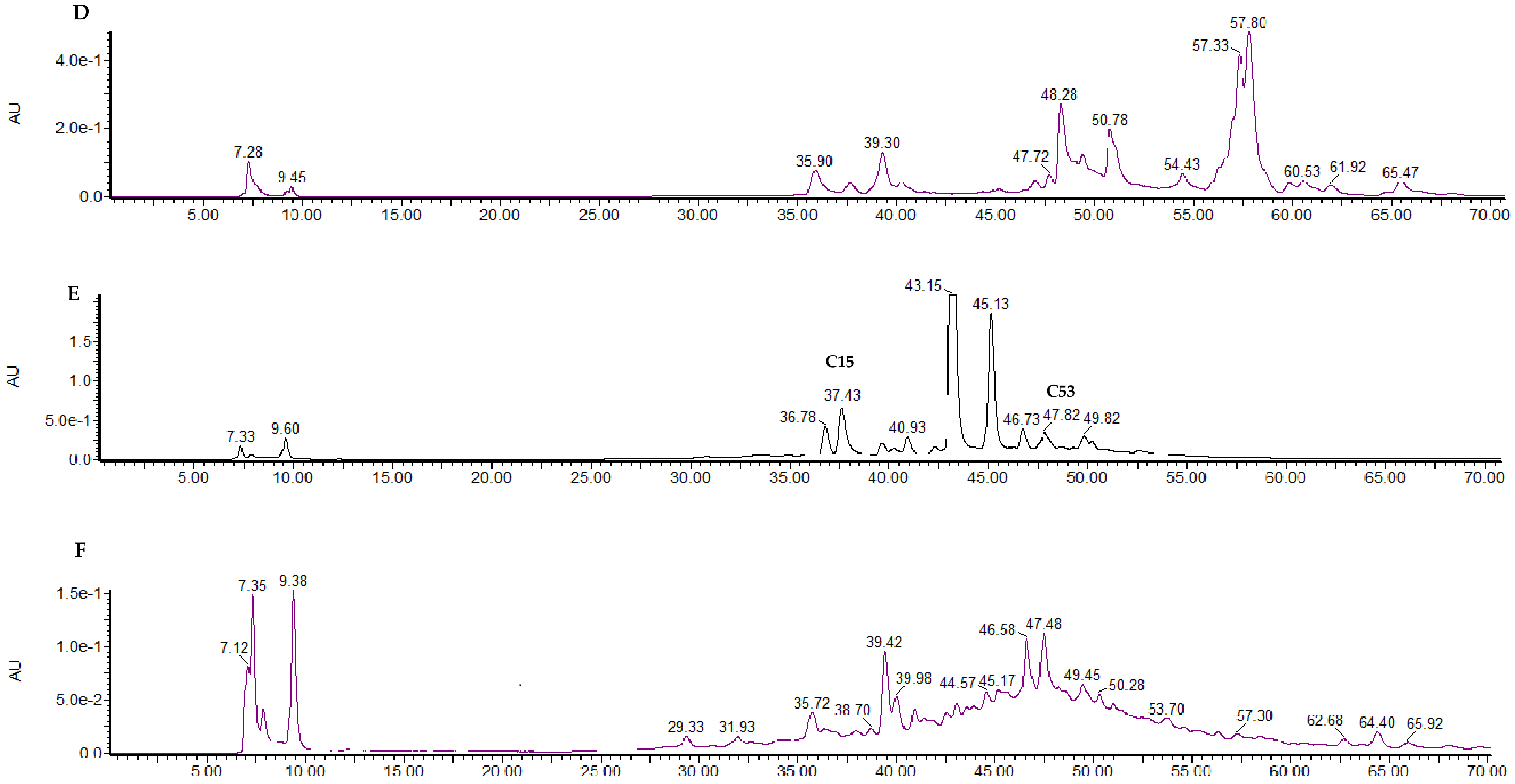

Figure S2. Chromatographic profile at 280 nm by HPLC-DAD-ESI-MS/MS of the halophytes species. *I. crithmoides* (A); *S. fruticosa* (B); *S. ramosissima* (C); *D. crassifolium*(D); *C. maritimum* (E); *M. nodiflorum* (F) and *M. crystallinum* (G).

Figure S2. *Cont.*

G

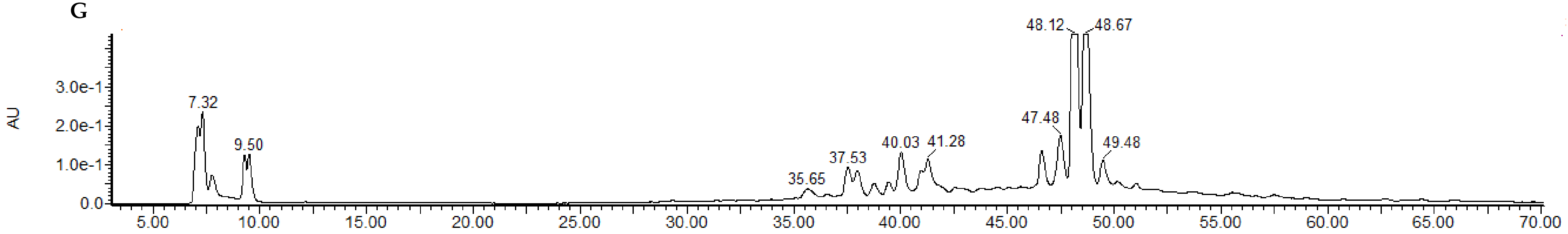

Figure S2. Chromatographic profile at 280 nm by HPLC-DAD-ESI-MS/MS of the halophytes species. *I. crithmoides* (A); *S. fruticosa* (B); *S. ramosissima* (C); *D. crassifolium*(D); *C. maritimum* (E); *M. nodiflorum* (F) and *M. crystallinum* (G).

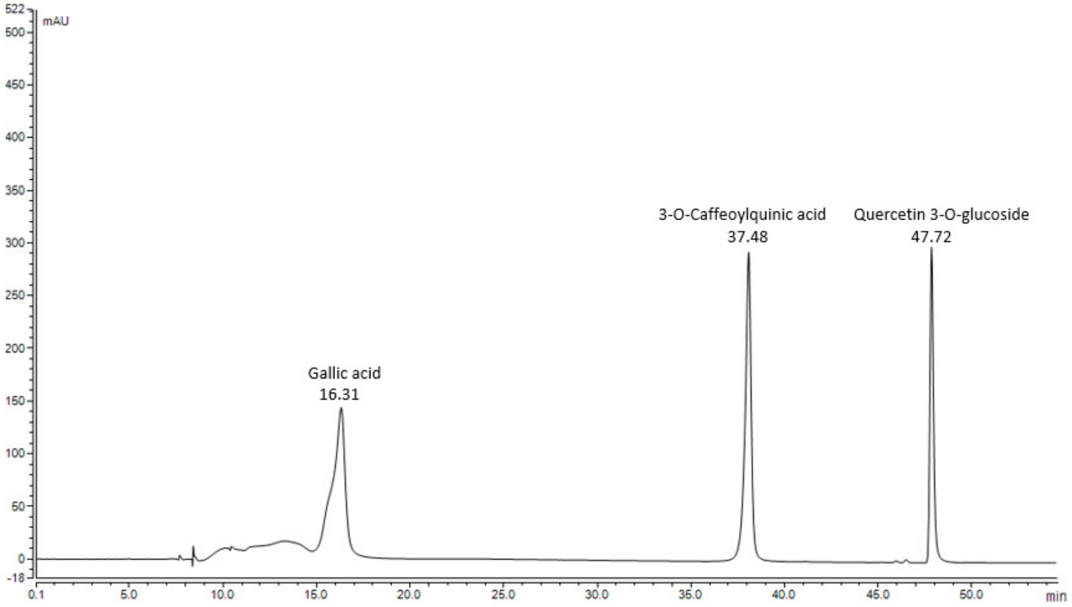

Figure S3. Chromatographic profile at 280 nm by HPLC-DAD of the standards of phenolic compounds: gallic acid, chlorogenic acid (3-O-caffeoylquinic acid) and quercetin-3-glucoside.

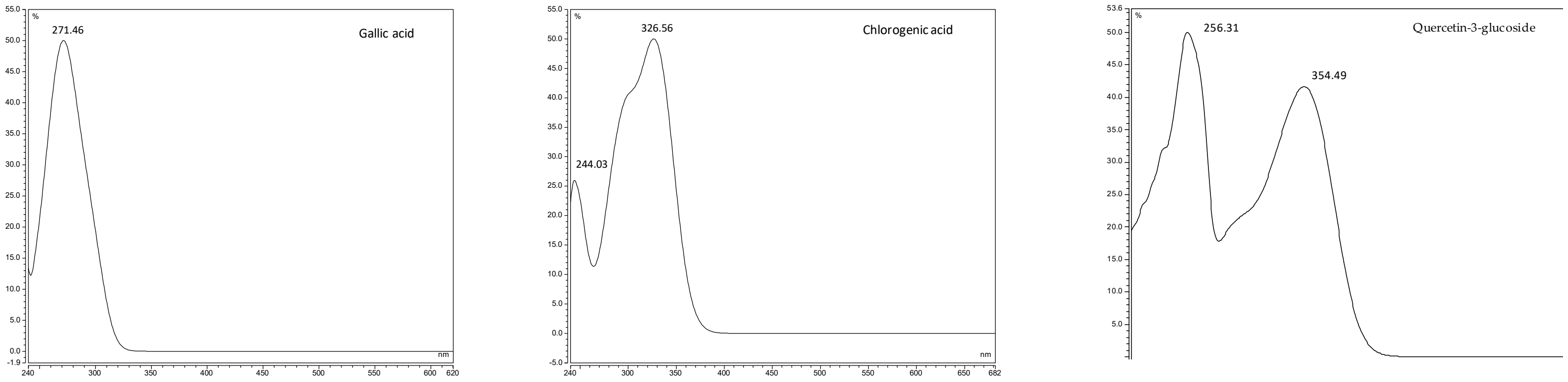

Figure S4. UV-Visible spectra of the phenolic compounds standards: gallic acid, chlorogenic acid (3-O-caffeoylquinic acid) and quercetin-3-glucoside.

Table S2. Characterization parameters (retention time, RT, % of total peak area, tentative identification, chemical classes, odor, calculated/theoretical linear retention index, LRI) of volatile compounds in halophyte species using SPME-GC–MS. The references used to support the tentative identifications were indicated.

Species	Peak	RT (min) [a]	Area%	Tentative Identification	Chemical Classes	Odor Description [b]	Calculated LRI [c]	Theoretical LRI	References
C. maritimum	1	7.24	0.91	3-hexen-1-ol	Alcohol	green, marine, seaweed	860	861	[153-155]
	2	9.55	0.71	alpha-thujene	Terpene	herbal, green, weak earthy	927	934	[156-158]
	3	9.742	1.71	alpha-pinene	Terpene	oily, green	932	933	[159,160]
	4	11.283	17.24	alpha-fenchene	Terpene	fruity, fresh	973	962	[161,162]
	5	11.992	1.01	beta-myrcene	Terpene	herbaceous, sweet	992	992	[158-163]
	6	12.817	0.40	alpha-terpinene	Terpene	resinous	1016	1017	[158,164]
	7	13.125	13.41	*p*-cymene	Terpene	green, fruity, aromatic	1025	1031	[159,165-167]
	8	13.225	0.40	beta-phellandrene	Terpene	NF	1028	1032	[168,169]
	9	13.65	8.87	(*Z*)-beta-ocimene	Terpene	floral, herbaceous	1041	1037	[168,169]
	10	13.992	0.30	(*E*)-beta-ocimene	Terpene	floral, herbaceous	1051	1047	[168,169]
	11	14.358	30.44	*m*-mentha-4,8-diene	Terpene	NF	1062	1081	[168]
	12	15.25	0.30	alpha-terpinolene	Terpene	woody, herbaceous	1088	1086	[164,170]
	13	16.617	0.20	allo-ocimene	Terpene	fresh	1131	1131	[171,172]
	14	18.075	0.20	4-terpineol	Terpene	roasted, woody	1178	1178	[173,174]
	15	19.65	23.59	thymol methyl ether	Terpene	oregano-like, thyme-like, rosemary-like, medicinal	1239	1237	[175-176]
	16	25.242	0.20	alpha-bergamotene	Terpene	wood, warm, tea	1439	1438	[174,178]
	17	27.092	0.10	beta-bisabolene	Terpene	NF	1513	1512	[179]

Table S2. *Cont.*

Species	Peak	RT (min) [a]	Area%	Tentative Identification	Chemical Classes	Odor Description [b]	Calculated LRI [c]	Theoretical LRI	References
I. crithmoides	1	7.00	0.42	3-hexen-1-ol	Alcohol	green, marine, seaweed	860	861	[153-155]
	2	7.53	0.73	1-hexanol	Alcohol	woody, sweet, green, fruity	873	872	[180-182]
	3	9.74	0.52	alpha-pinene	Terpene	oily, green	932	933	[159,160]
	4	10.26	8.34	camphene	Terpene	sweet	946	947	[159,160,183]
	5	11.31	0.73	beta-pinene	Terpene	woody, green, pine-like	974	979	[158,14,183]
	6	12.01	0.21	beta-myrcene	Terpene	herbaceous, sweet	993	992	[158-162]
	7	12.40	17.41	alpha-phellandrene	Terpene	fresh, green	1004	1005	[164,181,183]
	8	12.63	0.10	3-hexen-1-ol acetate	Ester	fruity, floral	1010	1007	[180,184]
	9	12.87	0.31	hexyl acetate	Ester	fruit, herb	1018	1019	[185,186]
	10	13.15	22.94	*p*-cymene	Terpene	green, fruity, aromatic	1026	1031	[159,165-167]
	11	13.24	2.29	limonene	Terpene	pine/chemical, floral/fresh	1029	1029	[187,188]
	12	14.28	0.21	gamma-terpinene	Terpene	green, woody	1059	1059	[159,170,189]
	13	15.25	1.15	alpha-terpinolene	Terpene	woody, herbaceous	1088	1086	[164,170]
	14	17.71	0.31	borneol	Terpene	camphoraceous, earthy	1167	1165	[190,191]
	15	18.08	0.21	4-terpineol	Terpene	roasted, woody	1179	1178	[173,174]
	16	19.78	3.96	thymol methyl ether	Terpene	oregano-like, thyme-like, rose-mary-like, medicinal	1236	1237	[175-177]
	17	20.10	0.73	allyl hexanoate	Ester	sweet, fruity	1248	-	[192]
	18	20.25	17.52	2,4,6-trimethylacetophenone	Ketone	not found	1253	1252	[193]
	19	21.01	0.63	cyclooctyl acetate	Ester	not found	1279	1279	[194]
	20	21.26	19.71	isobornyl acetate	Ester	herb, woody, sweet, minty	1288	1285	[191,192]
	21	21.56	0.21	thymol	Terpene	thyme-like, spicy	1299	1293	[157,195]
	22	23.80	1.15	cadinene	Terpene	herb, burnt, sulphur	1382	1362	[196,197]
	23	24.92	0.21	2-tert-Butyl-1,4-dimethoxybenzene	Terpene	not found	1426	1427	[198]

Table S2. *Cont.*

Species	Peak	RT (min) [a]	Area%	Tentative Identification	Chemical Classes	Odor Description [b]	Calculated LRI [c]	Theoretical LRI	References
D. crassifolium	1	5.23	6.35	hexanal	Aldehyde	herbal, grassy, green	807	801	[154,180,184,199]
	2	7.42	6.69	3-hexen-1-ol	Alcohol	green, marine, seaweed	866	861	[153-155]
	3	7.88	10.37	1-hexanol	Alcohol	woody, sweet, green, fruity	878	872	[180-182]
	4	8.19	1.34	*n*-butyl ether	Ether	not found	887	860	[200]
	5	9.27	6.35	oxime-methoxy-phenyl-	Ester	fishy	916	910	[201,202]
	6	11.97	1.67	2,2,4,6,6-pentamethylheptane	Terpene	stony, dusty	990	991	[203,204]
	7	13.24	9.03	*p*-cymene	Terpene	green, fruity, aromatic	1027	1031	[159,165-167]
	8	15.78	1.00	undecane	Hydrocarbon	not found	1103	1100	[201]
	9	18.83	3.34	dodecane	Hydrocarbon	alkane-like, chemical	1202	1200	[201,205]
	10	19.81	20.74	thymol methyl ether	Terpene	oregano-like, thyme-like, rosemary-like, medicinal	1236	1237	[175-177]
	11	20.23	9.70	2,4,6-trimethylacetophenone	Ketone	not found	1251	1252	[193]
	12	21.52	19.73	isobornyl acetate	Ester	herb, woody, sweet, minty	1287	1285	[191,192]
	13	23.80	3.68	alpha-gurjunene	Terpene	green, herbal	1382	1407	[190,206]

Table S2. *Cont.*

Species	Peak	RT (min) [a]	Area%	Tentative Identification	Chemical Classes	Odor Description [b]	Calculated LRI [c]	Theoretical LRI	References
M. nodiflorum	1	4.342	1.258149	1-pentanol	Alcohol	balsamic	<800	766	[207,208]
	2	5.108	1.372527	octane	Hydrocarbon	Alkane-like	803	800	[209]
	3	7.108	8.578291	3-hexen-1-ol	Alcohol	green, marine, seaweed	858	861	[153-155]
	4	7.683	77.77651	1-hexanol	Alcohol	woody, sweet, green, fruity	873	872	[180-182]
	5	9.542	0.228754	alpha-thujene	Terpene	herbal, green, weak earthy	924	924	[190,210]
	6	11.342	0.343132	1-heptanol	Alcohol	Metallic	973	967	[211]
	7	11.925	2.85943	2-ethylbutanal	Aldehyde	green	989	-	[210]
	8	12.667	0.228754	3-hexen-1-ol acetate	Ester	fruity, floral	1010	1007	[180,184]
	9	12.883	0.800641	hexyl acetate	Ester	fruit, herb	1016	1019	[185,186]
	10	12.983	1.830035	trans-2-hexenyl acetate	Ester	fruity, sweet, herbaceous	1019	1018	[213,214]
	11	13.258	0.343132	limonene	Terpene	pine, chemical, floral, fresh	1028	1029	[188,215]
	12	14.908	0.125815	1-octanol	Alcohol	citrus-like, herbal	1077	1070	[216,217]
	13	15.742	0.137253	linalool	Terpene	pleasant scent, floral	1102	1097	[166,180]
	14	15.9	0.343132	2,6-dimethylcyclohexanol	Alcohol	earthy	1107	1108	[218,219]
	15	18.025	0.457509	1-nonanol	Alcohol	fatty	1176	1176	[220,221]
	16	19.358	0.343132	beta-cyclocitral	Terpene	sweet-tobacco, grape	1221	1220	[215,222]
	17	19.8	1.258149	thymol methyl ether	Terpene	oregano-like, thyme-like, rosemary-like, medicinal	1236	1237	[175-177]
	18	20.225	0.571886	2,4,6-trimethylacetophenone	Ketone	not found	1251	1252	[193]
	19	21.233	0.915018	isobornyl acetate	Ester	herb, woody, sweet, minty	1286	1285	[190,191]
	20	23.8	0.228754	cadinene	Terpene	herb, burnt, sulphur	1382	1362	[196,197]

Table S2. *Cont.*

Species	Peak	RT (min) [a]	Area%	Tentative Identification	Chemical Classes	Odor Description [b]	Calculated LRI [c]	Theoretical LRI	References
S. fruticosa	1	7.41	27.56	2-hexenal	Aldehyde	floral, herbal	856	861	[153,180,184]
	2	7.76	44.04	1-hexanol	Alcohol	woody, sweet, green, fruity	875	872	[180-182]
	3	10.28	1.14	camphene	Terpene	sweet	944	947	[159,160,183]
	4	11.07	4.15	2-hexenoic acid, methyl ester	Ester	not found	966	972	[223]
	5	11.81	0.31	*n*-caproic acid vinyl ester	Ester	not found	986	964	[205]
	6	12.52	3.83	alpha-phellandrene	Terpene	fresh, green	1006	1005	[164,181,183]
	7	12.81	0.21	4-carene	Terpene	sweet, pungent	1014	-	[166]
	8	12.92	0.10	*trans*-2-hexenyl acetate	Ester	fruity, sweet, herbaceous	1017	1018	[213,214]
	9	13.05	2.80	*p*-cymene	Terpene	green, fruity, aromatic	1022	1031	[159,165-167]
	10	13.19	1.66	limonene	Terpene	pine, chemical, floral, fresh	1026	1029	[185,188]
	11	13.81	0.62	ethyl 2-hexenoate	Ester	citrus-like, fatty	1045	1053	[224,225]
	12	15.20	0.41	alpha-terpinolene	Terpene	woody, herbaceous	1085	1086	[164,170]
	13	15.46	0.52	1-nonen-4-ol	Alcohol	cardboard	1093	1094	[226]
	14	20.14	4.46	*p*-(1-butenyl)anisole	Hydrocarbon	not found	1248	1259	[227]
	15	20.92	0.41	3-octen-1-ol, acetate	Ester	toasted nut, smoky, dusty	1275	1279	[184]
	16	21.18	6.63	isobornyl acetate	Ester	herb, woody, sweet, minty	1284	1285	[190,191]
	17	23.71	1.14	cadinene	Terpene	herb, burnt, sulphur	1378	1362	[196,197]

Table S2. *Cont.*

Species	Peak	RT (min) [a]	Area%	Tentative Identification	Chemical Classes	Odor Description [b]	Calculated LRI [c]	Theoretical LRI	References
M. crystallinum	1	4.28	1.78	1-pentanol	Alcohol	balsamic	766	766	[207,208]
	2	5.07	9.89	hexanal	Aldehyde	herbal, grassy, green	802	801	[154,180,184,199,208]
	3	6.93	36.66	2-hexenal	Aldehyde	floral, herbal	853	861	[153,28,32]
	4	7.63	40.33	1-hexanol	Alcohol	woody, sweet, green, fruity	872	872	[180-182]
	5	8.76	0.11	heptanal	Aldehyde	penetrating oily, harsh	902	902	[182,199,228]
	6	9.57	0.67	methoxy-phenyl-oxime	Ester	fishy	924	916	[201,202]
	7	11.55	0.11	3,5,5-trimethyl-2-hexene	Hydrocarbon	not found	979	977	[201]
	8	11.78	0.22	1-octen-3-ol	Alcohol	mushroom	985	980	[154,158,188]
	9	12.30	0.11	3-octanol	Alcohol	mushroom-like, herbal, nutty	994	998	[157,229]
	10	12.69	0.11	*cis*-3-hexenyl acetate	Ester	fruity, green, floral	1011	1007	[180,184,230]
	11	12.92	0.56	hexyl acetate	Ester	fruit, herb	1017	1019	[185,186]
	12	13.02	0.22	*trans*-2-hexenyl acetate	Ester	fruity, sweet, herbaceous	1020	1018	[213,214]
	13	13.13	0.22	*p*-cymene	Terpene	green, fruity, aromatic	1024	1031	[159,165-167]
	14	14.33	0.33	3-carene	Terpene	sweet, strong	1060	-	[231]
	15	14.98	0.03	1-octanol	Alcohol	citrus-like, herbal	1079	1070	[216,217]
	16	15.89	1.22	nonanal	Aldehyde	green, citrus-like	1107	1111	[153,232]
	17	18.31	0.22	octanoic acid	Carboxylic acid	fruity, faint, candy	1185	1173	[233,234]
	18	18.81	0.33	dodecane	Hydrocarbon	alkane-like, chemical	1201	1200	[201,205]
	19	19.80	2.78	thymol methyl ether	Terpene	oregano-like, thyme-like, rose-mary-like, medicinal	1236	1237	[175-177]
	20	20.22	1.22	2,4,6-trimethylacetophenone	Ketone	not found	1251	1252	[193]
	21	21.05	0.44	nonanoic acid	Carboxylic acid	fatty, cheesy	1280	1275	[157]
	22	21.23	2.33	isobornyl acetate	Ester	herb, woody, sweet, minty	1286	1285	[190,191]
	23	22.99	0.09	alpha-cubebene	Terpene	herbaceous, waxy	1351	1352	[235,236]

Table S2. *Cont.*

Species	Peak	RT (min) [a]	Area%	Tentative Identification	Chemical Classes	Odor Description [b]	Calculated LRI [c]	Theoretical LRI	References
S. ramosissima	1	4.99	6.30	hexanal	Aldehyde	herbal, grassy, green	800	801	[154,180,184,199,208]
	2	7.04	45.65	2-hexenal	Aldehyde	floral, herbal	856	861	[153,180,184]
	3	7.78	47.68	octanal	Aldehyde	green, citrusy, lemon	976	1001	[208,237,238]
	4	11.58	0.06	*n*-caproic acid vinyl ester	Ester	not found	979	964	[205]
	5	13.81	0.10	benzeneacetaldehyde	Aldehyde	fresh, floral	1044	1043	[232,239]
	6	15.57	0.10	1-nonen-4-ol	Alcohol	cardboard	1096	1094	[226,240]
	7	15.88	0.10	2,6-dimethyl-cyclohexanol	Alcohol	earthy	1106	1108	[218,219]

[a] Mean of the retention times in the chromatograms of the fresh halophyte species (n=2). [b] Described odor descriptions. [c] Mean calculated of the linear retention indexes. NF – Not found.

References

153. Lin, L.; Zhuang, M.; Lei, F.; Yang, B.; Zhao, M. GC/MS analysis of volatiles obtained by headspace solid-phase microextraction and simultaneous–distillation extraction from *Rabdosia serra* (MAXIM.) HARA leaf and stem. *Food Chemistry* **2013,** *136* (2), 555-562.
154. Radulović, N.; Blagojević, P.; Palić, R. Comparative study of the leaf volatiles of *Arctostaphylos uva-ursi* (L.) Spreng. and *Vaccinium vitis-idaea* L. (Ericaceae). *Molecules* **2010**, *15(9)*, 6168-6185.
155. Aparicio, R.; Morales, M.T. Characterization of Olive Ripeness by Green Aroma Compounds of Virgin Olive Oil. *J. Agric. Food Chem.* **1998**, *46*, 1116–1122.
156. Costa, R.; d'Acampora Zellner, B.; Crupi, M. L.; Fina, M. R. D.; Valentino, M. R.; Dugo, P.; Dugo, G.; Mondello, L. GC–MS, GC–O and enantio–GC investigation of the essential oil of *Tarchonanthus camphoratus* L. *Flavour and Fragrance Journal* **2008,** *23* (1), 40-48.
157. Jirovetz, L.; Smith, D.; Buchbauer, G. Aroma Compound Analysis of *Eruca sativa* (*Brassicaceae*) SPME Headspace Leaf Samples Using GC, GC–MS, and Olfactometry. *Journal of Agricultural and Food Chemistry* **2002,** *50* (16), 4643-4646.
158. Aligiannis, N.; Kalpoutzakis, E.; Mitaku, S.; Chinou, I.B. Composition and antimicrobial activity of the essential oils of two *Origanum* species. *J. Agric. Food Chem.* **2001**, *49*, 4168–4170.
159. Hazzit, M.; Baaliouamer, A.; Faleiro, M. L.; Miguel, M. Graça. Composition of the essential oils of *Thymus* and *Origanum* species from Algeria and their antioxidant and antimicrobial activities. *Journal of Agricultural and Food Chemistry* **2006**, *54(17)*, 6314–6321.
160. Stashenko, E.E.; Martínez, J.R.; Ruíz, C.A.; Arias, G.; Durán, C.; Salgar, W.; Cala, M. *Lippia origanoides* chemotype differentiation based on essential oil GC-MS and principal component analysis. *J. Sep. Sci.* **2010**, 33, 93–103.
161. Karabagias, I.K.; Karabagias, V.K.; Riganakos, K.A. Physico-Chemical Parameters, Phenolic Profile, *In Vitro* Antioxidant Activity and Volatile Compounds of Ladastacho (*Lavandula stoechas*) from the Region of Saidona. *Antioxidants* **2019**, *8*, 80.
162. Gerretzen, J.; Buydens, L. M. C.; Tromp – van den Beukel, A. O.; Koussissi, E.; Brouwer, E. R.; Jansen, J. J.; Szymańska, E. A novel approach for analyzing gas chromatography-mass spectrometry/olfactometry data. *Chemometrics and Intelligent Laboratory Systems* **2015**, 146, 290-296.
163. Nguir, A.; Besbes, M.; Ben Jannet, H.; Flamini, G.; harzallah-Skhiri, F.; Hamza, M. H. Chemical Composition, Antioxidant and Anti-acetylcholinesterase activities of Tunisian *Crithmum maritimum* L. Essential oils. *Mediterranean Journal of Chemistry* **2011**, 1, 173-179.
164. Minh Tu, N. T.; Onishi, Y.; Choi, H.-S.; Kondo, Y.; Bassore, S. M.; Ukeda, H.; Sawamura, M. Characteristic Odor Components of *Citrus sphaerocarpa* Tanaka (Kabosu) Cold-Pressed Peel Oil. *Journal of Agricultural and Food Chemistry* **2002,** *50* (10), 2908-2913.
165. Vasta, V.; D'Alessandro, A. G.; Priolo, A.; Petrotos, K.; Martemucci, G. Volatile compound profile of ewe's milk and meat of their suckling lambs in relation to pasture vs. indoor feeding system. *Small Ruminant Research* **2012,** *105* (1), 16-21.
166. Yi, L.; Dong, N.; Liu, S.; Yi, Z.; Zhang, Y. Chemical features of *Pericarpium Citri* Reticulatae and *Pericarpium Citri* Reticulatae Viride revealed by GC–MS metabolomics analysis. *Food Chemistry* **2015,** *186*, 192-199.

167. Pino, J.A.; Quijano, C.E. Estudo de compostos voláteis de ameixa (*prunus domestica* L. cv. horvin) e estimativa da sua contribuição ao aroma. *Cienc. Tecnol. Aliment.* **2012**, *32*, 76–83.
168. Figuérédo, G.; Chalchat, J.-C.; Petrovic, S.; Maksimovic, Z.; Gorunovic, M.; Boza, P.; Radic, J. Composition of Essential Oils of Flowers, Leaves, Stems and Rhizome of *Peucedanum officinale* L. (*Apiaceae*). Journal of Essential Oil Research **2009**, 21(2), 123-126.
169. Pino, J. A.; Marbot, R.; Vázquez, C. Characterization of Volatiles in Strawberry Guava (*Psidium cattleianum* Sabine) Fruit. *Journal of Agricultural and Food Chemistry* **2001,** *49* (12), 5883-5887.
170. Wang, Y.; Finn, C.; Qian, M.C. Impact of growing environment on Chickasaw blackberry (*Rubus* L) aroma evaluated by gas chromatography olfactometry dilution analysis. *J. Agric. Food Chem.* **2005**, *53*, 3563–3571.
171. Flamini, G.; Tebano, M.; Cioni, P.L. Volatiles emission patterns of different plant organs and pollen of *Citrus limon*. *Anal. Chim. Acta* **2007**, *589*, 120–124.
172. Cai, X.; Mai, R.-Z.; Zou, J.-J.; Zhang, H.-Y.; Zeng, X.-L.; Zheng, R.-R.; Wang, C.-Y. Analysis of aroma-active compounds in three sweet osmanthus (*Osmanthus fragrans*) cultivars by GC-olfactometry and GC-MS. *Journal of Zhejiang University-Sci B* **2014,** *15* (7), 638-648.
173. Xie, J.; Sun, B.; Zheng, F.; Wang, S. Volatile flavor constituents in roasted pork of Mini-pig. *Food Chemistry* **2008,** *109* (3), 506-514.
174. Mustapha, M. B.; Zardi-Bergaoui, A.; Chaieb, I.; Flamini, G.; Ascrizzi, R.; Jannet, H. B. Chemical Composition and Insecticidal Activity of *Crithmum Maritimum* L. Essential Oil against Stored-Product Beetle *Tribolium Castaneum*. *Chemistry & Biodiversity* **2020,** *17* (3), e1900552.
175. Flamini, G.; Cioni, P. L.; Morelli, I. Differences in the Fragrances of Pollen and Different Floral Parts of Male and Female Flowers of *Laurus nobilis*. *Journal of Agricultural and Food Chemistry* **2002,** *50* (16), 4647-4652.
176. Hudaib, M.; Speroni, E.; Di Pietra, A. M.; Cavrini, V. GC/MS evaluation of thyme (*Thymus vulgaris* L.) oil composition and variations during the vegetative cycle. *Journal of Pharmaceutical and Biomedical Analysis* **2002,** *29* (4), 691-700.
177. Schreiner, L.; Bauer, J.; Ortner, E.; Buettner, A. Structure–Odor Activity Studies on Derivatives of Aromatic and Oxygenated Monoterpenoids Synthesized by Modifying *p*-Cymene. *Journal of Natural Products* **2020,** *83* (4), 834-842.
178. Laokuldilok, N.; Utama-ang, N.; Kopermsub, P.; Thakeow, P. Characterization of Odor Active Compounds of Fresh and Dried Turmeric by Gas Chromatography-Mass Spectrometry, Gas Chromatography Olfactometry and Sensory Evaluation. *FAB J* **2017**, 3, 216-230.
179. Stoppacher, N; Kluger, B; Zeilinger, S; Krska, R; Schuhmacher, R. Identification and profiling of volatile metabolites of the biocontrol fungus *Trichoderma atroviride* by HS-SPME-GC-MS. *J Microbiol Methods* **2010,** 81(2), 187-193.
180. Sampaio, T.S.; Nogueira, P.C.L. Volatile components of mangaba fruit (*Hancornia speciosa* Gomes) at three stages of maturity. *Food Chem.* **2006**, 95, 606–610
181. Quijano, C.E.; Linares, D.; Pino, J.A. Changes in volatile compounds of fermented cereza agria [*Phyllanthus acidus* (L.) Skeels] fruit. *Flavour Fragr.* J. **2007**, 22, 392–394.

182. Lykomitros, D.; Fogliano, V.; Capuano, E. Flavor of roasted peanuts (*Arachis hypogaea*) — Part II: Correlation of volatile compounds to sensory characteristics. *Food Research International* **2016**, 89, 870–881.

183. Telci, I.; Demirtas, I.; Sahin, A. Variation in plant properties and essential oil composition of sweet fennel (*Foeniculum vulgare* Mill.) fruits during stages of maturity. *Industrial Crops and Products* **2009**, 30 (1), 126-130.

184. Jordán, M. J.; Tandon, K.; Shaw, P. E.; Goodner, K. L., Aromatic Profile of Aqueous Banana Essence and Banana Fruit by Gas Chromatography−Mass Spectrometry (GC-MS) and Gas Chromatography−Olfactometry (GC-O). *Journal of Agricultural and Food Chemistry* **2001**, 49 (10), 4813-4817.

185. Gürbüz, O.; Rouseff, J. M.; Rouseff, R. L., Comparison of Aroma Volatiles in Commercial Merlot and Cabernet Sauvignon Wines Using Gas Chromatography−Olfactometry and Gas Chromatography−Mass Spectrometry. *Journal of Agricultural and Food Chemistry* **2006**, 54 (11), 3990-3996.

186. Cardeal, Z.L., Gomes da Silva, M.D., Marriott, P.J. Comprehensive two-dimensional gas chromatography/mass spectrometric analysis of pepper volatiles. *Rapid Commun Mass Spectrom*. **2006**;20(19), 2823-2836.

187. Beaulieu, J. C.; Grimm, C. C. Identification of Volatile Compounds in Cantaloupe at Various Developmental Stages Using Solid Phase Microextraction. *Journal of Agricultural and Food Chemistry* **2001,** *49* (3), 1345-1352.

188. Varlet, V.; Knockaert, C.; Prost, C.; Serot, T. Comparison of Odor-Active Volatile Compounds of Fresh and Smoked Salmon. *Journal of Agricultural and Food Chemistry* **2006,** *54* (9), 3391-3401.

189. Purcaro, G.; Tranchida, P.Q.; Jacques, R.A.; Caramão, E.B.; Moret, S.; Conte, L.; Dugo, P.; Dugo, G.; Mondello, L. Characterization of the yerba mate (*Ilex paraguariensis*) volatile fraction using solid-phase microextraction-comprehensive 2-D GC-MS. *J. Sep. Science* **2009**, 32, 3755-3763.

190. Jirovetz, L.; Buchbauer, G.; Denkova, Z.; Slavchev, A.; Stoyanova, A.; Schmidt, E. Chemical composition, antimicrobial activities and odor descriptions of various *Salvia sp*. and *Thuja sp*. essential oils. *Ernährung/nutrition* **2006,** *30*, 152-159.

191. Nezhadali, A; Shirvan, B.Z. Separation, Identification and Determination of Volatile Compounds of *Ziziphora persica Bunge* Using HS-SPME/GC-MS. *International Journal of Environmental Science and Development* **2010**, 1(2), 115-118.

192. Niu, Y.; Wang, P.; Xiao, Q.; Xiao, Z.; Mao, H.; Zhang, J. Characterization of Odor-Active Volatiles and Odor Contribution Based on Binary Interaction Effects in Mango and Vodka Cocktail. *Molecules* **2020**, *25*, 1083.

193. Jeribi, C.; Karoui, I. J.; Benhassine, D.; Abderrabba, M. Chemical Composition of *Cardopatium corymbosum* Leaves Essential Oil. *Journal of Essential Oil Bearing Plants* **2016,** *19* (6), 1471-1477.

194. Villa-Ruano, N.; Pacheco-Hernández, Y.; Cruz-Durán, R.; Lozoya-Gloria, E. Volatiles and seasonal variation of the essential oil composition from the leaves of *Clinopodium macrostemum* var. *laevigatum* and its biological activities. *Industrial Crops and Products* **2015,** *77*, 741-747.

195. Mockute, D.; Bernotiene, G. The Main Citral−Geraniol and Carvacrol Chemotypes of the Essential Oil of *Thymus pulegioides* L. Growing Wild in Vilnius District (Lithuania). *Journal of Agricultural and Food Chemistry* **1999,** *47* (9), 3787-3790.

196.Moon, S.-Y.; Cliff, M. A.; Li-Chan, E. C. Y. Odour-active components of simulated beef flavour analysed by solid phase microextraction and gas chromatography–mass spectrometry and –olfactometry. *Food Research International* **2006,** *39* (3), 294-308.

197.Giorgi, A.; Panseri, S.; Nanayakkara, N.N.MC.; Chiesa, L. HS-SPME-GC/MS analysis of the volatile compounds of Achillea collina: Evaluation of the emissions fingerprint induced by *Myzus persicae* infestation. *J. Plant Biol.* **2012**, 55, 251–260.

198.Kokoska, L., Urbanova, K., Kloucek, P., Nedorostova, L., Polesna, L., Malik, J., Jiros, P., Havlik, J., Vadlejch, J. and Valterova, I. Essential Oils in the *Ranunculaceae* Family: Chemical Composition of Hydrodistilled Oils from *Consolida regalis, Delphinium elatum, Nigella hispanica*, and *N. nigellastrum* Seeds. *Chemistry & Biodiversity* **2012**, 9, 151-161.

199.Cho, I. H.; Lee, S. Min; Kim, S. Y.; Choi, H.; Kim, K.-O.; Kim, Y.-S. (2007). Differentiation of aroma characteristics of pine-mushrooms (*Tricholoma matsutake* Sing.) of different grades using gas chromatography−olfactometry and sensory analysis. *Journal of Agricultural and Food Chemistry* **2007**, *55(6)*, 2323–2328.

200.Gong, W.-c.; Chen, G.; Liu, C.-q.; Dunn, B. L.; Sun, W.-b. Comparison of floral scent between and within *Buddleja fallowiana* and *Buddleja officinalis* (*Scrophulariaceae*). *Biochemical Systematics and Ecology* **2014,** *55*, 322-328.

201.Ma, R.; Liu, X.; Tian, H.; Han, B.; Li, Y.; Tang, C.; Zhu, K.; Li, C.; Meng, Y. Odor-active volatile compounds profile of triploid rainbow trout with different marketable sizes. *Aquaculture Reports* **2020,** *17*, 100312.

202.Wang, P.; Ma, X.; Wang, W.; Xu, D.; Zhang, X.; Zhang, J.; Sun, Y. Characterization of flavor fingerprinting of red sufu during fermentation and the comparison of volatiles of typical products. *Food Science and Human Wellness* **2019,** *8* (4), 375-384.

203.Tontul, I.; Torun, M.; Dincer, C.; Sahin-Nadeem, H.; Topuz, A.; Turna, T.; Ozdemir, F. Comparative study on volatile compounds in Turkish green tea powder: Impact of tea clone, shading level and shooting period. *Food Research International* **2013,** *53* (2), 744-750.

204.Villberg, K.; Veijanen, A.; Gustafsson, I.; Wickström, K. Analysis of odour and taste problems in high-density polyethene. *Journal of Chromatography A* **1997,** *791* (1), 213-219.

205.Guo, X.; Ho, C.-T.; Wan, X.; Zhu, H.; Liu, Q.; Wen, Z. Changes of volatile compounds and odor profiles in Wuyi rock tea during processing. *Food Chemistry* **2021,** *341*, 128230.

206.Villa, C.; Trucchi, B.; Bertoli, A.; Pistelli, L.; Parodi, A.; Bassi, A. M.; Ruffoni, B. *Salvia somalensis* essential oil as a potential cosmetic ingredient: solvent-free microwave extraction, hydrodistillation, GC–MS analysis, odour evaluation and *in vitro* cytotoxicity assays. *International Journal of Cosmetic Science* **2009,** *31* (1), 55-61.

207.Liu, J.; Tang, X.; Zhang, Y.; Zhao, W. Determination of the Volatile Composition in Brown Millet, Milled Millet and Millet Bran by Gas Chromatography/Mass Spectrometry. *Molecules* **2012**, *17*,2271-2282.

208.Zhu, J.; Chen, F.; Wang, L.; Niu, Y.; Yu, D.; Shu, C.; Chen, H.; Wang, H.; Xiao, Z. Comparison of Aroma-Active Volatiles in Oolong Tea Infusions Using GC–Olfactometry, GC–FPD, and GC–MS. *Journal of Agricultural and Food Chemistry* **2015,** *63* (34), 7499-7510.

209.Huang, X.-H.; Zheng, X.; Chen, Z.-H.; Zhang, Y.-Y.; Du, M.; Dong, X.-P.; Qin, L.; Zhu, B.-W. Fresh and grilled eel volatile fingerprinting by e-Nose, GC-O, GC–MS and GC × GC-QTOF combined with purge and trap and solvent-assisted flavor evaporation. *Food Research International* **2019,** *115*, 32-43.

210. Angioni, A.; Barra, A.; Coroneo, V.; Dessi, S.; Cabras, P. Chemical composition, seasonal variability, and antifungal activity of *Lavandula stoechas* L. ssp. *stoechas* Essential Oils from Stem/Leaves and Flowers. *J. Agric. Food Chem.* **2006**, *54(12)*, 4364–4370.

211.Lu, Q.; Liu, F.; Bao, J. Volatile components of American silver carp analyzed by electronic nose and MMSE-GC-MS-O. *Journal of Food Biochemistry* **2019,** *43* (11), e13006.

212.Kishimoto, N.; Kashiwagi, A. Prediction of Specific Odor Markers in Oil from Olive Fruit Infested with Olive Scale Using an Electronic Nose. *IEEE International Symposium on Olfaction and Electronic Nose (ISOEN)* ***2019***, Fukuoka, Japan, 1-3.

213.Kiatbenjakul, P.; Intarapichet, K.-O.; Cadwallader, K. R. Characterization of potent odorants in male giant water bug (*Lethocerus indicus* Lep. and Serv.), an important edible insect of Southeast Asia. *Food Chemistry* **2015,** *168*, 639-647.

214.Mahattanatawee, K.; Luanphaisarnnont, T.; Rouseff, R. Comparison of Aroma Character Impact Volatiles of Thummong Leaves (*Litsea petiolata* Hook. f.), Mangdana Water Beetle (*Lethocerus indicus*), and a Commercial Product as Flavoring Agents in Thai Traditional Cooking. *Journal of Agricultural and Food Chemistry* **2018,** *66* (10), 2480-2484.

215.. Pino, J. A.; Mesa, J.; Muñoz, Y.; Martí, M. P.; Marbot, R. Volatile Components from Mango (*Mangifera indica* L.) Cultivars. *J. Agric. Food Che*m. **2005**, *53*, 2213–2223.

216.Nóbrega, I. C. C.; Ataíde, C. S.; Moura, O. M.; Livera, A. V.; Menezes, P. H. Volatile constituents of cooked bullfrog (*Rana catesbeiana*) legs. *Food Chemistry* **2007,** *102* (1), 186-191.

217.Cuevas, F. J.; Moreno-Rojas, J. M.; Ruiz-Moreno, M. J. Assessing a traceability technique in fresh oranges (*Citrus sinensis* L. Osbeck) with an HS-SPME-GC-MS method. Towards a volatile characterisation of organic oranges. *Food Chemistry* **2017,** *221*, 1930-1938.

218.Baião, L. F.; Oliveira, A. S.; Gonçalves, A.; Guedes de Pinho, P.; Valente, L. M. P.; Cunha, L. M. Analysis of volatile compounds in *Paracentrotus lividus* by HS-SPME/GS-MS and relation to its sensorial properties. *LWT* **2020,** *130*, 109629.

219.Bianchi, G.; Falcinelli, B.; Tosti, G.; Bocci, L.; Benincasa, P. Taste quality traits and volatile profiles of sprouts and wheatgrass from hulled and non-hulled *Triticum* species. *Journal of Food Biochemistry* **2019,** *43* (7), e12869.

220.Yang, D. S.; Shewfelt, R. L.; Lee, K.-S.; Kays, S. J. Comparison of Odor-Active Compounds from Six Distinctly Different Rice Flavor Types. *Journal of Agricultural and Food Chemistry* **2008,** *56* (8), 2780-2787.

221.Cai, L.; Ao, Z.; Tang, T.; Tong, F.; Wei, Z.; Yang, F.; Shu, Y.; Liu, S.; Mai, K. Characterization of difference in muscle volatile compounds between triploid and diploid crucian carp. *Aquaculture Reports* **2021,** *20*, 100641.

222.Zhang, K.; Lin, T. F.; Zhang, T.; Li, C.; Gao, N. Characterization of typical taste and odor compounds formed by *Microcystis aeruginosa*. *Journal of Environmental Sciences* **2013,** *25* (8), 1539-1548.

223.Li, Q.; Shi, X.; Zhao, Q.; Cui, Y.; Ouyang, J.; Xu, F. Effect of cooking methods on nutritional quality and volatile compounds of Chinese chestnut (*Castanea mollissima* Blume). *Food Chemistry* **2016,** *201*, 80-86.

224.Yang, C.; Wang, Y.; Liang, Z.; Fan, P.; Wu, B.; Yang, L.; Wang, Y.; Li, S. Volatiles of grape berries evaluated at the germplasm level by headspace-SPME with GC–MS. *Food Chemistry* **2009,** *114* (3), 1106-1114.

225.Chen, J. L.; Wu, J. H.; Wang, Q.; Deng, H.; Hu, X. S. Changes in the Volatile Compounds and Chemical and Physical Properties of Kuerle Fragrant Pear (*Pyrus serotina* Reld) during Storage. *Journal of Agricultural and Food Chemistry* **2006,** *54* (23), 8842-8847.

226.Radulović, N.; Stojanović, G.; Milovanovic, V.; Dokovic, D.; Randjelovic, V. Volatile constituents of *Equisetum fluviatile* L. *Journal of Essential Oil Research* **2008,** *20*, 437-441.

227.Velasco-Negueruela, A.; Pérez-Alonso, M. J.; de Paz, P. L. P.; Palá-Paúl, J.; Sanz, J. Analysis by gas chromatography–mass spectrometry of the essential oils from the aerial parts of *Pimpinella anagodendron* Bolle and *Pimpinella rupicola* Svent., two endemic species to the Canary Islands, Spain. *Journal of Chromatography A* **2005,** *1095* (1), 180-184.

228.García-Aguilar, L.; Rojas-Molina, A.; Ibarra-Alvarado, C.; Rojas-Molina, J.I.; Vázquez-Landaverde, P.A.; Luna-Vázquez, F.J.; Zavala-Sánchez, M.A. Nutritional Value and Volatile Compounds of Black Cherry (*Prunus serotina*) Seeds. *Molecules* **2015**, *20,* 3479-3495.

229.Costa, R.; Tedone, L.; De Grazia, S.; Dugo, P.; Mondello, L. Multiple headspace-solid-phase microextraction: An application to quantification of mushroom volatiles. *Analytica Chimica Acta* **2013,** *770*, 1-6.

230.Pino, J. A.; Trujillo, R. Characterization of odour-active compounds of sour guava (*Psidium acidum*[DC.]Landrum) fruit by gas chromatography-olfactometry and odour activity value. *Flavour and Fragrance Journal* **2021,** *36* (2), 207-212.

231.Ali, S. B.; Ghatak, B.; Gupta, S. D.; Debabhuti, N.; Chakraborty, P.; Sharma, P.; Ghosh, A.; Tudu, B.; Mitra, S.; Sarkar, M. P.; Bhattacharyya, N.; Bandyopadhyay, R. Detection of 3-Carene in mango using a quartz crystal microbalance sensor. *Sensors and Actuators B: Chemical* **2016,** *230*, 791-800.

232.Selli, S.; Cayhan, G. G. Analysis of volatile compounds of wild gilthead sea bream (*Sparus aurata*) by simultaneous distillation–extraction (SDE) and GC–MS. *Microchemical Journal* **2009,** *93* (2), 232-235.

233.Villière, A.; Arvisenet, G.; Lethuaut, L.; Prost, C.; Sérot, T. Selection of a representative extraction method for the analysis of odourant volatile composition of French cider by GC–MS–O and GC×GC–TOF-MS. *Food Chemistry* **2012,** *131* (4), 1561-1568.

234.Shen, X.; Chen, W.; Zheng, Y.; Lei, X.; Tang, M.; Wang, H.; Song, F. Chemical composition, antibacterial and antioxidant activities of hydrosols from different parts *of Areca catechu* L. and *Cocos nucifera* L. *Industrial Crops and Products* **2017,** *96*, 110-119.

235.Dussort, P.; Deprêtre, N.; Bou-Maroun, E.; Fant, C.; Guichard, E.; Brunerie, P.; Le Fur, Y.; Le Quéré, J. L. An original approach for gas chromatography-olfactometry detection frequency analysis: Application to gin. *Food Research International* **2012,** *49* (1), 253-262.

236. Miranda, R. F.; de Paula, M. M.; da Costa, G. M.; Barão, C. E.; da Silva, A. C. R.; Raices, R. S. L.; Gomes, R. G.; Pimentel, T. C. Orange juice added with *L. casei*: is there an impact of the probiotic addition methodology on the quality parameters? *LWT* **2019,** *106*, 186-193.
237. Amanpour, A.; Kelebek, H.; Kesen, S.; Selli, S. Characterization of Aroma-Active Compounds in Iranian cv. Mari Olive Oil by Aroma Extract Dilution Analysis and GC–MS-Olfactometry. *Journal of the American Oil Chemists' Society* **2016,** *93* (12), 1595-1603.
238. Evstatieva, L.; Todorova, M.; Antonova, D.; Staneva J. Chemical composition of the essential oils of *Rhodiola rosea* L. of three different origins. *Pharmacogn Mag*. **2010**, 6(24), 256-258.
239. Fu, M.; Shen, X.; Peng, H.; Zhou, Q.; Yun, J.; Sun, Y.; Ho, C.-T.; Cai, H.; Hou, R. Identification of rancidity markers in roasted sunflower seeds produced from raw materials stored for different periods of time. *LWT* **2020,** *118*, 108721.
240. Matsuishi, M.; Kume, J.; Itou, Y.; Takahashi, M.; Arai, M.; Nagatomi, H.; Watanabe, K.; Hayase, F.; Okitani, A. Aroma components of Wagyu beef and imported beef. *Nihon Chikusan Gakkaiho* **2004,** *75* (3), 409-415.
